# Nitrile Substituents at the Conjugated Dipyridophenazine Moiety as Infrared Redox Markers in Electrochemically Reduced Heteroleptic Ru(II) Polypyridyl Complexes

**DOI:** 10.1021/acs.inorgchem.3c03484

**Published:** 2024-01-23

**Authors:** Elizabeth Sumner, Martin Pižl, Kane T. McQuaid, František Hartl

**Affiliations:** † Department of Chemistry, University of Reading, Whiteknights, Reading RG6 6DX, U.K.; ‡ Department of Inorganic Chemistry, University of Chemistry and Technology Prague, Technická 5, Prague 6 166 28, Czech Republic

## Abstract

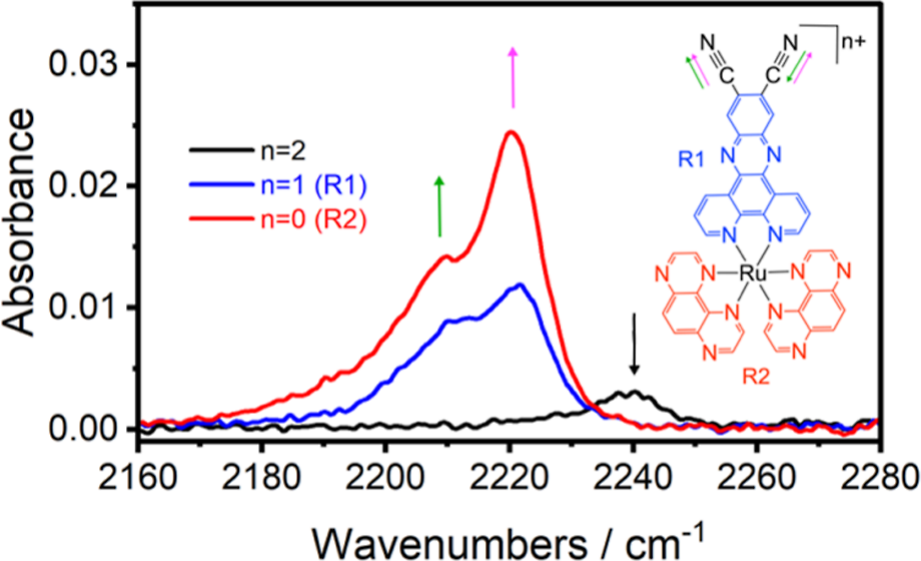

Ruthenium(II) complexes [Ru(tap)_2_(NN)]^2+^ (tap = 1,4,5,8-tetraazaphenanthrene, NN = 11-cyano-dipyrido[3,2-*a*:2′,3′-*c*]phenazine (11-CN-dppz) and 11,12-dicyano-dipyrido[3,2-*a*:2′,3′-*c*]phenazine (11,12-CN-dppz)) feature the C≡N groups as infrared (IR)-active redox markers. They were studied by cyclic voltammetry, UV–vis, and IR spectroelectrochemistry (SEC), and density functional theory calculations to assign the four 1e^–^ reduction waves R1–R4 observed in dichloromethane. Generally, the NN ligands are reduced first (R1). For [Ru(tap)_2_(11,12-CN-dppz)]^2+^, R1 is sufficiently separated from R2 and delocalized over both tap ligands. Accordingly, IR SEC conducted at R1 shows a large red shift of the ν_s,as_(C≡N) modes by −18/–28 cm^–1^, accompanied by a 4-fold enhancement of the ν_s_(C≡N) intensity, comparably with reference data for free 11,12-CN-dppz. The first tap-based reduction of spin-doublet [Ru(tap)_2_(11,12-CN-dppz)]^+^ to spin-triplet [Ru(tap)_2_(11,12-CN-dppz)] at R2 decreased ν(C≡N) by merely −2 cm^–1^, while the intensity enhancement reached an overall factor of 8. Comparably, a red shift of ν(C≡N) by −27 cm^–1^ resulted from the 1e^–^ reduction of [Ru(tap)_2_(11-CN-dppz)]^2+^ at R1 (poorly resolved from R2), and the intensity enhancement was roughly 3-fold. Concomitant 1e^–^ reductions of the tap ligands (R2 and R3) caused only minor ν(C≡N) shifts of −3 cm^–1^ and increased the absorbance by overall factors of 6.5 and 8, respectively.

## Abstract 2

IR stretching vibrations of nitrile substituents at the pyrazine part of dipyrido[3,2-*a*:2′,3′-*c*]phenazine (dppz) are useful redox markers, sensitively reflecting electrochemical reduction of free and complexed dppz by the observed significant downshift of their wavenumber and strongly enhanced intensity. The presented complexes [Ru(tap)_2_(11-CN-dppz)]^2+^ and [Ru(tap)_2_(11,12-CN-dppz)]^2+^ (tap = 1,4,5,8-tetraazaphenanthrene) show relevance to photo-oxidation of DNA, and the detailed experimental and theoretical description of their reduction paths provides a solid basis for such electron-transfer studies.

## Introduction

Ruthenium(II) polypyridyl complexes continue to attract attention for their rich and controllable photophysical, photochemical, and electrochemical properties. Diverse types of tailored polypyridyl ligands bearing various substituents have extensively been studied for a range of applications, such as the modeling of photosystems, luminescent probes for deoxyribonucleic acid (DNA), ribonucleic acid or G-quadruplexes, etc.^1,2^

**Scheme 1:**
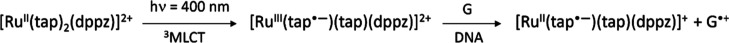
Photoreduction of [Ru(tap)_2_(dppz)]^2+^ by an Electron Transfer from the Guanine Base (G) in DNA^3^

Recently, spectroelectrochemical studies of the complex [Ru(tap)_2_(dppz)]^2+^ (tap = 1,4,5,8-tetraazaphenanthrene, dppz = dipyrido[3,2-*a*:2′,3′-*c*]phenazine) have revealed that the singly reduced cationic species can be generated via a photoelectron transfer from guanine (G) rather than a predicted proton-coupled electron transfer (Scheme 1). The infrared (IR) spectral changes accompanying the formation of the 1e^–^ reduced species were revealed by spectroelectrochemistry. The product, [Ru(tap)_2_(dppz)]^+^, shows near-IR electronic absorptions at 1970 and 2820 nm that have been assigned by time-dependent DFT (TDDFT) calculations to low-lying ligand-to-metal charge transfer transitions, viz. [tap]^•–^ → Ru.^3^

Withdrawing substituents at the phenazine distal ring of the intercalating dppz ligands significantly impact the electronic properties of the complex and affect its binding with DNA.^4^ Addition of phenyl groups at the dppz ligand (11-X-Ph-dppz; X = CN, *t*Bu) or phenyl–ethynyl groups terminated by the strongly electron-withdrawing nitrile group (11-CN-Ph-CC-dppz) or, on the contrary, the electron-donating *tert*-butyl group (11-*t*Bu-Ph-CC-dppz), extends the conjugation and lowers orbital energies of the dipyridyl and phenazine moieties.^5^ Consequently, the latter ligands exhibit less negative reduction potentials in comparison with unsubstituted dppz (−1.28 V vs SCE) and red-shifted electronic absorption features. The phenyl group induces a slightly stabilizing effect, with the reduction being localized on the phenazine part of dppz. Notably, the addition of the conjugated CN–phenyl–ethynyl group shifted the reduction much less negatively to −1.07 V vs SCE compared to 11-tBu-Ph-CC-dppz (−1.22 V vs SCE). However, the most pronounced effect is imposed by the nitrile substituent itself, with 11-CN-dppz reducing only at −0.91 V vs SCE.^5^

Further application of the CN functional groups regards their capacity to act as suitable IR-active tags^6−9^ when bound at dppz or other noninnocent conjugated α-diimine ligands. The change in the wavenumber of the ν(CN) stretching mode can be monitored for the parent and corresponding singly reduced radical anionic species. The collected spectroscopic data illustrate changes in the electronic distribution and intramolecular structure^9,10^ during (i) time-resolved infrared (TRIR) studies of charge-transfer excited states and photoinduced electron-transfer reactions^11^ and (ii) reference infrared spectroelectrochemical (IR SEC) measurements.^12^ A pioneering IR SEC study of a series of complexes [Ru(bpy)_3–*x*_(4,4′-CN-5,5′-Me-bpy)_*x*_]^2+^ (*x* = 1–3; bpy = 2,2′-bipyridine) focused on the responses of the 4,4′-CN-5,5′-Me-bpy ligands to their 1e^–^ reduction positioned less negatively compared to the unsubstituted bpy ligand(s). Notably, the first 1e^–^ reduction of each 4,4′-CN-5,5′-Me-bpy ligand at the Ru(II) center downshifts its ν_as_(CN) absorption by ca. 45 cm^–1^ and is accompanied by a large intensity enhancement (15-fold when comparing absorption maxima, or even 34-fold as based on the integrated band areas). In contrast, the successive reduction of the bpy ligand results only in a moderate ν_as_(CN) red shift and a minor intensity enhancement. The IR SEC data correspond with a similar, albeit less pronounced, ν_as_(CN) shift and intensity enhancement observed with TRIR spectroscopy for the ^3^MLCT excited state of [Ru^III^(bpy)_2_(4,4′-CN-5,5′-Me-bpy^•–^)]^2+^ from the IR SEC series.^13^

Generally, the enhancement of the ν(CN) molar absorptivity can be attributed to the coupling between the redistribution of charges and vibrational motion or mixing between a low-lying electronic transition and vibrational transition, the so-called vibronic coupling.^9−11,14^

**Chart 1:**
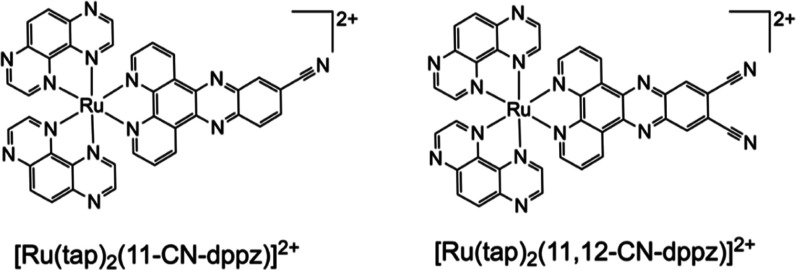
Schematic Molecular Structures of the Studied Complexes, with the PF_6_^–^ Counterions Omitted

This study extends the published pioneering IR SEC work^12^ on [Ru(bpy)_2_(4,4′-CN-5,5′-Me-bpy)]^2+^, focusing on the addition of the nitrile substituent(s) at the phenazine part of the dppz ligand in the ref (3) complex [Ru(tap)_2_(dppz)]^2+^ (Scheme 1). In general, it is aimed at proving the electron-transfer signaling concept^11−13^ of an IR-active tag, ν(CN), upon electrochemical reduction of heteroleptic Ru(II) α-diimine complexes. The investigated labeled ligands are 11-cyano-dipyrido[3,2-*a*:2′,3′-*c*]phenazine (11-CN-dppz) and 11,12-dicyano-dipyrido[3,2-*a*:2′,3′-*c*]phenazine (11,12-CN-dppz) (Chart 1). More practically, the selected pair represents heteroleptic Ru(II) complexes capable of binding to DNA as photo-oxidants operating via optical population of metal-to-ligand charge-transfer excited states. While the preceding investigations^12,13^ of [Ru(bpy)_2_(4,4′-CN-5,5′-Me-bpy)]^2+^ have proven the close similarity between the reduction of the 4,4′-CN-5,5′-Me-bpy ligand (i) mediated by an electrode and (ii) in a short-lived lowest ^3^MLCT excited state, the outcome is likely to be different for the tap and 11-CN-dppz/11,12-CN-dppz ligands. This expectation is justified by prior quantum-mechanical calculations carried out as part of this work. The detailed spectroelectrochemical study of the reduction paths of the complexes in Chart 1 therefore provides a valuable reference material for any future investigations of photodriven electron transfer directed to these ligands.

## Experimental Section

### General Procedures

Dichloromethane (DCM) and butyronitrile (PrCN) were freshly distilled from CaH_2_ prior to use under an inert atmosphere of dry argon. The supporting electrolyte, Bu_4_NPF_6_ tetrabutylammonium hexafluorophosphate (TBAH, Sigma-Aldrich), was recrystallized twice from absolute ethanol and dried under a vacuum at 373 K for 5 h and for an additional 20 min just prior to (spectro)electrochemical measurements. Standard Schlenk techniques under a dry argon gas atmosphere were applied to all procedures. ^1^H NMR spectra were recorded on a Bruker Nanobay spectrometer (400 MHz).

All ligands (tap, 11-CN-dppz, 11,12-CN-dppz) and the target complexes were synthesized according to the literature procedures.^15^ The purity of synthesized compounds was checked by ^1^H NMR (Figures S14–S17, Supporting Information).

### Methods

Cyclic voltammetry (CV) was performed under an inert argon atmosphere with a heart-shaped single-compartment glass cell containing a coiled Ag wire pseudoreference electrode, a 0.4 mm diameter Pt microdisc working electrode, and a coiled Pt wire counter electrode. The cell was held inside an earthed Faraday cage and connected to a Metrohm-Autolab PGSTAT302N potentiostat. The internal standard ferrocenium/ferrocene (Fc^+^/Fc) was added prior to the final scans. The sample solutions were 1 mM analyte dissolved in DCM or PrCN containing 10^–1^ M TBAH. Differential pulse voltammetry (DPV) was undertaken under the same conditions as CV.

Spectroelectrochemistry was performed using an optically transparent thin-layer electrochemical (OTTLE) cell^16^ equipped with a Pt minigrid working electrode. The sample solution contained 2 × 10^–1^ M TBAH as the supporting electrolyte and 2 mM redox active compounds in DCM. This concentration enabled parallel monitoring of the reduction steps with UV–vis and IR spectroscopies. The course of the spectroelectrochemical experiment was monitored by thin-layer CV (*v* = 2 mV s^–1^), with a potential control by a PalmSens EmStat3 potentiostat operated with PSTrace5 software.

IR spectroelectrochemistry was conducted using the Bruker Vertex 70v Fourier transform infrared (FT-IR) spectrometer equipped with a pyroelectric DTLaGS detector. UV–vis spectroelectrochemical measurements were performed using a Scinco S-3100 spectrophotometer with a diode array detector (200–1100 nm).

DFT calculations were performed in Gaussian 16, Revision C01 (G16),^17^ together with the three-parametrized Becke, Lee, Yang, and Park (B3LYP) functional.^18,19^ For the Ru atom, a quasi-relativistic effective-core pseudopotential and a corresponding optimized basis set^20^ was used, and for nonmetal atoms, the 6-311G(d) basis set.^21^ Solvent effects (DCM) were described by the conductor-like polarizable continuum model.^22^ Open shell systems were calculated using the unrestricted Kohn–Sham approach (UKS), and TDDFT was used to calculate electronic transitions and analyze them in terms of contributing one-electron excitations.^23^

## Results and Discussion

### CV and DFT Analysis of Frontier Molecular Orbitals

Free ligands 11-CN-dppz and 11,12-CN-dppz undergo two 1e^–^ electron reductions (Figures S1–S4, Supporting Information), but for 11-CN-dppz, the second reduction is hidden beyond the solvent/TBAH potential limit (Figures S1 and S2, Supporting Information). The reduction of [11,12-CN-dppz]^•–^ in DCM is quasi-reversible, probably due to limited solubility (Figure S4, Supporting Information). Moreover, the dianions become readily protonated.

The lowest unoccupied molecular orbital (LUMO) of 11-CN-dppz (Figure S18, Supporting Information) and 11,12-CN-dppz (Figure S25, Supporting Information) is predominantly localized on the phenazine part, and the same also applies to the distribution of the spin density in the corresponding radical anions. The involvement of the nitrile substituent(s) is limited, especially in [11,12-CN-dppz]^•–^ (Figure S25, Supporting Information). The successive 1e^–^ reduction to dianions is localized significantly more on the phenanthroline part, as revealed by the nature of the β-LUSO (Figures S24 and S31, Supporting Information). The added electrons combine to give diamagnetic dianions with high-lying LUMO on the pyridyl groups.

**Figure 1:**
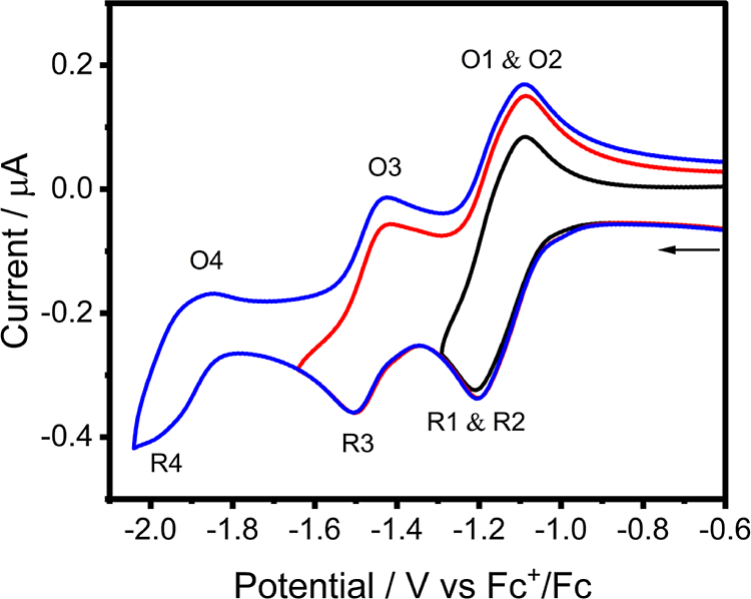
Cyclic voltammogram of [Ru(tap)_2_(11-CN-dppz)]^2+^ in DCM/TBAH showing the reductions to corresponding cationic (R1), neutral (R2), anionic (R3), and dianionic (R4) species. Experimental conditions: Pt microdisc working electrode, *T* = 298 K, and *v* = 100 mV s^–1^.

The complex [Ru(tap)_2_(11-CN-dppz)]^2+^ undergoes four observable 1e^–^ reductions, R1–R4 (Table 1), in both DCM (CV in Figure 1, and DPV in Figure S11, Supporting Information) and PrCN (CV in Figure S9, Supporting Information). The first two reversible 1e^–^ steps are better resolved in more polar PrCN. The close positions of the waves R1 and R2 seen in DCM (Figure 1) indicate that [Ru(tap)_2_(11-CN-dppz)]^2+^ becomes initially reduced at the remote phenazine moiety of 11-CN-dppz and the tap ligands, thereby limiting the effect of interelectronic repulsion on the wave separation. An accurate assignment of the reduction waves prior to spectroelectrochemical investigations has been facilitated by DFT calculations of frontier orbitals and the distribution of spin densities in the reduced species.

**Table 1:** Reduction Potentials (V vs Fc^+^/Fc) Determined for the Investigated CN-Substituted dppz Ligands, Their Ru–bis(tap) Complexes, and Reference [Ru(tap)_2_(dppz)]^2+^

compound	solvent	R1/O1(*E*_1/2_)	R2/O2(*E*_1/2_)	R3/O3(*E*_1/2_)	R4/O4(*E*_1/2_)	R5/O5(*E*_1/2_)
[Ru(tap)_2_(dppz)]^2+^^a^	MeCN	–1.18^a^^b^	–1.36^a^^b^	–1.45^b^^c^	–2.00^b^	d
11-CN-dppz	PrCN	–1.32	c			
	DCM	–1.44	c			
11,12-CN-dppz	PrCN	–1.14	–1.93			
	DCM	–1.17	–1.86			
[Ru(tap)_2_(11-CN-dppz)]^2+^	PrCN	–1.09	–1.23	–1.43	–1.85	c
	DCM	–1.16	–1.18	–1.46	–1.92	c
[Ru(tap)2(11,12-CN-dppz)]^2+^	PrCN	–0.87	–1.22	–1.44	–1.73	–2.11
	DCM	–0.82	–1.15	–1.42	–1.71	c

a Reference (3).

b Recalculated from ref (24) by using the factor of 0.382 (ref (25)).

c R3 localized on dppz.

d Not observed.

**Scheme 2:**
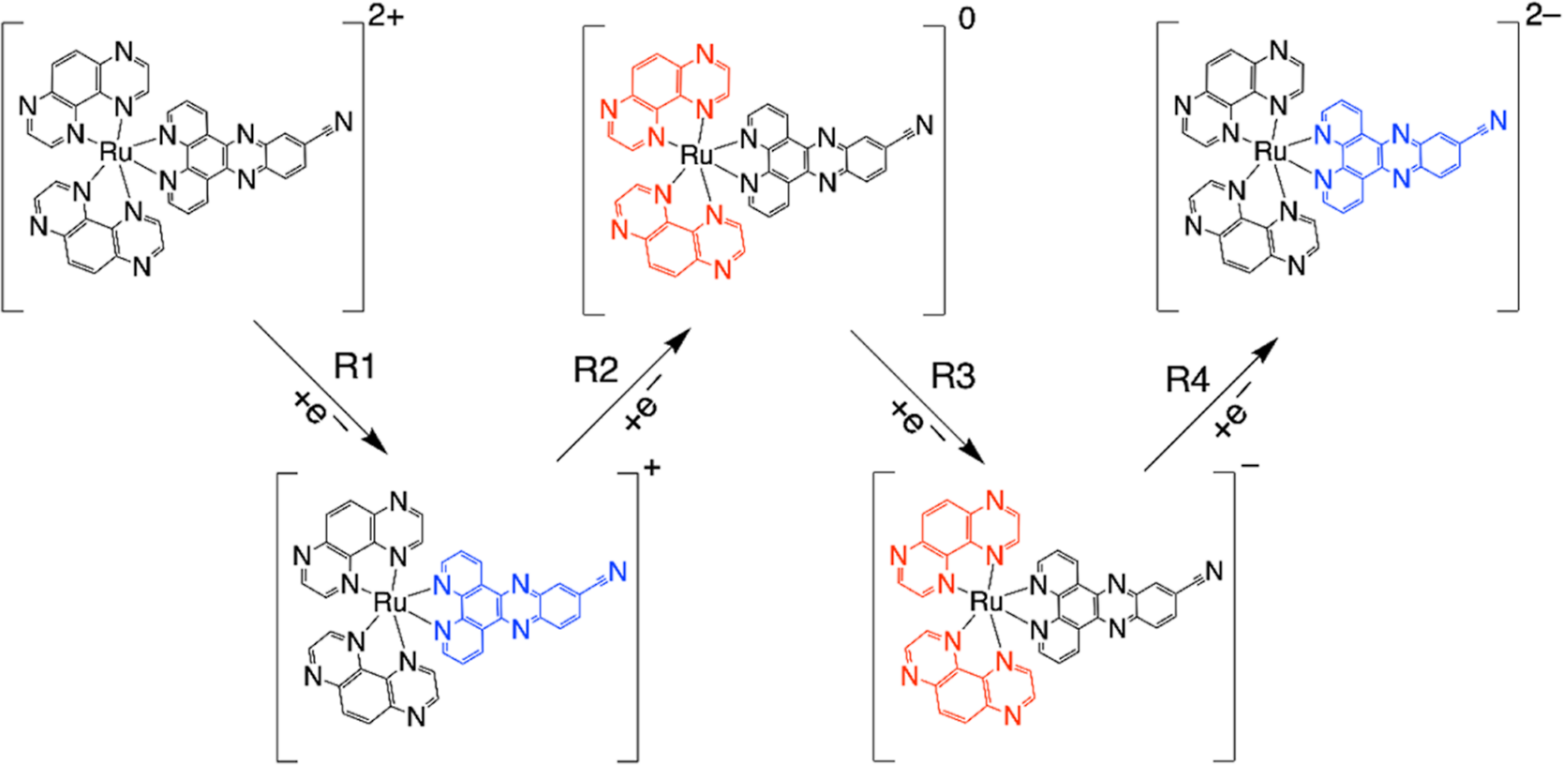
Reduction Pathway of [Ru(tap)_2_(11-CN-dppz)]^2+^; the Localization of Each 1e^–^ Reduction Step Is Highlighted; the Steps R2 and R3 Are Delocalized over Both Tap Ligands

The highest occupied molecular orbital (HOMO) and LUMO of the parent [Ru(tap)_2_(11-CN-dppz)]^2+^ are largely localized on the ruthenium(II) center and 11-CN-dppz, respectively (Figure S33, Supporting Information). Upon the initial 1e^–^ reduction of [Ru(tap)_2_(11-CN-dppz)]^2+^ to the monocation, the added spin density is exclusively localized on the 11-CN-dppz ligand [Figure S32 (left), Supporting Information]. The wave R1 can therefore be assigned to the reduction of 11-CN-dppz leading to a spin-doublet state. In [Ru(tap)_2_(11-CN-dppz)]^+^, α-HOSO and β-HOSO are located on the reduced ligand, [11-CN-dppz]^•–^, and α-LUSO and β-LUSO are located on both tap ligands. This outcome clearly reflects the large separation between the energy states of 11-CN-dppz and the tap ligands. The β-HOSO resides on the dppz part because of the destabilization of the π bonding orbital of 11-CN-dppz caused by placing 1e^–^ in the π* orbital of 11-CN-dppz. The following reduction step R2 is therefore delocalized over both tap ligands [Figure S32 (middle-left), Supporting Information], producing neutral [Ru(tap)_2_(11-CN-dppz)] in a spin-triplet state. Subsequent R3 belongs to the second reduction of the tap ligands generating [Ru(tap)_2_(11-CN-dppz)]^−^ in a spin-quadruplet state [Figure S32 (middle-right), Supporting Information]. R4 then represents the first wave in the second series of three reduction steps, converting the radical anionic ligands to dianions (Scheme 2). Notably, R4 is separated from R1 much like R2 is from R1 for the free 11,12-CN-dppz (Table 1), signaling the addition of the second electron at [11-CN-dppz]^•–^ to give [Ru(tap)_2_(11-CN-dppz)]^2–^ in a spin-triplet state with the 11-CN-dppz dianion [Figure S32 (right), Supporting Information].

**Figure 2:**
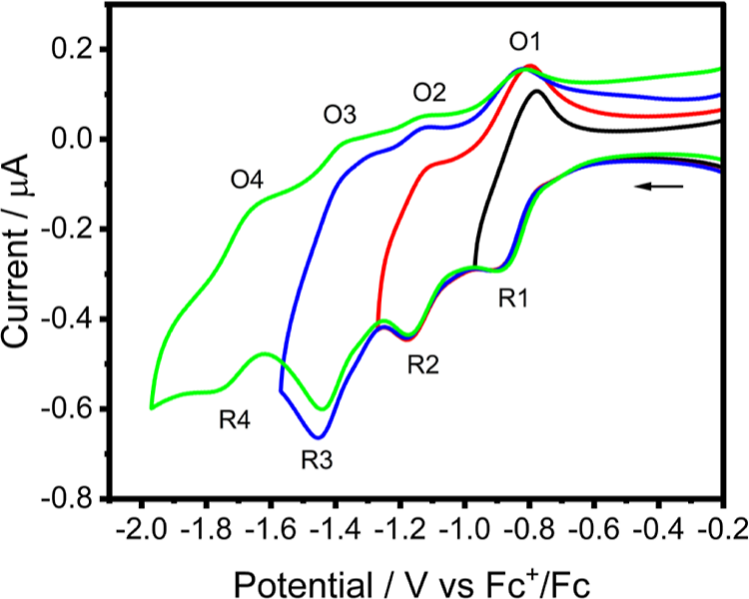
Cyclic voltammogram of [Ru(tap)_2_(11,12-CN-dppz)]^2+^ in DCM/TBAH showing the reduction steps to the corresponding monocationic (at R1), neutral (at R2), anionic (at R3), and dianionic (at R4) species. Experimental conditions: Pt microdisc working electrode, *T* = 298 K, *v* = 100 mV s^–1^.

Five reduction waves (R1–R5) are observed in the cyclic voltammogram of [Ru(tap)_2_(11,12-CN-dppz)]^2+^ in PrCN/TBAH (Table 1 and Figure S10, Supporting Information); the expected sixth wave remains unresolved at ambient temperature even when scanning with DPV (Figure S12, Supporting Information). In DCM/TBAH, only four reduction waves of [Ru(tap)_2_(11,12-CN-dppz)]^2+^ are seen in the limited negative potential window (Figure 2). In agreement with the different first reduction potentials of free 11-CN-dppz and 11,12-CN-dppz (Table 1), the reduction of chelated 11,12-CN-dppz at R1 occurs at a much less negative electrode potential compared to the CV of [Ru(tap)_2_(11-CN-dppz)]^2+^, where R1 and R2 are poorly resolved (see Figure 1). Consequently, the separation between R1 on 11,12-CN-dppz and R2 on the tap ligands has increased to 330 mV (DCM) and 350 mV (PrCN). The reduction potentials of the tap ligands (steps R2 and R3) remain unaffected by the addition of the second nitrile group at the distal ring. The same also applies to the separation of the two dppz-based reduction steps in [Ru(tap)_2_(11,12-CN-dppz)]^2+^ (R1 and R4) and free 11,12-CN-dppz (R1 and R2). Apparently, electronic communication between the nitrile-bearing dppz and tap ligands in the complexes is very limited. The only notable difference encountered in the cyclic voltammograms of [Ru(tap)_2_(11,12-CN-dppz)]^2+^ is the markedly irreversible shape of the R3/O3 couple in DCM/TBAH (Figure 2) and weak adsorption phenomena at R3 in PrCN/TBAH (Figure S10, Supporting Information). These features signal some unusual behavior that was investigated and explained by IR spectroelectrochemistry in the following section.

The frontier molecular orbitals of [Ru(tap)_2_(11,12-CN-dppz)]^2+^ (Figure S43, Supporting Information) have the same nature as those of [Ru(tap)_2_(11-CN-dppz)]^2+^(see above). The HOMO is largely localized on the ruthenium(II) center, whereas the LUMO resides exclusively on the 11,12-CN-dppz ligand and dominantly on its phenazine part because of a greater π-accepting capacity due to the two nitrile substituents. Upon the initial reduction to the corresponding monocationic complex at R1, the α-HOSO and β-HOSO are again localized on the 11,12-CN-dppz ligand and the α-LUSO and β-LUSO on the tap ligands. Likewise, the second reduction to the neutral complex at R2 occurs on the tap ligands, as illustrated by α-HOSO, whereas β-HOSO is localized again at the ruthenium center. The third reduction at R3 also resides on the tap ligands. A coupling between α-HOSO and β-HOSO situated on the tap ligands stabilizes these occupied orbitals.

**Scheme 3:**
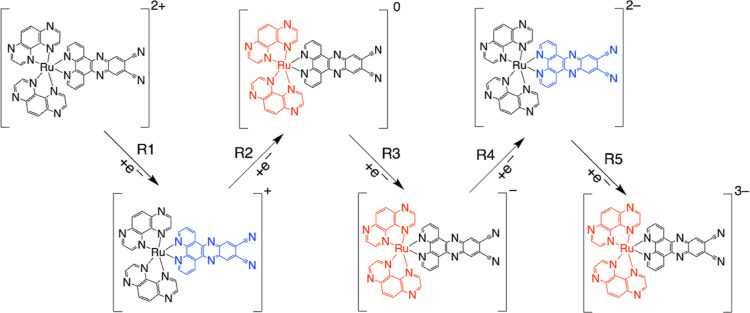
Reduction Pathway of [Ru(tap)_2_(11,12-CN-dppz)]^2+^; the Localization of Each 1e^–^ Reduction Step Is Highlighted; the Steps R2 and R3 Are Delocalized over Both Tap Ligands

Summarizing the CV data and the relevant DFT data given in the Supporting Information, the reduction path of [Ru(tap)_2_(11,12-CN-dppz)]^2+^ is presented in Scheme 3. The first reduction (R1) resides on 11,12-CN-dppz, producing the spin-doublet cationic complex [Figure S42 (left), Supporting Information]. It is followed by R2 and R3 populating different spin-orbitals at the tap ligands and producing spin-triplet neutral and spin-quadruplet anionic complexes [Figure S42 (middle-left and middle), Supporting Information], respectively. The fourth reduction (R4) lies on the [11,12-CN-dppz]^•–^ and gives the dianionic complex in a spin-triplet state [Figure S42 (middle-right), Supporting Information]. The fifth observable reduction (R5, in PrCN) is again tap-based and leads to the trianionic complex in a spin-quadruplet state [Figure S42 (right), Supporting Information]; the latter state is preferred by 0.36 eV when compared with the alternative spin-doublet state.

The nitrile groups at the terminal phenazine parts of the dppz ligands make them ideal candidates for IR spectroscopic tags, sensitively reflecting the nature of the multiple reduction steps described in this section. Both IR and UV–vis spectroelectrochemistry have been employed to monitor the reduction process, support the assignment of the steps along the reduction pathways, and contribute to a detailed description of the bonding and electronic absorption properties of the reduced species. The experimental spectroelectrochemical data have also been analyzed with the support from DFT and TDDFT calculations.

### IR Spectroelectrochemistry and DFT Harmonic Analysis

The IR ν(CN) region was monitored during all IR SEC experiments in DCM. The experimental and supporting DFT-calculated data are listed in Table 2.

**Table 2:** IR ν(CN) Wavenumbers (in cm^–1^) Measured and Calculated for 11-CN-dppz, 11,12-CN-dppz, [Ru(tap)_2_(11-CN-dppz)]^2+^, [Ru(tap)_2_(11,12-CN-dppz)]^2+^, and Their Reduced Species

compound	*x*	exp.	exp. Δν(CN)	calc^a^	calc. Δν(CN)
11-CN-dppz^*x*^	0	2232		2232	
	1–	2195	–37	2181	–51
11,12-CN-dppz^*x*^	0	2240		2239	
				2238	
	1–	2218	–22	2208	–31
		2204	–36	2199	–39
[Ru(tap)_2_(11-CN-dppz)]^*x*^	2+	2233		2238	
	1+	2206	–27	2200	–38
	0	2203	–3	2194	–6
	1–	2000	–3	2188	–6
[Ru(tap)_2_(11,12-CN-dppz)]^*x*^	2+	2240		2244	
				2243	
	1+	2222	–18	2218	–27
		2213	–27	2213	–31
	0	2221	–1	2215	–3
		2210	–3	2208	–5
	1–	2195^b^	*[−26]*	2212	–3
		2182^b^	*[−28]*	2204	–4

a Scaled by a factor of 0.956.

b The 3e^–^ reduced anion complex (*x* = 1−) is unstable at ambient temperature. The experimental wavenumbers in brackets represent the secondary species observed.

During the 1e^–^ reduction of free 11-CN-dppz and 11,12-CN-dppz at R1 (Table 1), the gradual decrease of the parent ν(CN) absorption is accompanied by the appearance of the new ν(CN) absorptions of the radical anionic products shifted to smaller wavenumbers and featuring ca. 4-fold enhancement in intensity (Figures S5 and S7, Supporting Information, respectively). Unlike the parent [11,12-CN-dppz] showing a single unresolved, although slightly asymmetric ν(CN) band at 2240 cm^–1^ (that is, 8 cm^–1^ higher than the singly CN-substituted phenazine moiety), [11,12-CN-dppz]^•–^ displayed better resolved ν_as_(CN) (2204 cm^–1^) and ν_s_(CN) (2218 cm^–1^) modes (Figure S7, Supporting Information). The IR SEC outcome for both ligands and their radical anions correlates with their DFT-calculated IR spectra (Figures S20 and S26, Supporting Information, respectively), as there is a significant decrease in the wavenumber [−Δν(CN)] and enhancement of the ν(CN) intensity of the singly reduced species. The quasi-degenerate asymmetric and symmetric ν(CN) modes of the disubstituted species are, however, less resolved in theory compared to the experimental spectrum for the DFT calculations conducted within the harmonic approximation (Table 2).

**Figure 3:**
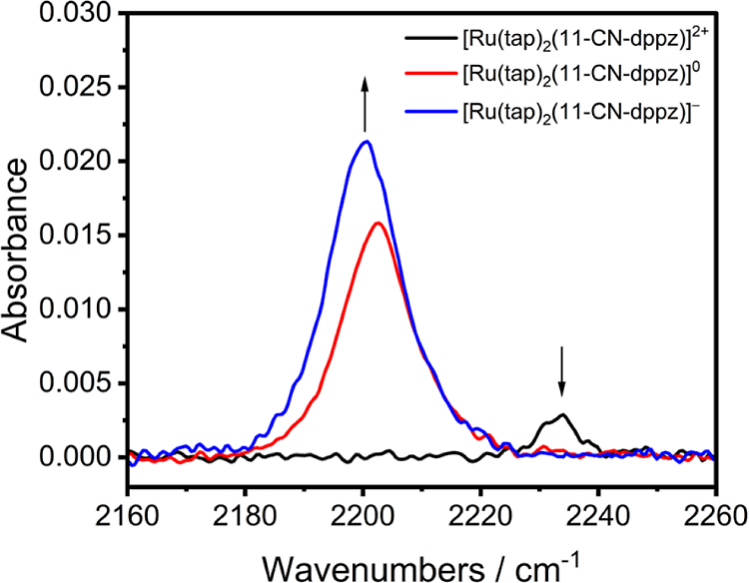
IR spectra [in the ν(CN) region] of [Ru(tap)_2_(11-CN-dppz)]^2+^ (black curve), 2e^–^ reduced [Ru(tap)_2_(11-CN-dppz)] (red curve; formed at poorly resolved R1 and R2), and 3e^–^ reduced [Ru(tap)_2_(11-CN-dppz)]^−^ (blue curve; formed at R3). Experimental conditions: Pt minigrid working electrode, OTTLE cell, DCM/TBAH, *T* = 298 K.

IR SEC monitoring of [Ru(tap)_2_(11-CN-dppz)]^2+^ (Figure 3) reveals its combined reduction to the neutral spin-triplet complex, resulting from the overlap of the 11-CN-dppz-based (R1) and tap-based (R2) reduction potentials (Figure 1). The singly reduced, spin-doublet monocation, [Ru(tap)_2_(11-CN-dppz)]^+^, can only be observed separately from 2e^–^ reduced [Ru(tap)_2_(11-CN-dppz)] from absorbance difference IR spectra at the initial stage (<20%) of the electrolysis at slightly resolved R1 and R2 (at the onset of the wave R1 in CV and TL-CV). Therefore, while it was possible to determine the −Δν(CN) value (becoming smaller compared with free 11-CN-dppz, Table 2), the enhancement of the ν(CN) intensity by a factor of 3 is only a rough estimate. The subsequent addition of the second and third unpaired electron to both tap ligands at R2 and R3, producing ultimately spin-quadruplet [Ru(tap)_2_(11-CN-dppz)]^−^, led for both steps to a small −Δν(CN) shift of 3 cm^–1^ and, surprisingly, to a significant enhancement of the ν(CN) intensity by an overall factor of 6.5 at R2 and 8 at R3. The DFT-calculated IR spectra for the separate steps R1–R3 are shown in Figure S34 (Supporting Information). While the relative −Δν(CN) shifts are reproduced appreciably, the main gain to the ν(CN) intensity is obtained in theory only from R1; on the contrary, passing R2 results in a significant intensity drop that is partly compensated in the following step R3 to give [Ru(tap)_2_(11-CN-dppz)]^−^ (in line with the corresponding experimental observation).

**Figure 4:**
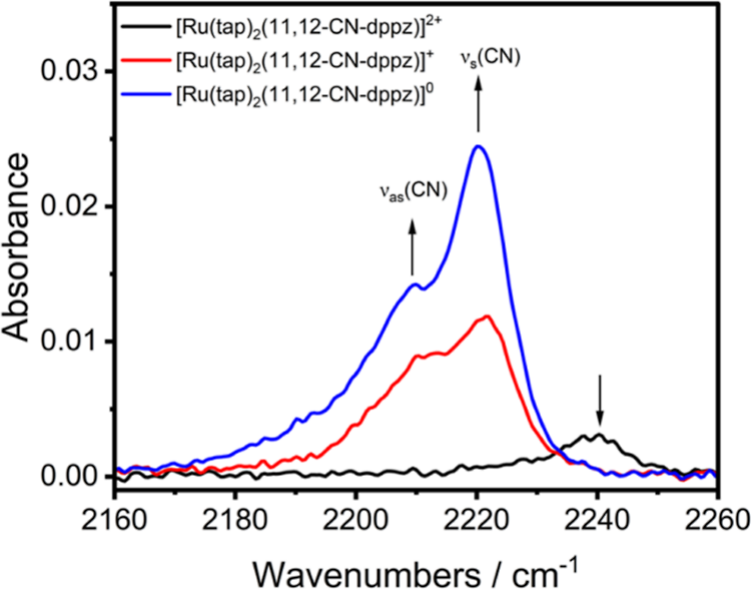
IR spectra [in the ν(CN) region] of [Ru(tap)_2_(11,12-CN-dppz)]^2+^ (black curve), 1e^–^ reduced [Ru(tap)_2_(11,12-CN-dppz)]^+^ (red curve; formed at R1), and 2e^–^ reduced [Ru(tap)_2_(11,12-CN-dppz)] (blue curve; formed at R2). Experimental conditions: Pt minigrid working electrode, an OTTLE cell, DCM/TBAH, *T* = 298 K.

The IR SEC monitoring of the reduction path of [Ru(tap)_2_(11,12-CN-dppz)]^2+^ has confirmed the distinct 1e^–^ reduction steps R1 and R2 (Table 1) being localized on the 11,12-CN-dppz and tap ligands, respectively (Figure 4). The corresponding spin-doublet cationic and spin-triplet neutral products exhibit resolved ν_s_(CN) and ν_as_(CN) modes (Table 2), much like free [11,12-CN-dppz]^•–^ (Figure S7, Supporting Information). The first reduction to [Ru(tap)_2_(11,12-CN-dppz)]^+^ (R1 in Figure 2) decreased the ν(CN) wavenumbers significantly, albeit less than observed for free 11,12-CN-dppz. The intensity of the new asymmetric ν(CN) absorption band increased by a factor of 4. This behavior is consistent with reduction of the 11,12-CN-dppz ligand. The second reduction step (R2 in Figure 2) to [Ru(tap)_2_(11,12-CN-dppz)] encompasses both tap ligands and, in accordance with this assignment, the ν(CN) wavenumbers become reduced by merely 2 cm^–1^. Surprisingly, with reference to the parent dication, the ν(CN) intensity grew further at R2 by an overall factor of 8 (Figure 4) despite the remote position of the nitrile substituents from the tap redox centers active at R2. This experimental outcome also deviates from the small intensity increase at R2 predicted by DFT calculations (Figure S44, Supporting Information). Nevertheless, the stability and correct assignment of [Ru(tap)_2_(11,12-CN-dppz)] have been confirmed by the reoxidation step at the O2 (Figure 2) that fully recovered the precursor cationic complex.

In contrast with the reversible reduction of neutral [Ru(tap)_2_(11-CN-dppz)] to the corresponding anion (Figure 3), the third 1e^–^ step along the reduction path of [Ru(tap)_2_(11,12-CN-dppz)]^2+^ did not yield stable [Ru(tap)_2_(11,12-CN-dppz)]^−^ with the ν(CN) wavenumbers predicted to be only slightly smaller than 2221 and 2210 cm^–1^ determined for the neutral precursor (Table 2). Instead, the product generated at R3 exhibits an asymmetric band at 2195 cm^–1^ (Figure S13, Supporting Information). Such a large red shift of ν(CN) evokes reduction of the [11,12-CN-dppz]^•–^ ligand taking place already at R3 instead of R4 (Table 1 and Scheme 3; see also Figure S44, Supporting Information). Importantly, the reoxidation did not recover [Ru(tap)_2_(11,12-CN-dppz)] (Table 2) but gave a slowly decaying new species absorbing at 2205 cm^–1^. The irreversible reduction of [Ru(tap)_2_(11,12-CN-dppz)] at R3, which is already indicated by the atypical wave shape in the conventional cyclic voltammogram (Figure 2), was not explored in greater detail to elucidate the unusual behavior.

It is noteworthy that the intimate IR spectroscopic response of the terminal nitrile groups in [Ru(tap)_2_(11,12-CN-dppz)]^2+^ to the separate 1e^–^ and 2e^–^ reduction steps reveals notable differences from that reported^12^ for the related heteroleptic complex [Ru(bpy)_2_(4,4′-CN-5,5′-Me-bpy)]^2+^. The common point is the lifted degeneracy of ν_s_(CN) (A_1_ symmetry) and ν_as_(CN) (B_1_ symmetry) modes attributed to the occupation of the π* LUMO in the parent dicationic complexes upon the initial 1e^–^ reduction of the 11,12-CN-dppz and 4,4′-CN-5,5′-Me-bpy ligands, which is delocalized in both cases also over the nitrile substituents at the conjugated aromatic rings (see Figure S42 in the Supporting Information). The localization of some spin density at the terminal N atoms of the C≡N groups in the singly reduced state results in an increased dipole moment derivative along the coordinates of the ν_s_(CN) and ν_as_(CN) vibrations, resulting in their amplified intensities. At the same time, the antibonding nature of the LUMO also with respect to the C≡N bonds explains the downshift of the ν_s_(CN) and ν_as_(CN) IR absorption bands upon electrochemical reduction. Its magnitude depends on the degree of involvement of the nitrile groups in the LUMO. Compared to [Ru(bpy)_2_(4,4′-CN-5,5′-Me-bpy^•–^)]^+^, singly reduced [Ru(tap)_2_(11,12-CN-dppz^•–^)]^+^ exhibits a smaller downshift (−18/–27 vs −29/–46 cm^–1^) and splitting (9 vs 17 cm^–1^) of the ν_s_(CN)/ν_as_(CN) bands and also a ca. 4 times smaller intensity enhancement. These differences correspond with a stronger electron-acceptor character of 11,12-CN-dppz compared to 4,4′-CN-5,5′-Me-bpy, as reflected in the first reduction potentials (R1) of the complexed ligands (vs Fc^+^/Fc): −1.09 V (11,12-CN-dppz) vs −1.38 V (4,4′-CN-5,5′-Me-bpy) in PrCN/MeCN. Apparently, the nitrile substituents at C11 and C12 of the distal phenazine ring and their IR stretching modes become less affected by the electrochemical reduction than those at C4 and C4′ of the pyridyl rings.

A more striking difference between the IR spectra of singly reduced [Ru(tap)_2_(11,12-CN-dppz^•–^)]^+^ and [Ru(bpy)_2_(4,4′-CN-5,5′-Me-bpy^•–^)]^+^ is the unexpected larger intensity enhancement observed for the ν_s_(CN) (A_1_) mode of [11,12-CN-dppz]^•–^, whereas [4,4′-CN-5,5′-Me-bpy]^•–^ features the opposite trend with the dominantly enhanced ν_as_(CN) (B_1_) mode. It is anticipated that the push–pull nature of the asymmetric stretching mode causes a larger change in the local dipole moment and consequently larger B_1_ intensity enhancement, as reported^12^ for [Ru(bpy)_2_(4,4′-CN-5,5′-Me-bpy^•–^)]^+^. On the other hand, the higher A_1_ than B_1_ intensity is likely determined by the change of the dipole moment of the whole [11,12-CN-dppz]^•–^ ligand/complex.

The tap-based second and third 1e^–^ reduction of [Ru(tap)_2_(11,12-CN-dppz)]^+^ to [Ru(tap)_2_(11,12-CN-dppz)] and [Ru(tap)_2_(11,12-CN-dppz)]^−^ has been predicted by DFT calculations to result only in a little downward shift of the ν_s_(CN) (A_1_) and ν_as_(CN) (B_1_) modes by a few wavenumbers and a minor intensity enhancement (see Figure S44, Supporting Information). The following marked downward shift and major intensity enhancement have been predicted for (unstable) [Ru(tap)_2_(11,12-CN-dppz)]^2–^ bearing the fully reduced [11,12-CN-dppz]^2–^ ligand. This anticipated behavior has indeed been reported^12^ for the series [Ru(bpy)_2_(4,4′-CN-5,5′-Me-bpy)]^*n*^ (*n* = 2+, 1+, 0, 1−). Surprisingly, IR SEC experiments (Figure 4) have revealed that the molar absorptivity of the ν_s_(CN) and ν_as_(CN) bands continues to rise in the same pace even for spin-triplet [Ru(tap)_2_(11,12-CN-dppz)] with the spin density delocalized over all three chelating ligands (Figure S42, Supporting Information), differently from reference [Ru(bpy)(bpy^•–^)(4,4′-CN-5,5′-Me-bpy^•–^)]. A plausible explanation of the additional A_1_ intensity enhancement may consider an alternative mechanism such as vibronic interactions with low-lying electronic transitions.^26^

### UV–Vis Spectroelectrochemistry and TDDFT Calculations

#### Ligands

The reference experimental UV–vis spectra recorded before and after the 1e^–^ reduction of free 11-CN-dppz and 11,12-CN-dppz to the corresponding radical anions are presented in the Supporting Information as Figures S6 and S8, respectively. Their analyses and accurate assignments of electronic transitions contributing to the absorption bands are facilitated by TDDFT calculations. The simulated UV–vis absorption spectra of 11-CN-dppz/[11-CN-dppz]^•–^ and 11,12-CN-dppz/[11,12-CN-dppz]^•–^ are depicted in Figures S21 and S27 (Supporting Information), respectively. The calculated individual vertical electronic excitations in 11-CN-dppz/[11-CN-dppz]^•–^ and 11,12-CN-dppz/[11,12-CN-dppz]^•–^ are visualized in Figures S21, S23 and S28, S30 (Supporting Information) and assigned in Tables S1–S4 (Supporting Information), respectively. From the diagnostic point of view, both radical anions feature characteristic π* → π* intraligand absorptions in the visible spectral range, with maxima at 620 nm (mainly α-HOSO → α-LUSO + 2) for [11-CN-dppz]^•–^ and at 589 nm (mainly α-HOSO → α-LUSO + 3) for [11,12-CN-dppz]^•–^. The visible electronic absorption of [tap]^•–^ in singly reduced [Ru(tap)_2_(dppz)]^+^ has been taken from the literature.^3^

#### Parent Dicationic Complexes

**Figure 5:**
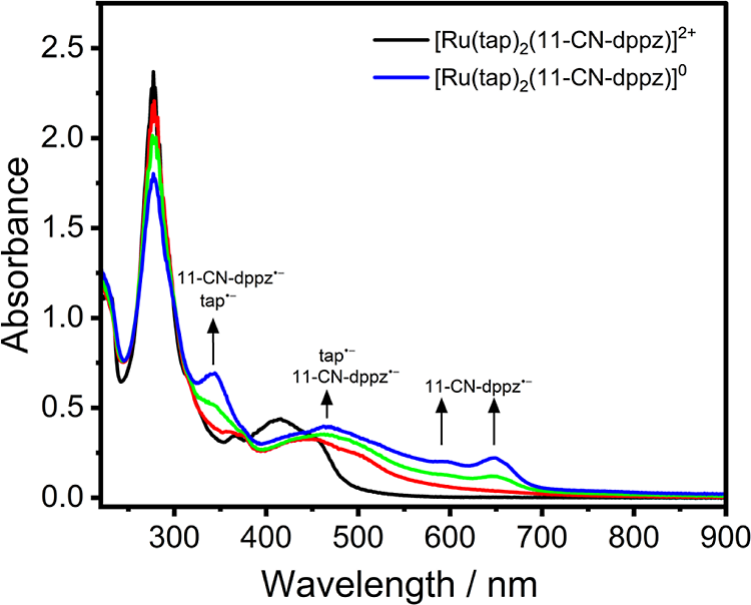
UV–vis SEC spectra of [Ru(tap)_2_(11-CN-dppz)]^2+^ (black curve) and 2e^–^ reduced [Ru(tap)_2_(11-CN-dppz)] (blue curve; formed at poorly resolved R1 and R2). Experimental conditions: Pt minigrid working electrode, an OTTLE cell, DCM/TBAH, *T* = 298 K.

**Figure 6:**
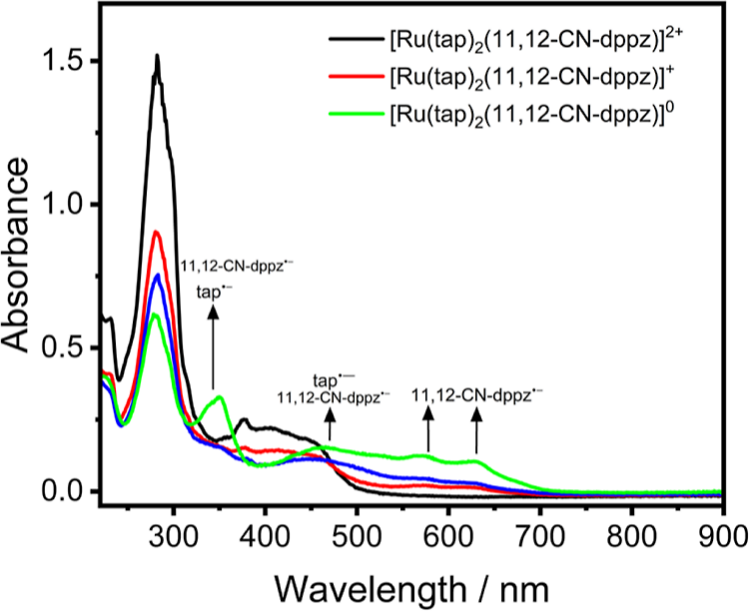
UV–vis SEC spectra of [Ru(tap)_2_(11,12-CN-dppz)]^2+^ (black curve), 1e^–^ reduced [Ru(tap)_2_(11,12-CN-dppz)]^+^ (red curve; formed at R1) and 2e^–^ reduced [Ru(tap)_2_(11,12-CN-dppz)] (green curve; formed at R2). The blue curve corresponds to the transition between the 1e^–^ reduced and 2e^–^ reduced complexes. Experimental conditions: Pt minigrid, an OTTLE cell, DCM/TBAH, and *T* = 298 K.

The UV–vis spectrum of [Ru(tap)_2_(11-CN-dppz)]^2+^ (Figure 5) is dominated by a broad band at about 414 nm, with a shoulder at about 450 nm. Based on TDDFT calculations (Figure S36, Table S5, Supporting Information), the band at 414 nm is assigned to a combined MLCT transition consisting of *d*_*y*_(Ru) → π*(11-CN-dppz), *d*_*z*_(Ru) → π*(tap) and *d*_*x*_(Ru)/*d*_*y*_(Ru)/*d*_*z*_(Ru) → π*(tap). The shoulder corresponds to the *d*_*z*_(Ru) → π*(11-CN-dppz) and *d*_*x*_(Ru)/*d*_*y*_(Ru)/*d*_*z*_(Ru) → π*(tap) transitions. Similarly, the UV–vis spectrum of [Ru(tap)_2_(11,12-CN-dppz)]^2+^ (Figure 6) contains a broad band at 404 nm and a shoulder at 457 nm. Based on TDDFT calculations (Figure S46, Table S8, Supporting Information), the broad band at 404 nm is assigned to an MLCT transition consisting of *d*_*x*_(Ru)/*d*_*y*_(Ru)/*d*_*z*_(Ru) → π*(tap). The shoulder mainly corresponds to optical excitation *d*_*x*_(Ru)/*d*_*y*_(Ru)/*d*_*z*_(Ru) → π*(tap), with a contribution from *d*_*x*_(Ru)/*d*_*y*_(Ru) → π*(11,12-CN-dppz).

#### One-Electron-Reduced Monocationic Complexes

Monitoring the reduction of parent complex [Ru(tap)_2_(11-CN-dppz)]^2+^ (Figure 5), the spectral changes in the visible region correspond to the poorly resolved one-electron reductions of both the 11-CN-dppz and tap ligands at R1/R2. Based on the reference [Ru(tap)_2_(dppz)]^2+^ complex, the [tap]^•–^ ligand is known to absorb in the region of 400–500 nm.^3^ Therefore, using the reference absorption of [11-CN-dppz]^•–^ (Figure S6, Supporting Information), [tap]^•–^ can be identified by the appearance of the absorption bands at 344 and 466 nm in 2e^–^ reduced [Ru(tap)_2_(11-CN-dppz)]. However, the electronic transitions in 1e^–^ reduced [Ru(tap)_2_(11-CN-dppz)]^+^ (the red curve in Figure 5) were also calculated by TDDFT (Figure S38, Table S6, Supporting Information). The band at 455 nm is dominated by MLCT [Ru → tap; α-HOSO – 3 → α-LUSO + 2 (44%)] with a small contribution from MLCT (Ru → [11-CN-dppz]^•–^; β-HOSO – 4 → β-LUSO) and LLCT ([11-CN-dppz]^•–^ →tap; α-HOSO – 1 → α-LUSO). The shoulder at about 502 nm is assigned to ILCT ([11-CN-dppz]^•–^-based; α-HOSO → α-LUSO + 11) and LLCT ([11-CN-dppz]^•–^ → tap; α-HOSO → α-LUSO + 8, α-LUSO + 9). The spectral tailing at 600 nm is dominated by ILCT ([11-CN-dppz]^•–^-based; α-HOSO → α-LUSO + 10, α-LUSO + 11) with the contribution of LLCT ([11-CN-dppz]^•–^ → tap; α-HOSO → α-LUSO + 8, α-LUSO + 9).

The UV–vis spectra of [Ru(tap)_2_(11,12-CN-dppz)]^2+/+/0^ (Figure 6) support the CV- and IR-based assignment of the first reduction (at R1) localized on the 11,12-CN-dppz ligand due to the rising absorption band at 550–700 nm that resembles free [11-CN-dppz]^•–^ at 597 nm (Figure S8, Supporting Information). Upon the second reduction delocalized over both tap ligands, the absorbance in this region does not change significantly. Instead, there is rising absorption of [tap]^•–^ at 450–550 nm, in agreement with the [Ru(tap)_2_(dppz)]^+^ complex.^3^ From the DFT-calculated UV–vis spectra (Figure S45, Supporting Information), this correlates with the experimental spectra as there is rising absorption of [11,12-CN-dppz]^•–^ at 550 nm, tailing into the higher energy MLCT transitions, which cannot be observed in the free 11,12-CN-dppz ligand. For 1e^–^ reduced [Ru(tap)_2_(11,12-CN-dppz)]^+^ (Figure S48, Table S9, Supporting Information), the transitions at 630 and 580 nm are assigned to the ILCT ([11,12-CN-dppz]^•–^-based; α-HOSO → α-LUSO + 8), LLCT ([11,12-CN-dppz]^•–^ → tap: α-HOSO → α-LUSO + 6, α-LUSO + 7) and combined LLCT/ILCT (α-HOSO → α-LUSO + 10, α-LUSO + 11). The band at 460 nm is dominated by MLCT (Ru → tap): α-HOSO – 2 → α-LUSO + 2 (56%), with a contribution of LLCT ([11,12-CN-dppz]^•–^ → tap, β-HOSO → β-LUSO + 3). The remaining transitions of [Ru(tap)_2_(11,12-CN-dppz)]^+^ are assigned in Table S9 (Supporting Information), including the lowest-energy near-infrared absorption tailing to the SWIR region, which was observed during the IR SEC measurements.

#### Two-Electron-Reduced Neutral Complexes

Turning our attention to 2e^–^ reduced [Ru(tap)_2_(11-CN-dppz)] (Figure S40 and Table S7, Supporting Information), its UV–vis absorption spectrum resembles that of 1e^–^ reduced [Ru(tap)_2_(11-CN-dppz)]^+^ (Figure 5), but the origin of transitions is different. The absorption bands at 647 and 591 nm mainly correspond to the ILCT transition ([11-CN-dppz]^•–^-based; α-HOSO → α-LUSO + 7), with a contribution of LLCT ([11-CN-dppz]^•–^ → [tap]^•–^ α-HOSO → α-LUSO + 5, LUSO + 6). The electronic transition at 467 nm is dominated by ILCT ([11-CN-dppz]^•–^-based; α-HOSO → α-LUSO + 10) with the contribution of LLCT ([11-CN-dppz]^•–^ → [tap]^•–^; α-HOSO → α-LUSO + 6) and ILCT ([tap]^•–^-based; α-HOSO – 1 → α-LUSO + 9). The absorption band at 344 nm is mainly assigned to MLCT2 transitions (Ru → [11-CN-dppz]^•–^; α-HOSO – 3 → α-LUSO + 3) and MLCT1 (Ru → [tap]^•–^; β-HOSO – 3 → β-LUSO + 3; β-HOSO – 2 → β-LUSO + 4).

Similarly, 2e^–^ reduced [Ru(tap)_2_(11,12-CN-dppz)] shows a transition at 630 nm, which is assigned to ILCT ([11,12-CN-dppz]^•–^-based: α-HOSO – 1 → α-LUSO + 5). The band at about 580 nm mainly corresponds to MLCT1 (Ru → [tap]^•–^) and MLCT2 (Ru → [11,12-CN-dppz]^•–^), with contributions from ILCT ([11,12-CN-dppz]^•–^-based: α-HOSO – 1 → α-LUSO + 8) and LLCT ([11,12-CN-dppz]^•–^ → [tap]^•–^: α-HOSO – 1 → α-LUSO + 6). The absorption at 460 nm is dominated by MLCT1 (Ru → [tap]^•–^: β-HOSO → β-LUSO + 3; β-HOSO – 1 → β-LUSO + 2, β-LUSO + 4), with contributions from MLCT2 (Ru → [11,12-CN-dppz]^•–^: β-HOSO – 3→ β-LUSO + 3; β-HOSO – 1 → β-LUSO + 1) and LLCT ([11,12-CN-dppz]^•–^ → [tap]^•–^: β-HOSO – 2 → β-LUSO + 2). The remaining transitions in [Ru(tap)_2_(11,12-CN-dppz)] are assigned in Table S10 (Supporting Information).

### Charge-Transfer Excited-State Properties Determined by DFT Calculations

The TDDFT-calculated data for electronic transitions allow prediction of the nature of the low-lying excited states of the studied complexes for upcoming studies by ultrafast laser spectroscopies. In the case of [Ru(tap)_2_(11-CN-dppz)]^2+^, the excitation at 400 nm mainly leads to optical population of an ^1^MLCT excited state involving π*(tap) orbitals (LUMO + 1, LUMO + 2, LUMO + 3, LUMO + 4), with contributions from an MLCT excitation into the dppz part (LUMO, LUMO + 5). These vertical excitations (Table S5, Supporting Information) are followed by ultrafast intersystem crossing, a well-known behavior of [Ru(diimine)_3_]^2+^ complexes.^3,12,13,27−29^ However, the lowest ^3^MLCT excited state becomes localized exclusively on the tap ligand in the trans position to the nitrile group/substituent on dppz (Figure S50, Supporting Information).

The 400 nm excitation of [Ru(tap)_2_(11,12-CN-dppz)]^2+^ implies an exclusive transition of spin density into the π*(tap) orbitals, reaching the same lowest ^3^MLCT state (Figure S51, Supporting Information) as predicted for [Ru(tap)_2_(11-CN-dppz)]^2+^. On the contrary, the contribution of the dppz-directed MLCT states will be very small (Table S8, Supporting Information).

The lowest Ru → tap ^3^MLCT excited state is reached by optical excitation of both [Ru(tap)_2_(NN)]^2+^ complexes, despite their LUMO being localized on the 11-CN-dppz and 11,12-CN-dppz ligands, as described in the preceding spectroelectrochemistry sections. A detailed study of the excited-state properties of these complexes combined with the spectroelectrochemical data sets may unravel the mechanism of photoinduced oxidation of DNA by electron transfer from guanine bases into the intercalated Ru–dppz moiety, most likely triggered by the population of the low-lying [Ru^III^(tap^•–^)(tap)(11-CN-dppz/11,12-CN-dppz)]^2+^^3^MLCT excited states.

## Conclusions

The studied complexes [Ru(tap)_2_(NN)]^2+^ (NN = 11-CN-dppz and 11,12-CN-dppz) were shown to undergo 1e^–^ reduction (R1) localized on the NN ligand. The subsequent two 1e^–^ reduction steps of the spin-doublet cationic complexes (R2 and R3) involve both tap ligands, producing the spin-triplet neutral complexes and spin-quadruplet anionic complexes. For NN = 11-CN-dppz the R1 and R2 steps overlap. The localization of the first three reduction steps (in DCM) inferred from the cyclic voltammetric responses has been confirmed spectroelectrochemically by monitoring the IR-active ν(C≡N) vibrations and appearance of new UV–vis absorption features assigned to intraligand π* → π* [NN]^•–^ and π* → π* [tap]^•–^ transitions along with MLCT (Ru → tap/NN and Ru → [tap/NN]^•–^) as well as LLCT ([NN]^•–^ → tap and [NN]^•–^ → [tap]^•–^) transitions elucidated by TDDFT calculations. The downshifts of the ν(C≡N) wavenumbers sensitively reflect the added electron density on the phenazine part of the NN ligands (large values) or on the remote tap ligands (small values). The intensity enhancement of the IR-active ν(C≡N) mode is large at both NN-based R1 and tap-based R2, which contradicts the theoretical predictions of a small ν(C≡N) intensity change at R2 and also differs from the more redox site-selective ν(C≡N) enhancement factors described in the literature for the related series of complexes [Ru(bpy)_3–*x*_(4,4′-CN-5,5′-Me-bpy)_*x*_]^2+^ (*x* = 1–3).^12^ This study further reveals that the ν(C≡N) vibrations of nitrile substituents on redox-active ligands can be utilized as convenient IR probes in electron-transfer systems despite the initially low IR intensity and Fermi resonances.^30^ It is anticipated that the strong increase in molar absorptivity observed during a reduction step or optical excitation localized in their vicinity facilitates the detection of the IR ν(C≡N) absorption at low concentrations, for example, during photoexcitation of Ru–polypyridyl-based probes intercalated in DNA^31^ in kinetic studies carried out by time-resolved IR laser spectroscopy. Combined with DFT and TDDFT calculations, the results indicate that both title complexes are promising IR-active tags for monitoring photo-oxidation of DNA by electron transfer from the guanine base^3^ to the intercalated complex in its lowest ^3^MLCT excited state residing on a single Ru^III^–tap^•–^ moiety, despite the NN-based π* LUMO of the parent dicationic complexes.

## Supporting Information Available

CV, UV–vis SEC and IR SEC of the free NN ligands in [Ru(tap)_2_(NN)]^2+^, CV (in PrCN) and DPV of the complexes, and ^1^H NMR spectra and complete DFT and TDDFT calculations of all the studied compounds (PDF)
