# Supplementary material for: Nitrile Substituents at the Conjugated Dipyridophenazine Moiety as Infrared Redox Markers in Electrochemically Reduced Heteroleptic Ru(II) Polypyridyl Complexes
Source: Inorg Chem. 2024 Jan 23;63(5):2460–9. doi: 10.1021/acs.inorgchem.3c03484 (PMC10848246; doi:10.1021/acs.inorgchem.3c03484)
Supplement: Supplementary file 1 — ic3c03484_si_001.pdf [file ic3c03484_si_001.pdf]

Supplementary information

Nitrile Substituents at the Conjugated Dipyridophenazine Moiety as Infrared Redox Markers in Electrochemically Reduced Heteroleptic Ru(II) Polypyridyl Complexes

Elizabeth Sumner[a], Martin Pižl[a,b], Kane T. McQuaid[a], František Hartl[a]**

[a] Department of Chemistry, University of Reading, Whiteknights, Reading RG6 6DX, United Kingdom
[b] Department of Inorganic Chemistry, University of Chemistry and Technology Prague, Technická 5, 166 28 Prague 6, Czech Republic

Corresponding Authors′ e-mail addresses: martin.pizl@vscht.cz; f.hartl@reading.ac.uk.

Table of Contents

1. ***Cyclic voltammetry of free ligands*** S-2
2. ***Spectroelectrochemistry of free ligands*** S-5
3. ***Cyclic voltammetry of studied complexes in PrCN*** S-8
4. ***Differential pulse voltammetry of studied complexes*** S-9
5. ***Spectroelectrochemistry of studied complexes*** S-10
6. ***NMR spectra of synthesized compounds*** S-11
7. ***DFT and TDDFT calculations on 11-CN-dppz and 11,12-CN-dppz*** S-15
8. ***DFT and TDDFT calculations on studied complexes*** S-27
9. ***DFT-calculated charge-transfer excited-state properties of studied complexes*** S-52

1. Cyclic voltammetry of free ligands

Free 11-CN-dppz undergoes reversible 1e^{-} reduction in both PrCN ($E_{1/2} = -1.32$ V) and DCM ($E_{1/2} = -1.44$ V) (Scheme S1 top). However, in PrCN (Figure S1), upon the addition of the Fc^{+}/Fc standard, the reduction becomes irreversible. In both solvents, the second reduction of the radical anion to dianion was too negative to be observed.

For free 11,12-CN-dppz, both 1e^{-} reduction steps (Scheme S1 bottom) are observed in PrCN (Figure S3) and DCM (Figure S4). In PrCN, the first reduction ($E_{1/2} = -1.14$ V) and second reduction ($E_{1/2} = -1.93$ V) waves are both reversible. In DCM, only the first reduction ($E_{1/2} = -1.17$ V) wave is reversible. The second reduction ($E_{1/2} = -1.86$ V) wave is quasi-reversible, potentially due to some reactivity occurring on the CV timescale. In comparison with free 11-CN-dppz, the reduction potential is less negative because the second nitrile substituent stabilizes the LUMO.

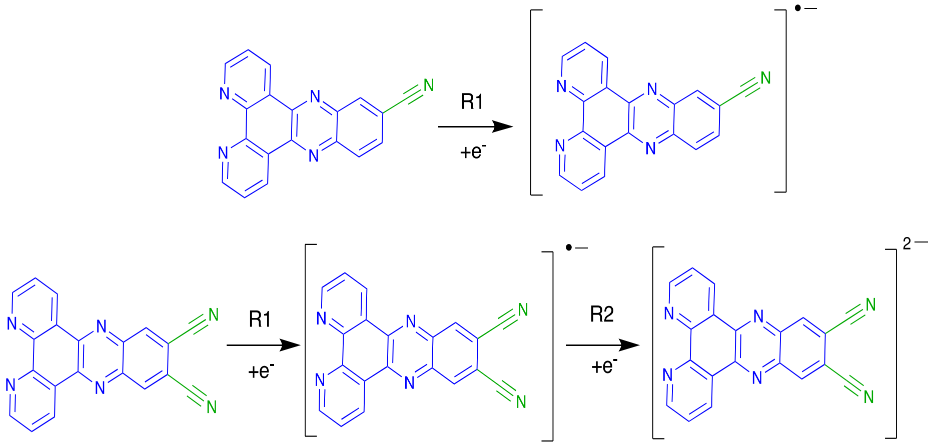

Scheme S1. Reduction pathways of 11-CN-dppz (top) and 11,12-CN-dppz (bottom).

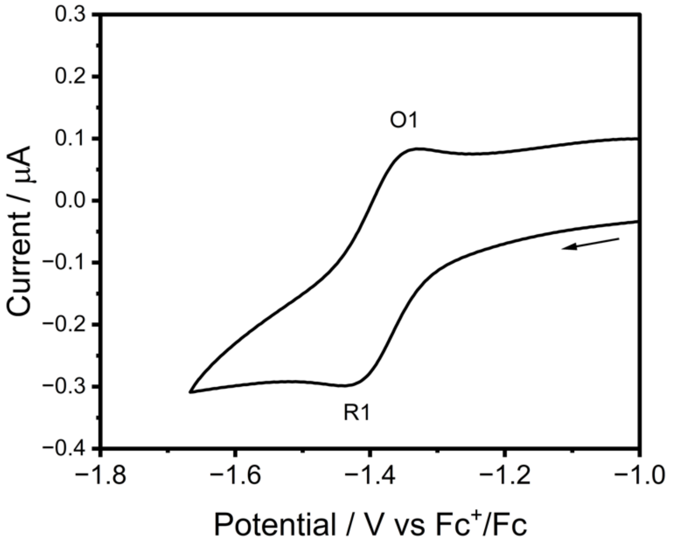

Figure S1. Cyclic voltammogram of 11-CN-dppz in PrCN/TBAH, showing its reversible reduction to the radical anion (at R1). Experimental conditions: Pt microdisc working electrode, T =298 K, v = 100 mV s^{-1}.

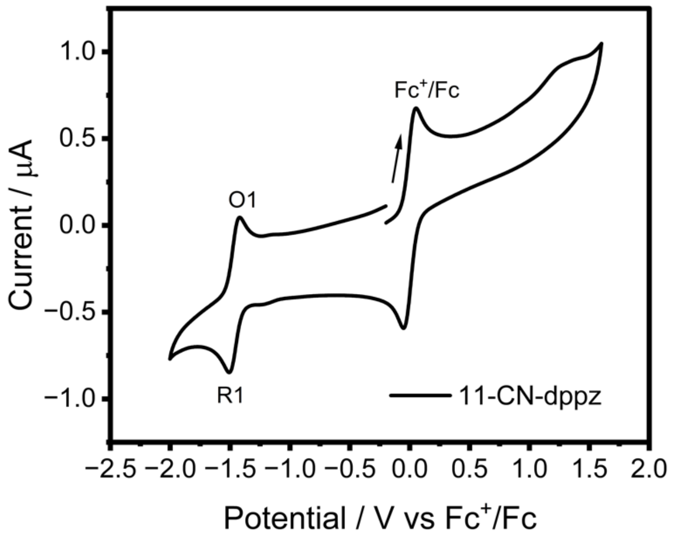

Figure S2. Cyclic voltammogram of 11-CN-dppz in DCM/TBAH, showing its reversible reduction to the radical anion (at R1). Experimental conditions: Pt microdisc working electrode, T =298 K, v = 100 mV s^{-1}.

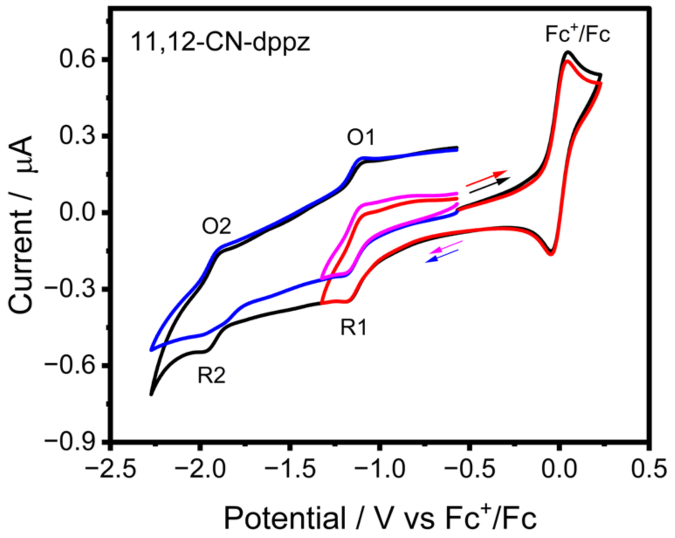

Figure S3. Cyclic voltammograms of 11,12-CN-dppz in PrCN/TBAH, showing the stepwise reductions to the corresponding radical anion (at R1) and dianion (at R2). Experimental conditions: Pt microdisc working electrode, $T = 298$ K, $v = 100$ mV s^{-1}.

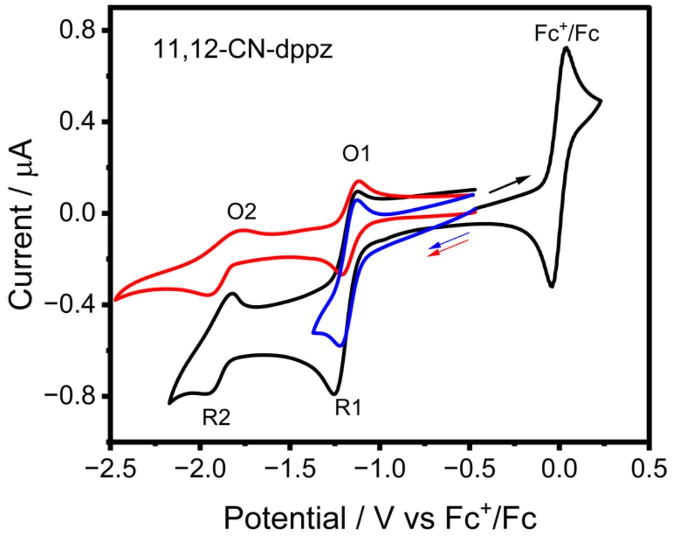

Figure S4. Cyclic voltammograms of 11,12-CN-dppz in DCM/TBAH, showing its stepwise reductions to the corresponding radical anion (at R1) and reactive dianion (at R2). Experimental conditions: Pt microdisc working electrode, $T = 298$ K, $v = 100$ mV s^{-1}.

2. Spectroelectrochemistry of free ligands

Using the IR SEC monitoring, the conversion of free 11-CN-dppz in DCM to the radical anion was accompanied by the replacement of the parent ν(CN) absorption band at 2232 cm^{-1} by a new intense feature at 2195 cm^{-1} (Figure S5). The absorbance was enhanced by a factor of four. These observations correlate with the DFT-calculated IR spectra (Figure S17) showing the same behavior and predicting an even larger enhancement by a factor of six. The corresponding UV-vis spectral changes reveal the characteristic $\pi^*\rightarrow\pi^*$ intra-ligand absorption of $[11\text{-CN-dppz}]^{\bullet-}$ at 620 nm (Figure S6). This absorption appears in the TDDFT-calculated UV-vis spectra at 578 nm (Figure S20). These reference data aid the assignment of electronic absorption of the ligand $[11\text{-CN-dppz}]^{\bullet-}$ in the reduced complexes (Figure 5 in the main text). The detailed assignment of electronic transitions in the UV-vis spectra of 11-CN-dppz and $[11\text{-CN-dppz}]^{\bullet-}$ (Figure S6) is given in Tables S1 and S2 linked to Figures S21 and S23, respectively.

The IR spectral changes observed during the reduction of free 11,12-CN-dppz to $[11,12\text{-CN-dppz}]^{\bullet-}$ (Figure S7) illustrate, similarly to free 11-CN-dppz, a comparably large red shift of the parent ν_s(CN) band at 2240 cm^{-1} to a smaller wavenumber (2218 cm^{-1}) and strong enhancement of its intensity. The IR spectrum of $[11,12\text{-CN-dppz}]^{\bullet-}$ also shows the better resolved asymmetric ν_{as}(CN) mode at 2204 cm^{-1}. Interestingly, $[11,12\text{-CN-dppz}]^{\bullet-}$ exhibits less ν_s(CN) enhancement compared with ν(CN) of $[11,12\text{-CN-dppz}]^{\bullet-}$, but this is predicted to be a property of the free ligands due to their delocalization. This correlates with the DFT-calculated IR spectra (Figure S26). The calculated ν_s(CN) and ν_{as}(CN) band maxima are separated only by 10 cm^{-1} and ν_{as}(CN) therefore remains unresolved. The theoretical IR ν_s(CN) absorption of 11,12-CN-dppz shows again a greater enhancement upon the $1e^-$ reduction than observed experimentally, which is ascribed to the delocalization of the phenazine distal ring. Similar to the monosubstituted dppz, the experimental UV-vis absorptions of 11,12-CN-dppz and $[11,12\text{-CN-dppz}]^{\bullet-}$ (Figure S8) are analyzed with TDDFT and assigned in Tables S3 and S4 linked to Figures S28 and S30, respectively.

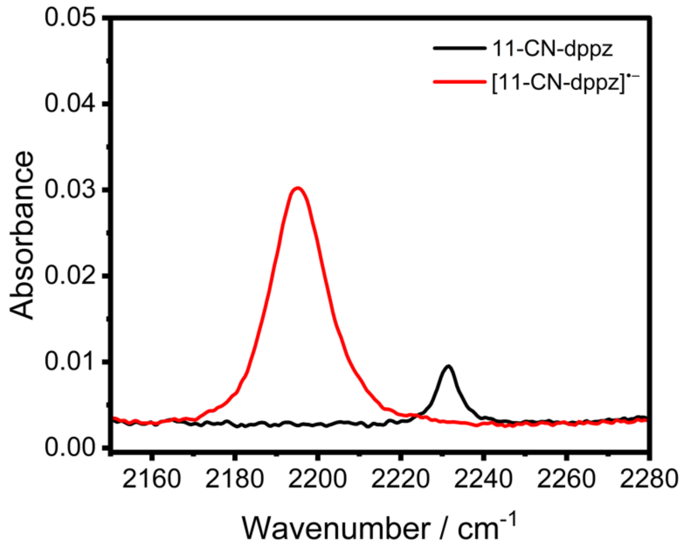

Figure S5. IR spectra of 11-CN-dppz (black curve) and [11-CN-dppz]•− (red curve) generated by electrochemical reduction within an OTTLE cell. Experimental conditions: Pt minigrid, DCM/TBAH, T = 298 K.

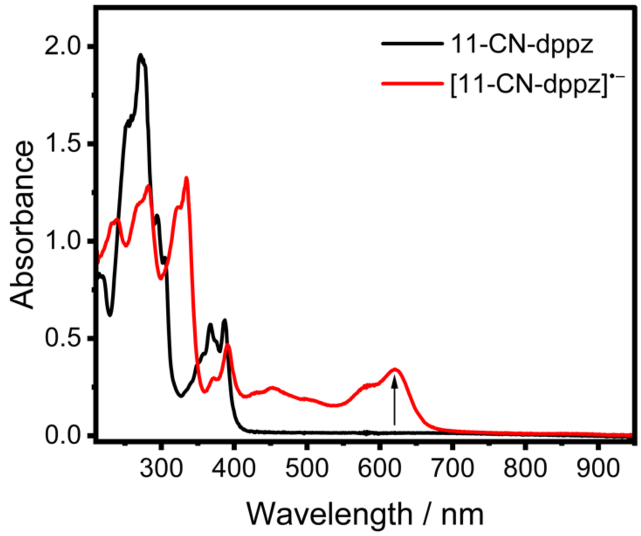

Figure S6. UV-vis absorption spectra of 11-CN-dppz (black curve) and [11-CN-dppz]•− (red curve) generated by electrochemical reduction within an OTTLE cell. Experimental conditions: Pt minigrid working electrode, DCM/TBAH, T = 298 K.

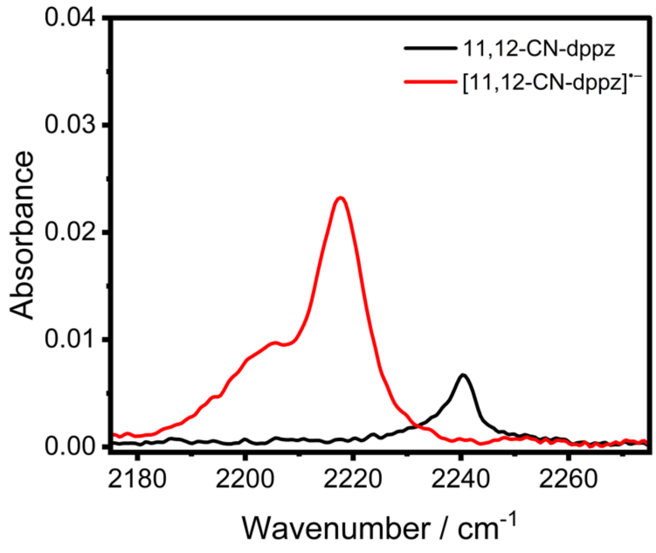

Figure S7. IR spectra of 11,12-CN-dppz (black curve) and [11,12-CN-dppz]•− (red curve) generated by electrochemical reduction within an OTTLE cell. Experimental conditions: Pt minigrid, DCM/TBAH, $T = 298$ K.

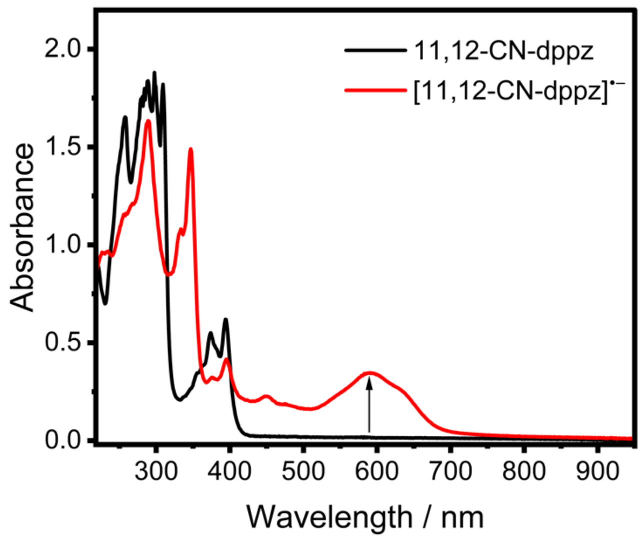

Figure S8. UV-vis absorption spectra of 11,12-CN-dppz (black curve) and [11,12-CN-dppz]•− (red curve) generated by electrochemical reduction within an OTTLE cell. Experimental conditions: Pt minigrid working electrode, DCM/TBAH, $T = 298$ K.

3. Cyclic voltammetry of studied complexes in PrCN

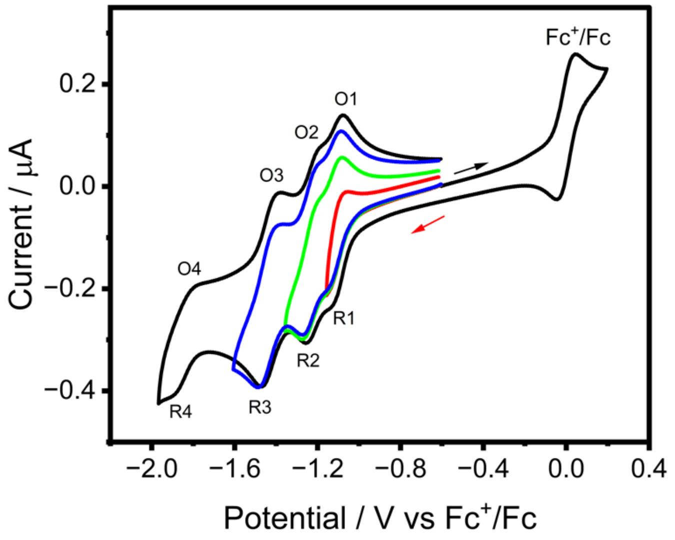

Figure S9. Cyclic voltammogram of $[Ru(tap)_2(11\text{-}CN\text{-}dppz)]^{2+}$ in PrCN/TBAH showing the reversible reductions to the corresponding cation (at R1), neutral complex (at R2), anion (at R3) and dianion (at R4). Experimental conditions: Pt microdisc working electrode, T = 298 K, v = 100 mV s^{-1}.

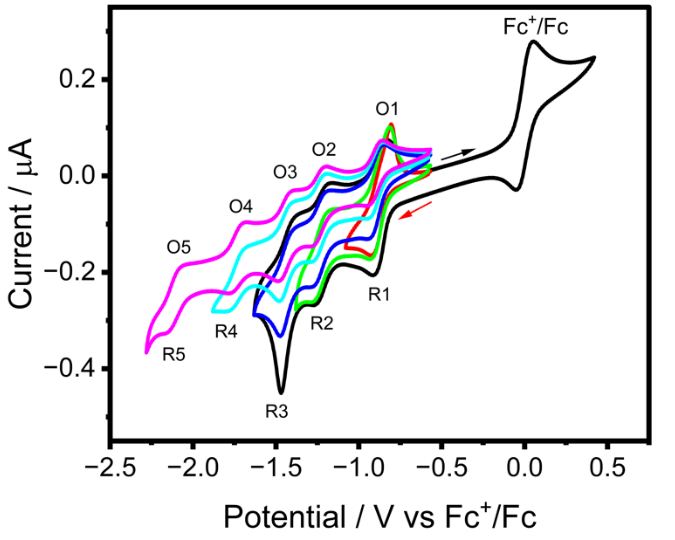

Figure S10. Cyclic voltammogram of $[Ru(tap)_2(11,12\text{-}CN\text{-}dppz)]^{2+}$ in PrCN/TBAH, showing the consecutive reduction to cationic (at R1), neutral (at R2), anionic (at R3), dianionic (at R4) and trianionic (at R5) species. Experimental conditions: Pt microdisc working electrode, T = 298 K, v = 100 mV s^{-1}.

4. Differential pulse voltammetry of studied complexes

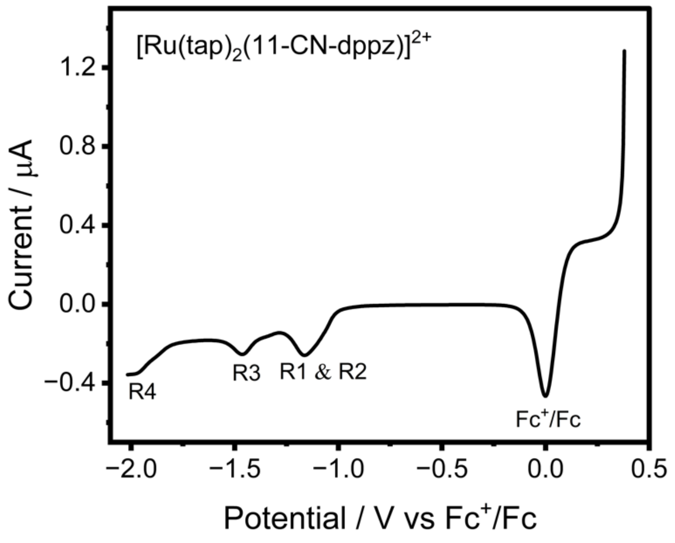

Figure S11. Differential pulse voltammogram (reductive branch) of $[Ru(tap)_2(11\text{-}CN\text{-}dppz)]^{2+}$ in DCM/TBAH, showing the consecutive reduction to cationic (at R1), neutral (at R2), anionic (at R3), and dianionic (at R4) species. Experimental conditions: Pt microdisc working electrode, T = 298 K, f = 10 Hz and t_p = 25 mV.

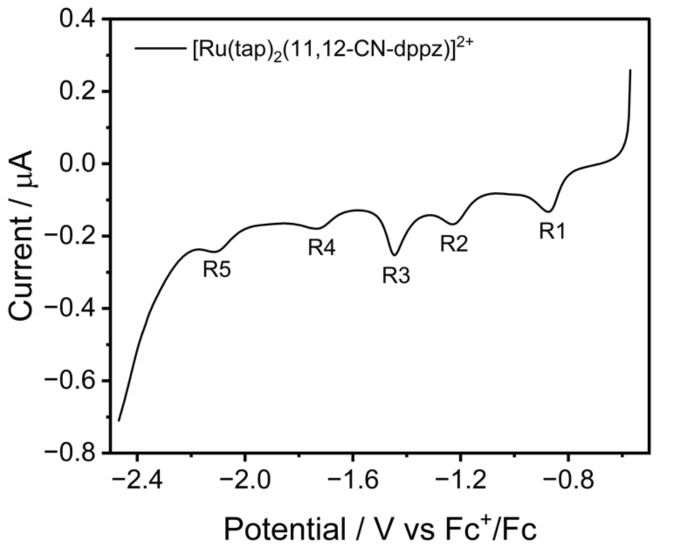

Figure S12. Differential pulse voltammogram (reductive branch) of $[Ru(tap)_2(11,12\text{-}CN\text{-}dppz)]^{2+}$ in PrCN/TBAH, showing the consecutive reduction to cationic (at R1), neutral (at R2), anionic (at R3), dianionic (at R4) and trianionic (at R5) species. The sixth consecutive reduction (at R6) remains unresolved. Experimental conditions: Pt microdisc working electrode, T = 298 K, f = 10 Hz and t_p = 25 mV.

5. Spectroelectrochemistry of studied complexes

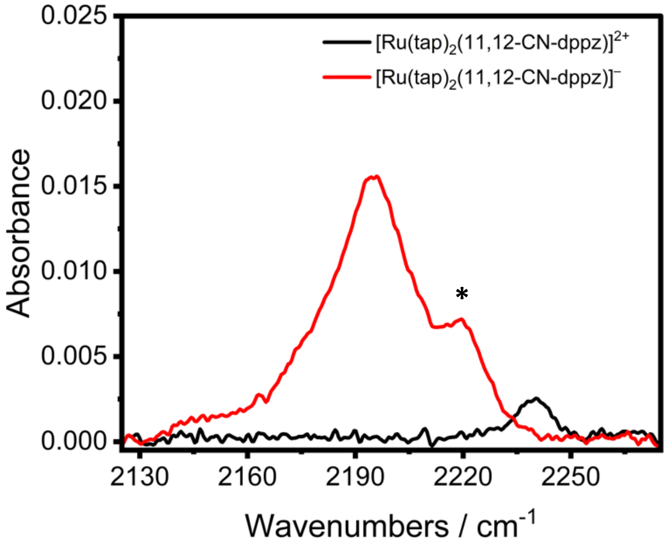

Figure S13. IR spectra of $[Ru(tap)_2(11,12\text{-}CN\text{-}dppz)]^{2+}$ (black curve) and the unassigned secondary species resulting from the third consecutive, irreversible $1e^-$ reduction of the parent complex (at R3 in the CV scan). The asterisk denotes an absorption band that remains unchanged during the reoxidation. Experimental conditions: Pt minigrid working electrode, an OTTLE cell, DCM/TBAH, $T = 298$ K.

6. NMR spectra of synthesized compounds

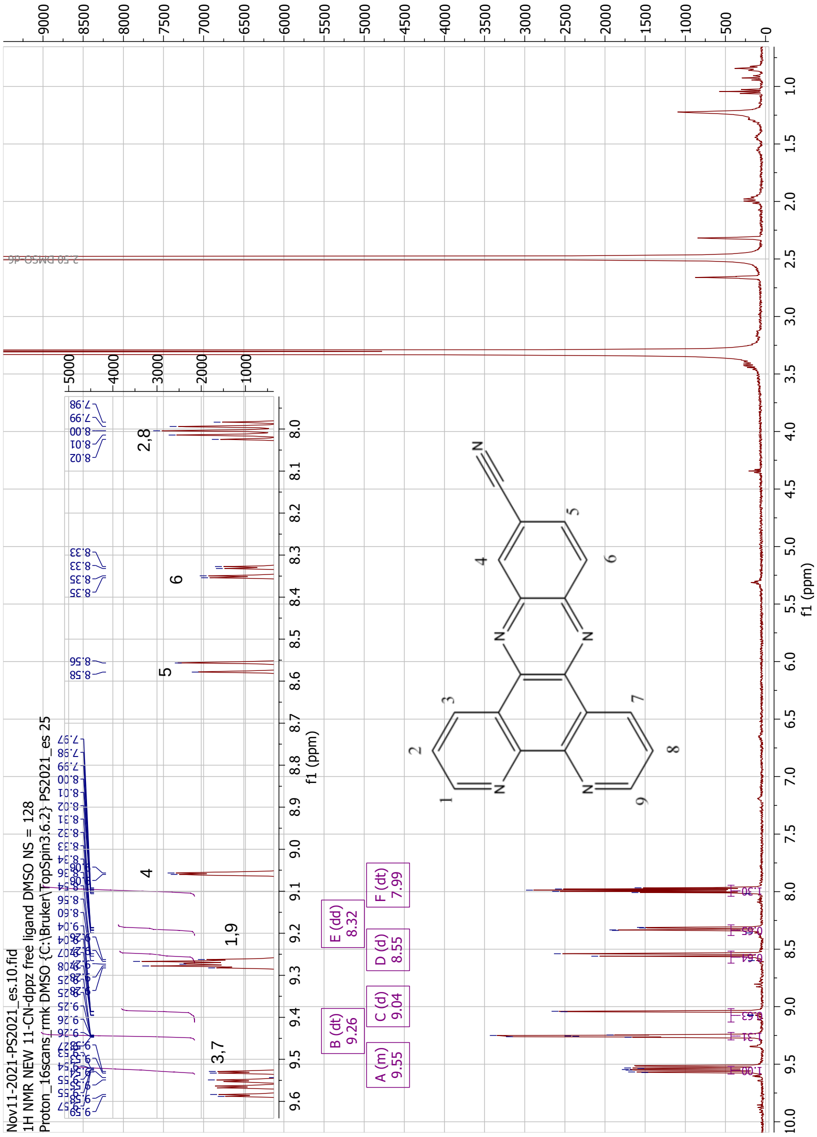

Figure S14. ^{1}H NMR (400 MHz, TMS, DMSO-d_6) spectrum of 11-CN-dppz.

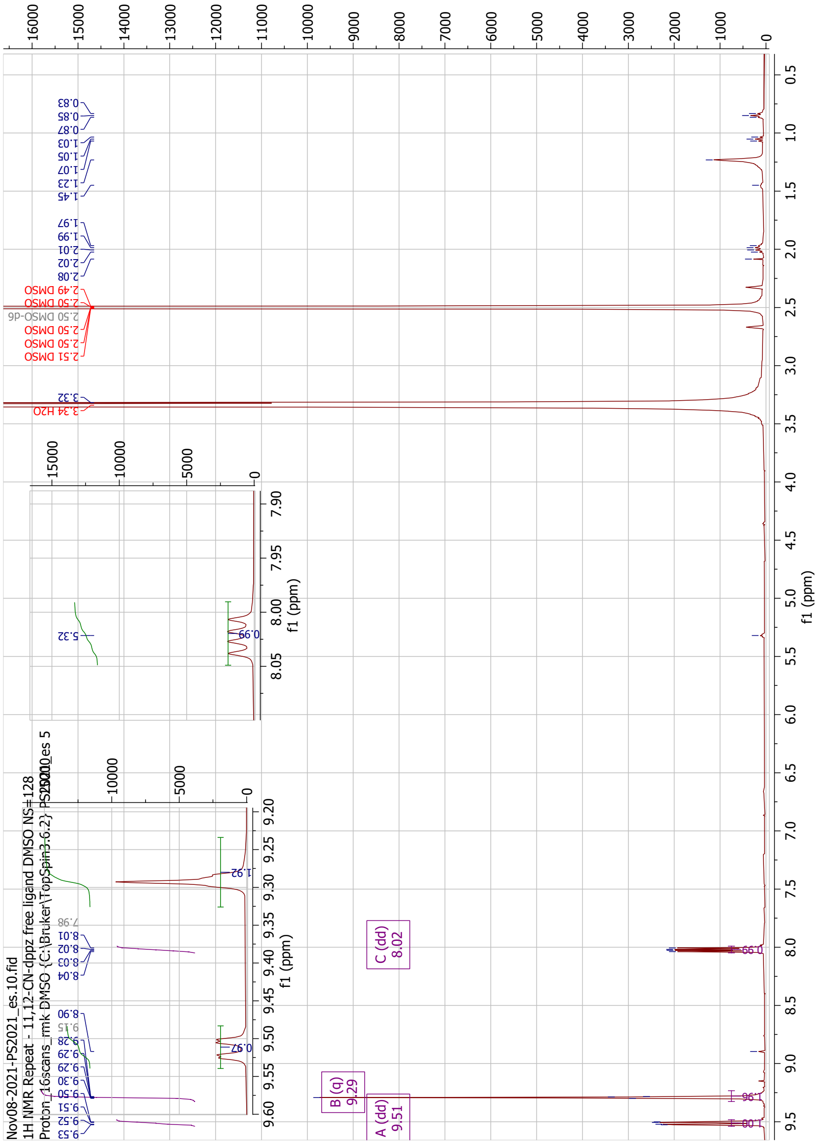

Figure S15. ^{1}H NMR (400 MHz, TMS, DMSO-d_6) spectrum of 11,12-CN-dppz.

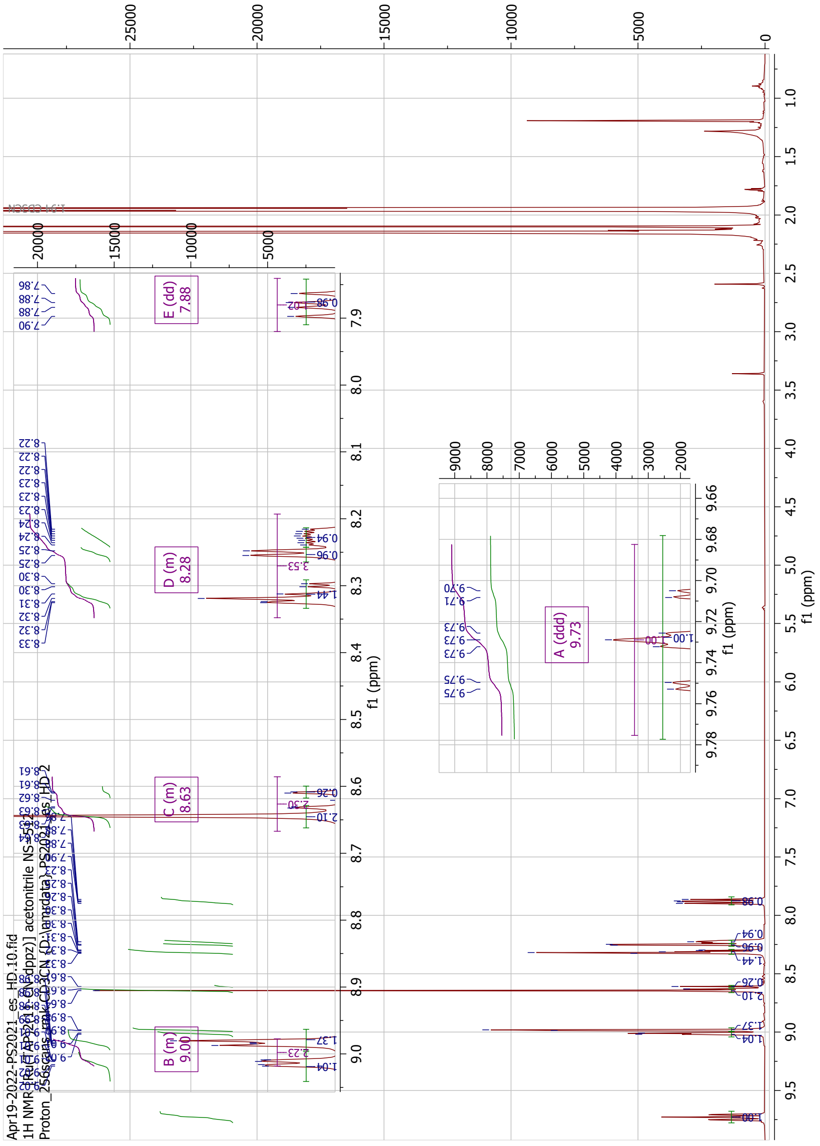

Figure S16. ^{1}H NMR (400 MHz, TMS, CD_3CN) spectrum of $[Ru(tap)_2(11\text{-}CN\text{-}dppz)]^{2+}$.

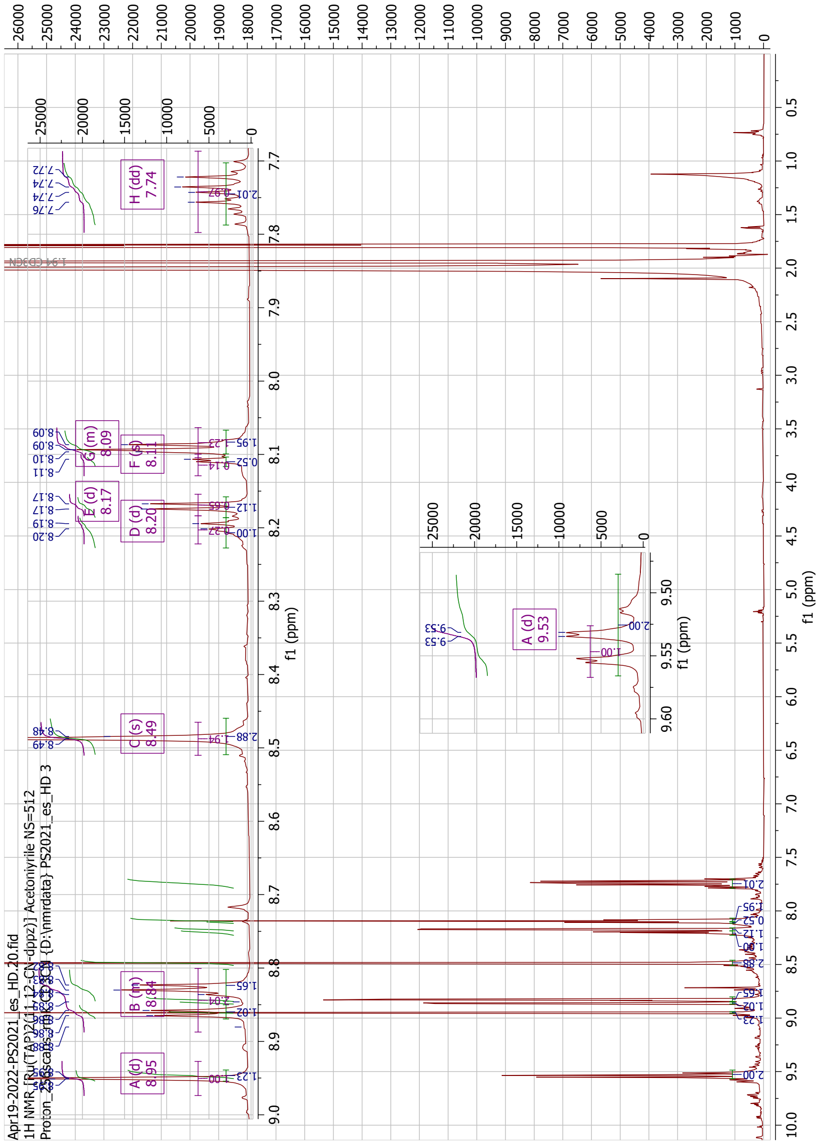

Figure S17. ^{1}H NMR (400 MHz, TMS, CD_3CN) spectrum of $[Ru(tap)_2(11,12\text{-}CN\text{-}dppz)]^{2+}$.

7. DFT and TDDFT calculations on 11-CN-dppz and 11,12-CN-dppz

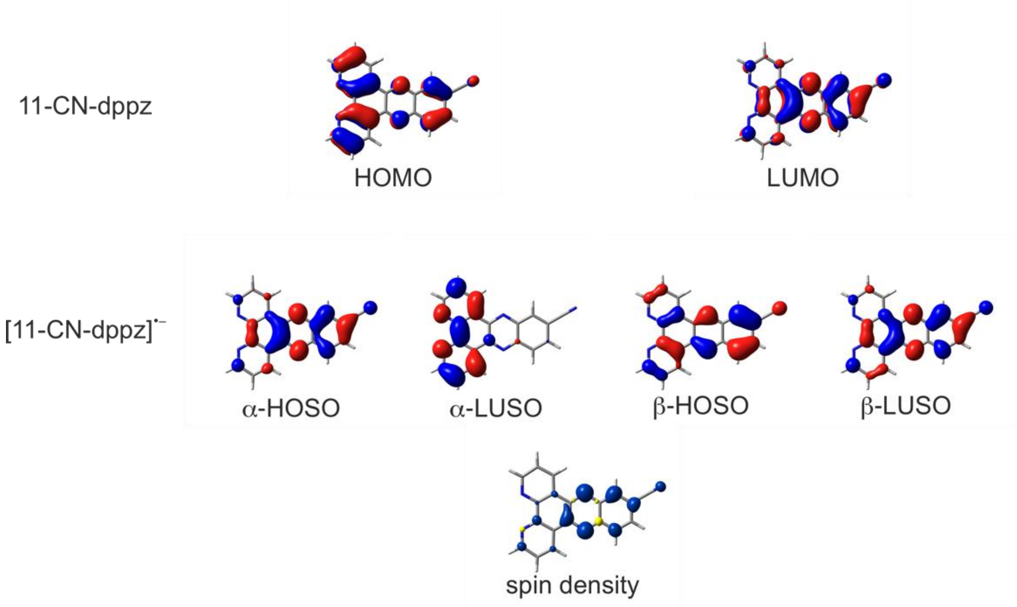

Figure S18. Frontier molecular (spin-) orbitals of [11-CN-dppz]$^{0/\bullet-}$ and spin density distribution in [11-CN-dppz]$^{\bullet-}$.

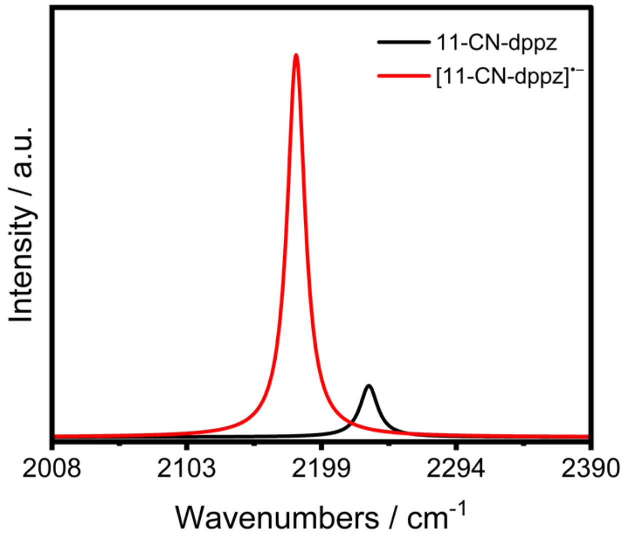

Figure S19. DFT-calculated IR spectra of 11-CN-dppz (black curve) and [11-CN-dppz]$^{\bullet-}$ (red curve).

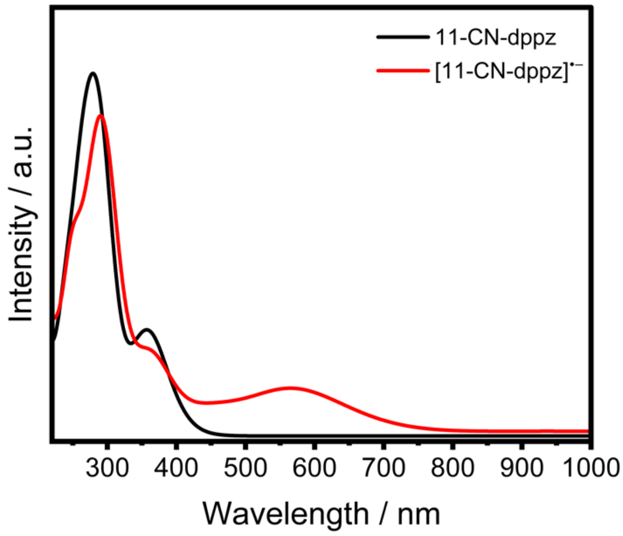

Figure S20. TDDFT-calculated UV-vis absorption spectra of 11-CN-dppz (black curve) and [11-CN-dppz]•− (red curve).

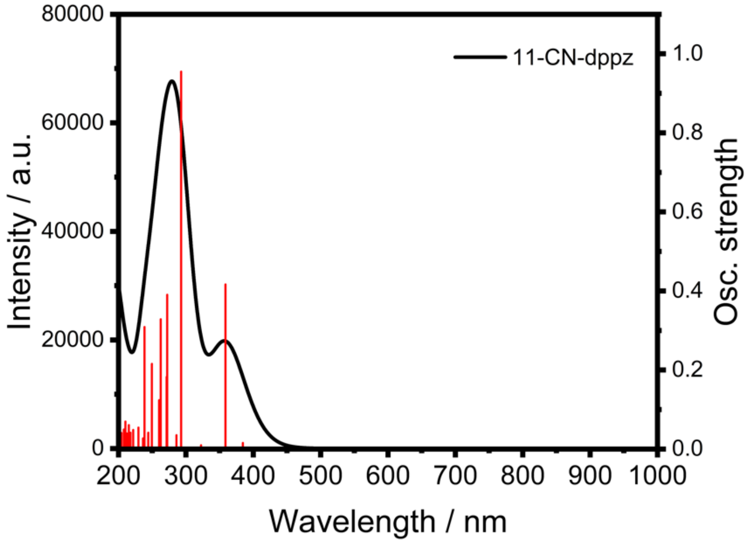

Figure S21. TDDFT-calculated UV-vis absorption spectrum of 11-CN-dppz.

Table S1. Major electronic excitations in 11-CN-dppz determined by TDDFT calculations.

Compound	Wavelength (nm) [a]	Osc. Str. (*f*)	Major contributions (%) [b]
**11-CN-dppz**	384 (387, 368)	0.0156	HOMO → LUMO (69)
	359 (387, 368)	0.4164	HOMO-1 → LUMO (66)<br>HOMO → LUMO+1 (22)
	293 (273)	0.9551	HOMO-1 → LUMO (20)<br>HOMO-1 → LUMO+2 (25)<br>HOMO → LUMO+1 (61)
	271 (273)	0.1827	HOMO-6 → LUMO (41)<br>HOMO-1 → LUMO+1 (52)
	263 (273)	0.3286	HOMO-6 → LUMO (49)<br>HOMO-1 → LUMO+1 (24)<br>HOMO → LUMO+2 (32)
	260 (273)	0.1237	HOMO-7 → LUMO (41)<br>HOMO-4 → LUMO+1 (37)<br>HOMO-1 → LUMO+2 (30)
	249 (252)	0.2156	HOMO-8 → LUMO (62)
	239 (252)	0.3088	HOMO-4 → LUMO+2 (67)

[a] Corresponding experimental absorption in brackets. [b] See Figure S22.

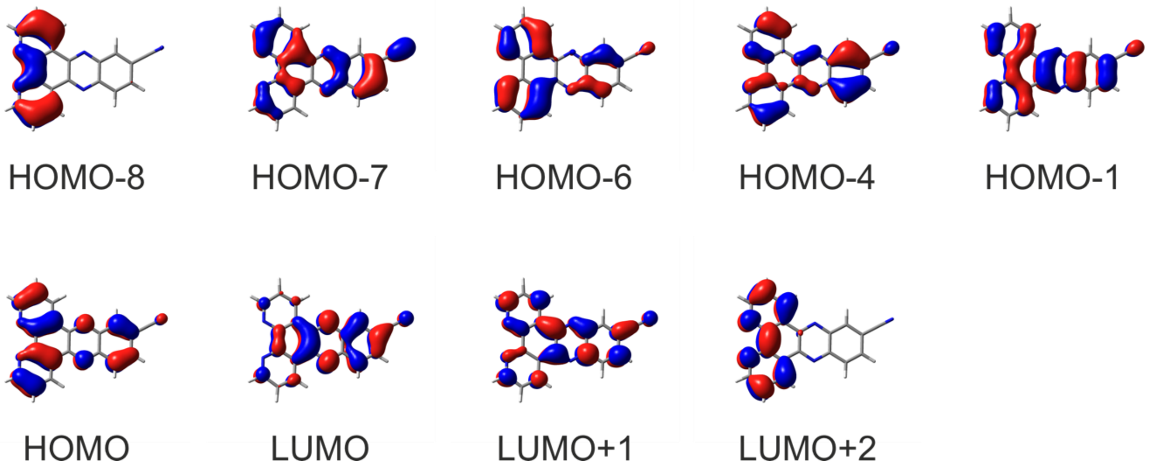

Figure S22. Molecular orbitals involved in electronic transitions of 11-CN-dppz (listed in Table S1).

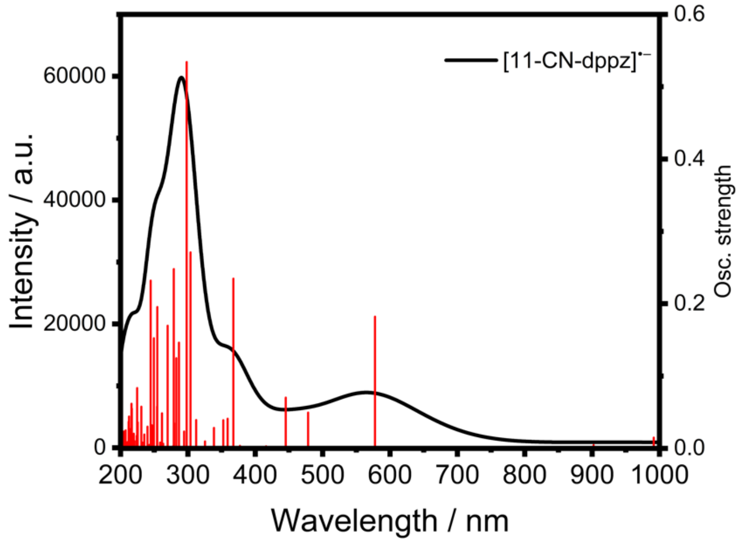

Figure S23. TDDFT-calculated UV-vis absorption spectrum of [11-CN-dppz]•−.

Table S2. Major electronic excitations in [11-CN-dppz]•− determined by TDDFT calculations.

Compound	Wavelength (nm) [a]	Osc. Str. (*f*)	Major contributions (%) [b]
[11-CN-dppz]•−	991	0.0149	α-HOSO → α-LUSO (33)<br>α-HOSO → α-LUSO+1 (93)
	578 (620)	0.1820	α-HOSO → α-LUSO+2 (97)
	479 (450)	0.0495	α-HOSO → α-LUSO+3 (87)<br>α-HOSO → α-LUSO+4 (26)<br>β-HOSO → β-LUSO (38)
	445 (450)	0.0702	α-HOSO → α-LUSO+3 (37)<br>β-HOSO-1 → β-LUSO+2 (24)<br>β-HOSO → β-LUSO (83)
	367 (390)	0.2346	α-HOSO-1 → α-LUSO+1 (27)<br>α-HOSO → α-LUSO+5 (38)<br>β-HOSO-1 → β-LUSO (83)
	304 (330)	0.2710	α-HOSO-5 → α-LUSO+1 (45)<br>α-HOSO-1 → α-LUSO+1 (37)<br>α-HOSO-1 → α-LUSO+2 (29)<br>β-HOSO-3 → β-LUSO (24)<br>β-HOSO-3 → β-LUSO+2 (39)<br>β-HOSO → β-LUSO+2 (23)<br>β-HOSO → β-LUSO+3 (28)
	298 (330)	0.5342	α-HOSO-2 → α-LUSO (33)<br>α-HOSO-1 → α-LUSO+1 (57)<br>β-HOSO-3 → β-LUSO (23)<br>β-HOSO-3 → β-LUSO+2 (21)<br>β-HOSO → β-LUSO+2 (37)<br>β-HOSO → β-LUSO+3 (25)
	287 (330)	0.1462	α-HOSO-2 → α-LUSO (48)<br>α-HOSO-1 → α-LUSO (28)<br>β-HOSO-3 → β-LUSO+2 (32)

Compound	Wavelength (nm) [a]	Osc. Str. (*f*)	Major contributions (%) [b]
			β-HOSO-1 → β-LUSO+1 (48)
	283 (330)	0.1247	α-HOSO-5 → α-LUSO+1 (28)<br>α-HOSO-2 → α-LUSO (28)<br>α-HOSO-2 → α-LUSO+1 (43)<br>α-HOSO-1 → α-LUSO+1 (29)<br>α-HOSO → α-LUSO+10 (25)<br>β-HOSO-3 → β-LUSO (20)<br>β-HOSO-3 → β-LUSO+1 (29)<br>β-HOSO-1 → β-LUSO+1 (20)
	279 (330)	0.2479	α-HOSO-10 → α-LUSO (27)<br>α-HOSO-2 → α-LUSO (37)<br>α-HOSO-2 → α-LUSO+1 (23)<br>α-HOSO → α-LUSO+10 (35)<br>β-HOSO-9 → β-LUSO+1 (28)<br>β-HOSO-3 → β-LUSO+1 (20)<br>β-HOSO-1 → β-LUSO+2 (33)
	270 (330)	0.1696	α-HOSO-5 → α-LUSO (27)<br>α-HOSO-2 → α-LUSO+1 (41)<br>α-HOSO-1 → α-LUSO (22)<br>α-HOSO → α-LUSO+10 (51)<br>β-HOSO-1 → β-LUSO+2 (47)<br>β-HOSO-1 → β-LUSO+3 (20)
	255 (281)	0.1955	α-HOSO-5 → α-LUSO (36)<br>α-HOSO-5 → α-LUSO+2 (21)<br>α-HOSO-1 → α-LUSO+3 (24)<br>β-HOSO-6 → β-LUSO+1 (26)<br>β-HOSO-3 → β-LUSO+1 (53)<br>β-HOSO → β-LUSO+4 (29)
	250 (281)	0.1523	α-HOSO-5 → α-LUSO (45)<br>α-HOSO-1 → α-LUSO+2 (38)<br>β-HOSO-8 → β-LUSO (30)<br>β-HOSO-3 → β-LUSO+2 (23)<br>β-HOSO → β-LUSO+3 (37)<br>β-HOSO → β-LUSO+4 (31)
	245 (281)	0.2319	α-HOSO-5 → α-LUSO (21)<br>α-HOSO-5 → α-LUSO+2 (20)<br>α-HOSO-2 → α-LUSO+2 (21)<br>α-HOSO-1 → α-LUSO+4 (25)<br>β-HOSO-8 → β-LUSO (37)<br>β-HOSO-6 → β-LUSO (44)<br>β-HOSO-3 → β-LUSO+1 (26)<br>β-HOSO-3 → β-LUSO+3 (21)<br>β-HOSO → β-LUSO+5 (20)

[a] Corresponding experimental absorption in brackets. [b] See Figure S24.

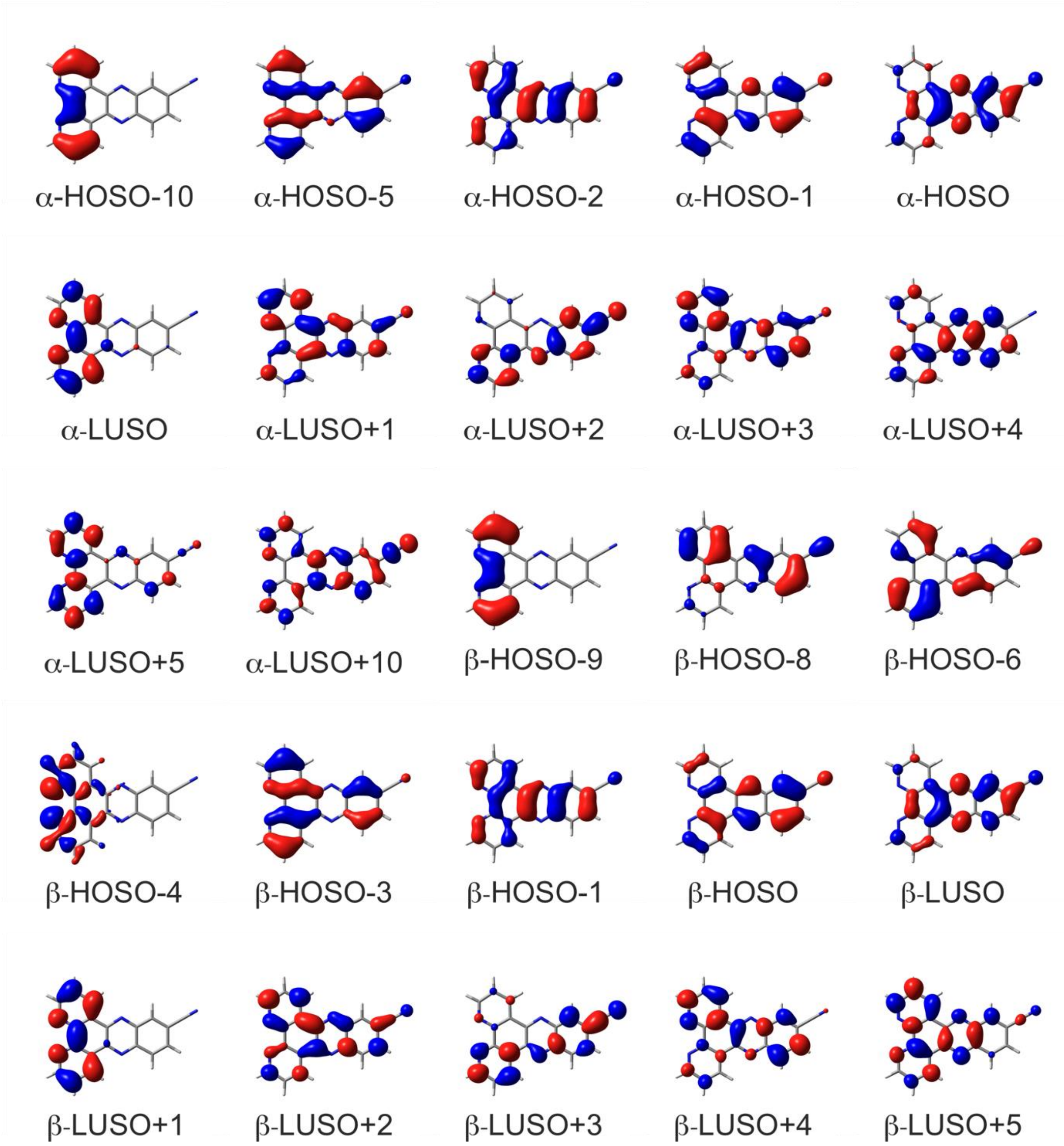

Figure S24. Molecular spin-orbitals involved in electronic transitions of [11-CN-dppz]$^{\bullet-}$ (listed in Table S2).

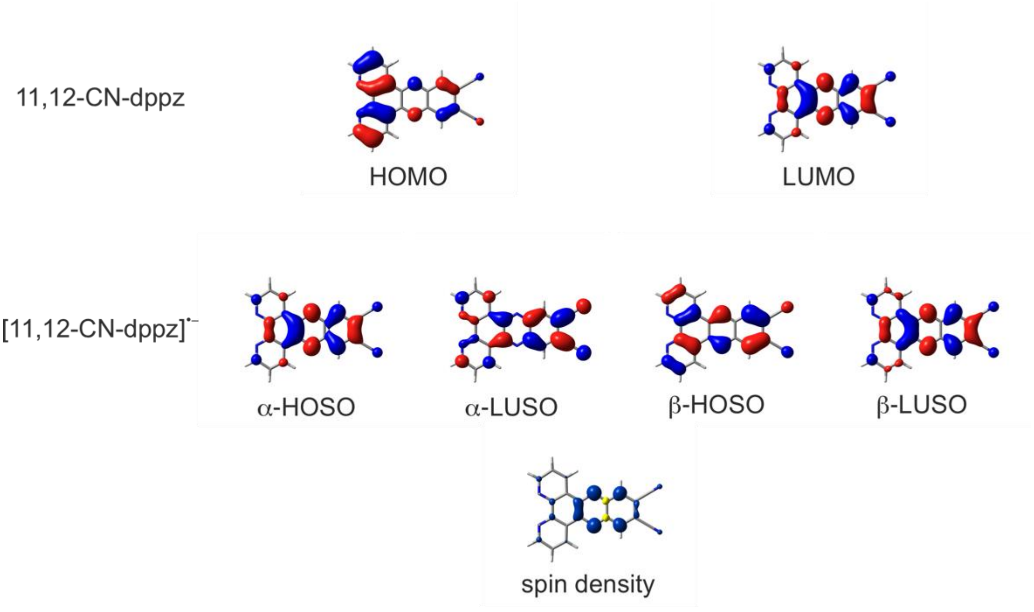

Figure S25. Frontier molecular (spin-) orbitals of [11,12-CN-dppz]$^{0/•−}$ and the spin density distribution in [11,12-CN-dppz]$^{•−}$.

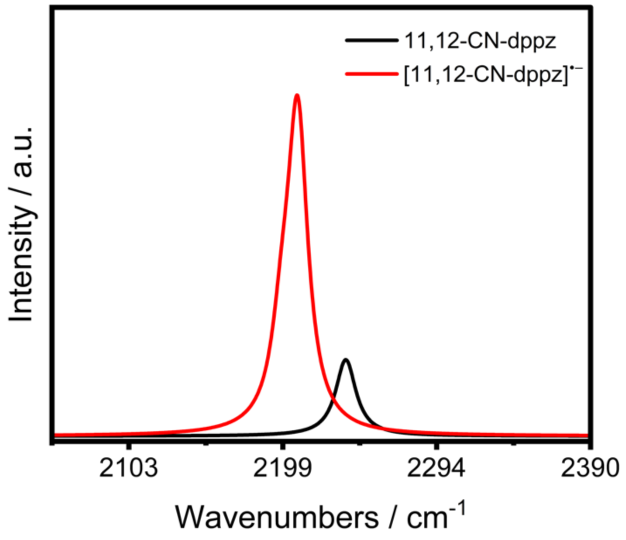

Figure S26. DFT-calculated IR spectra (ν(CN) region) of 11,12-CN-dppz (black curve) and reduced [11,12-CN-dppz]$^{•−}$ (red curve).

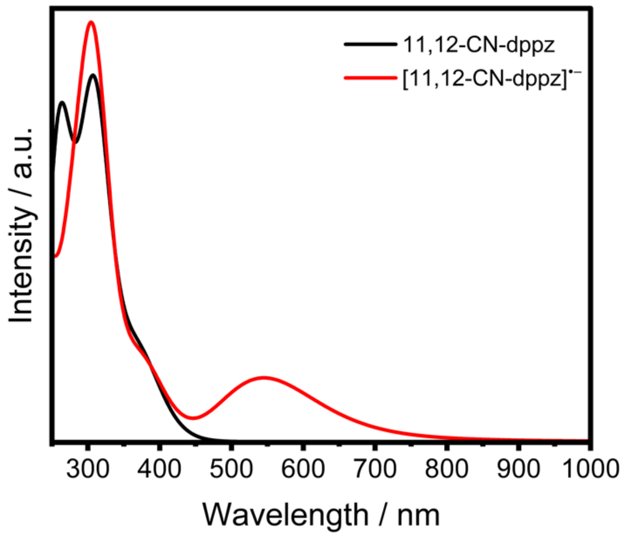

Figure S27. TDDFT-calculated UV-vis absorption spectra of 11,12-CN-dppz (black curve) and reduced [11,12-CN-dppz]•− (red curve).

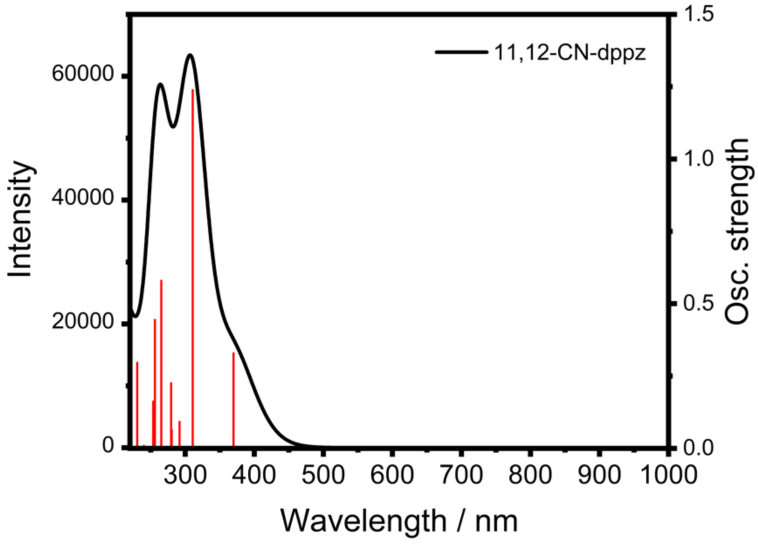

Figure S28. TDDFT-calculated UV-vis absorption spectrum of 11,12-CN-dppz.

Table S3. Major electronic excitations in 11,12-CN-dppz determined by TDDFT calculations.

Compound	Wavelength (nm) [a]	Osc. Str. (*f*)	Major contributions (%) [b]
11,12-CN-dppz	402 (394)	0.0057	HOMO → LUMO (70)
	370 (394,373)	0.3291	HOMO-1 → LUMO (65)<br>HOMO → LUMO+1 (26)
	311 (308)	1.2388	HOMO-1 → LUMO (26)<br>HOMO → LUMO+1 (64)
	280 (290)	0.2257	HOMO-8 → LUMO (25)<br>HOMO-7 → LUMO (27)<br>HOMO-4 → LUMO+1 (53)<br>HOMO-1 → LUMO+2 (22)
	265 (257)	0.5799	HOMO-6 → LUMO (41)<br>HOMO-1 → LUMO+1 (20)<br>HOMO → LUMO+1 (64)
	256 (257)	0.4442	HOMO-8 → LUMO (54)<br>HOMO-1 → LUMO+2 (34)
	254 (257)	0.1621	HOMO-7 → LUMO (44)<br>HOMO-4 → LUMO+1 (32)<br>HOMO-1 → LUMO+2 (20)<br>HOMO → LUMO+3 (36)
	231 (257)	0.2959	HOMO-4 → LUMO+2 (49)<br>HOMO-1 → LUMO+3 (47)
	213 (210)	0.1256	HOMO-6 → LUMO+2 (39)<br>HOMO-4 → LUMO+4 (32)<br>HOMO → LUMO+5 (41)
	212 (210)	0.2285	HOMO-11 → LUMO (22)<br>HOMO-4 → LUMO+3 (46)<br>HOMO-1 → LUMO+4 (31)

[a] Corresponding experimental absorption in brackets. [b] See Figure S29.

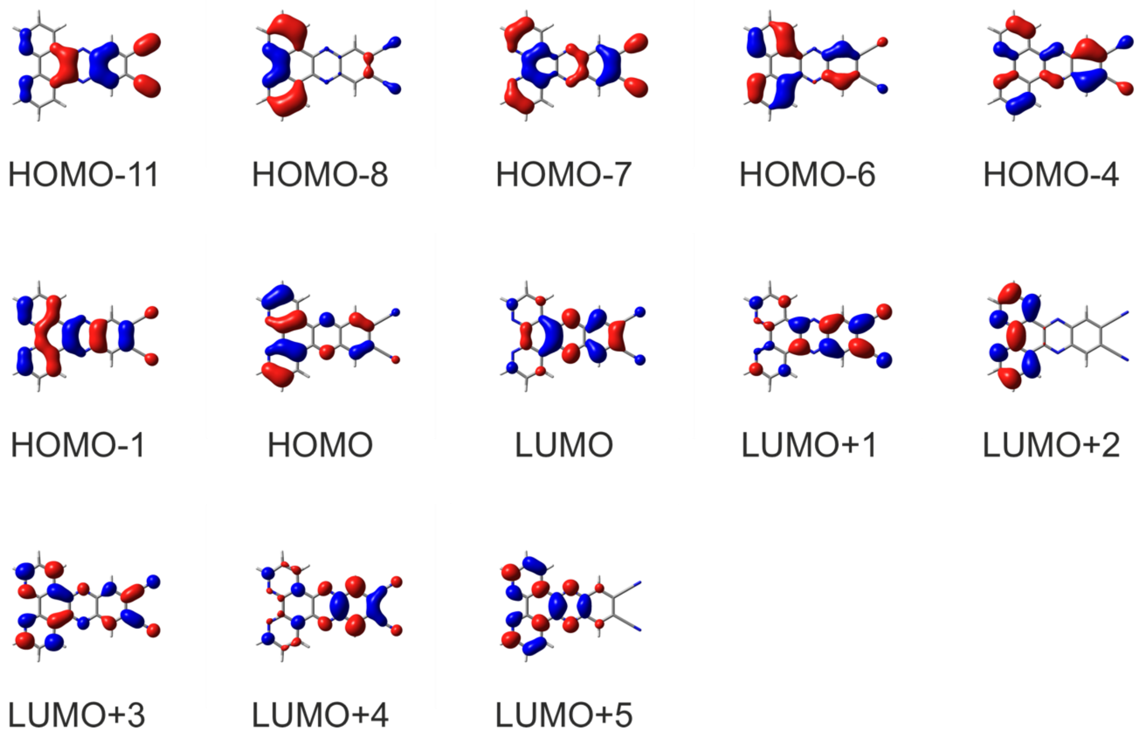

Figure S29. Molecular orbitals involved in electronic transitions of 11,12-CN-dppz (listed in Table S3).

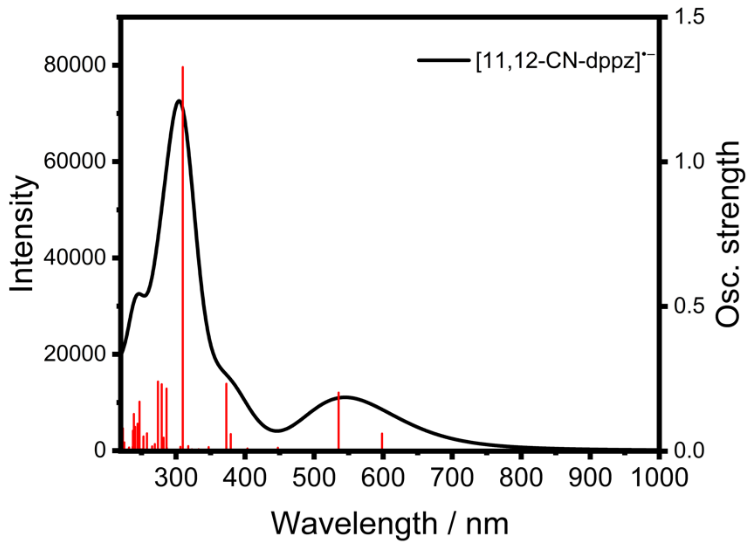

Figure S30. TDDFT-calculated UV-vis absorption spectrum of [11,12-CN-dppz]$^{•-}$.

Table S4. Major electronic excitations in [11,12-CN-dppz]$^{\bullet-}$ determined by TDDFT calculations.

Compound	Wavelength (nm) [a]	Osc. Str. (*f*)	Major contributions (%) [b]
[11,12-CN-dppz]$^{\bullet-}$	598 (589)	0.0607	α-HOSO → α-LUSO+2 (96)<br>β-HOSO → β-LUSO (21)
	536 (589)	0.2023	α-HOSO → α-LUSO+3 (97)
	373 (346)	0.2331	α-HOSO-1 → α-LUSO (27)<br>β-HOSO-1 → β-LUSO (91)
	310 (330)	1.3273	α-HOSO-2 → α-LUSO+1 (26)<br>α-HOSO-1 → α-LUSO (73)<br>β-HOSO-1 → β-LUSO (28)<br>β-HOSO → β-LUSO+1 (50)
	286 (289)	0.2165	α-HOSO-2 → α-LUSO (58)<br>α-HOSO → α-LUSO+8 (23)<br>β-HOSO-6 → β-LUSO (21)<br>β-HOSO-3 → β-LUSO+2 (31)<br>β-HOSO-1 → β-LUSO+1 (47)<br>β-HOSO → β-LUSO+2 (29)
	279 2(89)	0.2310	α-HOSO-10 → α-LUSO+1 (28)<br>α-HOSO-5 → α-LUSO (33)<br>α-HOSO-2 → α-LUSO+1 (62)<br>α-HOSO-2 → α-LUSO+3 (21)<br>β-HOSO-9 → β-LUSO+2 (28)
	274 (289)	0.2405	α-HOSO-5 → α-LUSO+1 (27)<br>α-HOSO-2 → α-LUSO (22)<br>α-HOSO-1 → α-LUSO+1 (40)<br>α-HOSO → α-LUSO+8 (53)<br>β-HOSO-3 → β-LUSO+2 (30)<br>β-HOSO-1 → β-LUSO+1 (36)
	247 (258)	0.1711	α-HOSO-5 → α-LUSO+1 (39)<br>α-HOSO-2 → α-LUSO (20)<br>α-HOSO-2 → α-LUSO+2 (20)<br>α-HOSO-1 → α-LUSO+3 (31)<br>α-HOSO-1 → α-LUSO+4 (23)<br>β-HOSO-8 → β-LUSO+1 (21)<br>β-HOSO-6 → β-LUSO (30)<br>β-HOSO-3 → β-LUSO+2 (34)<br>β-HOSO-3 → β-LUSO+4 (23)<br>β-HOSO-1 → β-LUSO+3 (35)
	239 (258)	0.1285	α-HOSO-9 → α-LUSO (23)<br>α-HOSO-7 → α-LUSO+1 (30)<br>α-HOSO-5 → α-LUSO+1 (49)<br>α-HOSO-2 → α-LUSO+2 (25)<br>α-HOSO-1 → α-LUSO+3 (44)<br>β-HOSO-8 → β-LUSO+1 (23)<br>β-HOSO-6 → β-LUSO (23)<br>β-HOSO → β-LUSO+4 (28)

[a] Corresponding experimental absorption in brackets. [b] See Figure S31.

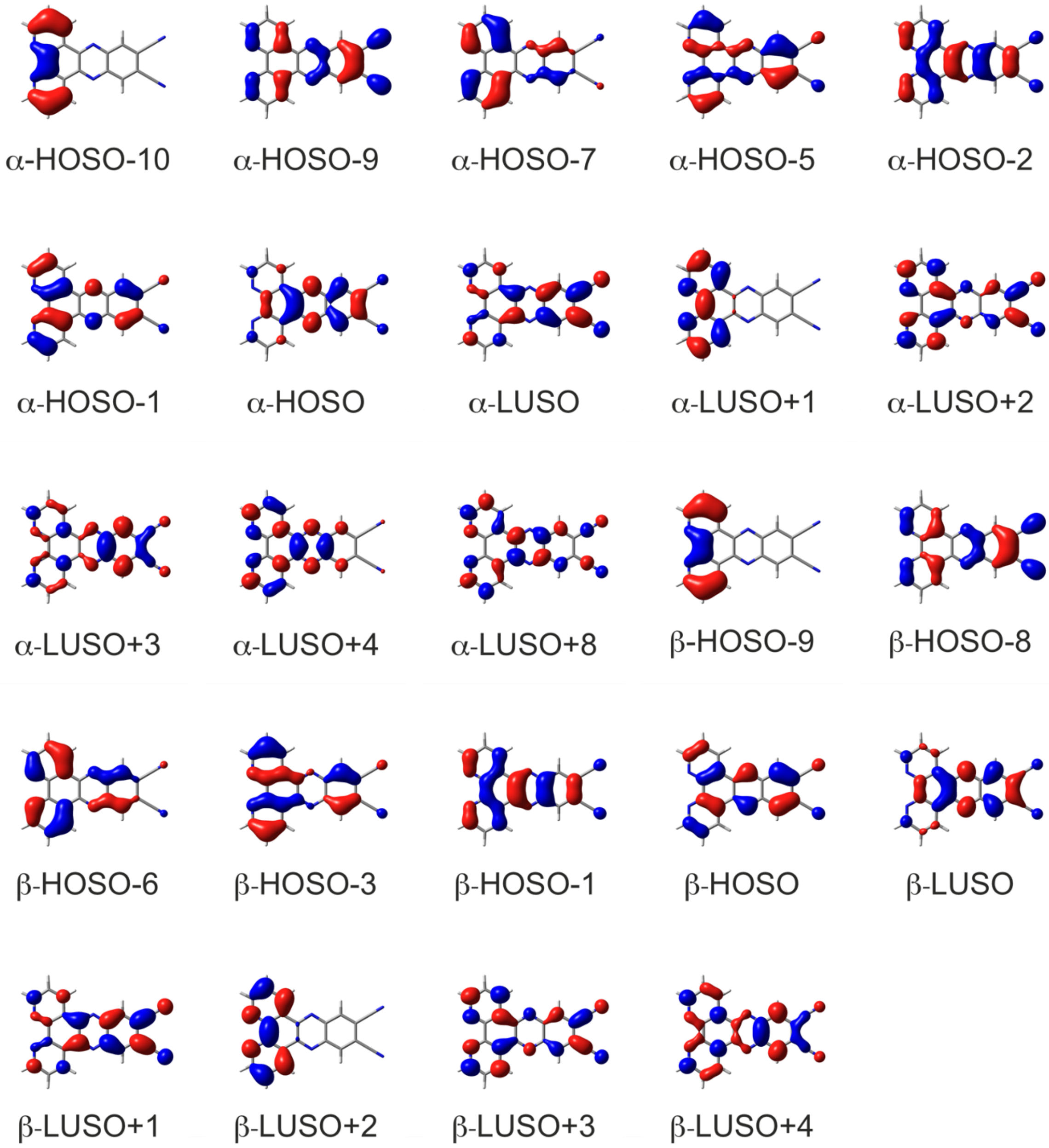

Figure S31. Molecular spin-orbitals involved in electronic transitions of $[11,12\text{-CN-dppz}]^{\bullet-}$ (listed in Table S4).

8. DFT and TDDFT calculations on studied complexes

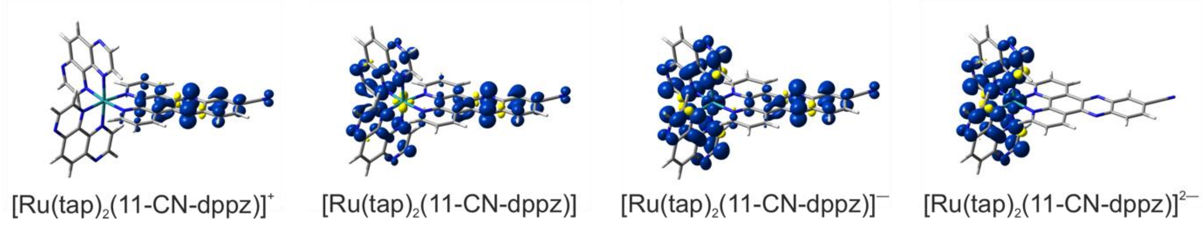

Figure S32. Distribution of spin densities in 1e$^{–}$ reduced (at R1; doublet) $[Ru(tap)_2(11\text{-}CN\text{-}dppz)]^{+}$, 2e$^{–}$ reduced (at R2; triplet) $[Ru(tap)_2(11\text{-}CN\text{-}dppz)]$, 3e$^{–}$ reduced (at R3; quadruplet) $[Ru(tap)_2(11\text{-}CN\text{-}dppz)]^{–}$ and 4e$^{–}$ reduced (at R4; triplet) $[Ru(tap)_2(11\text{-}CN\text{-}dppz)]^{2–}$.

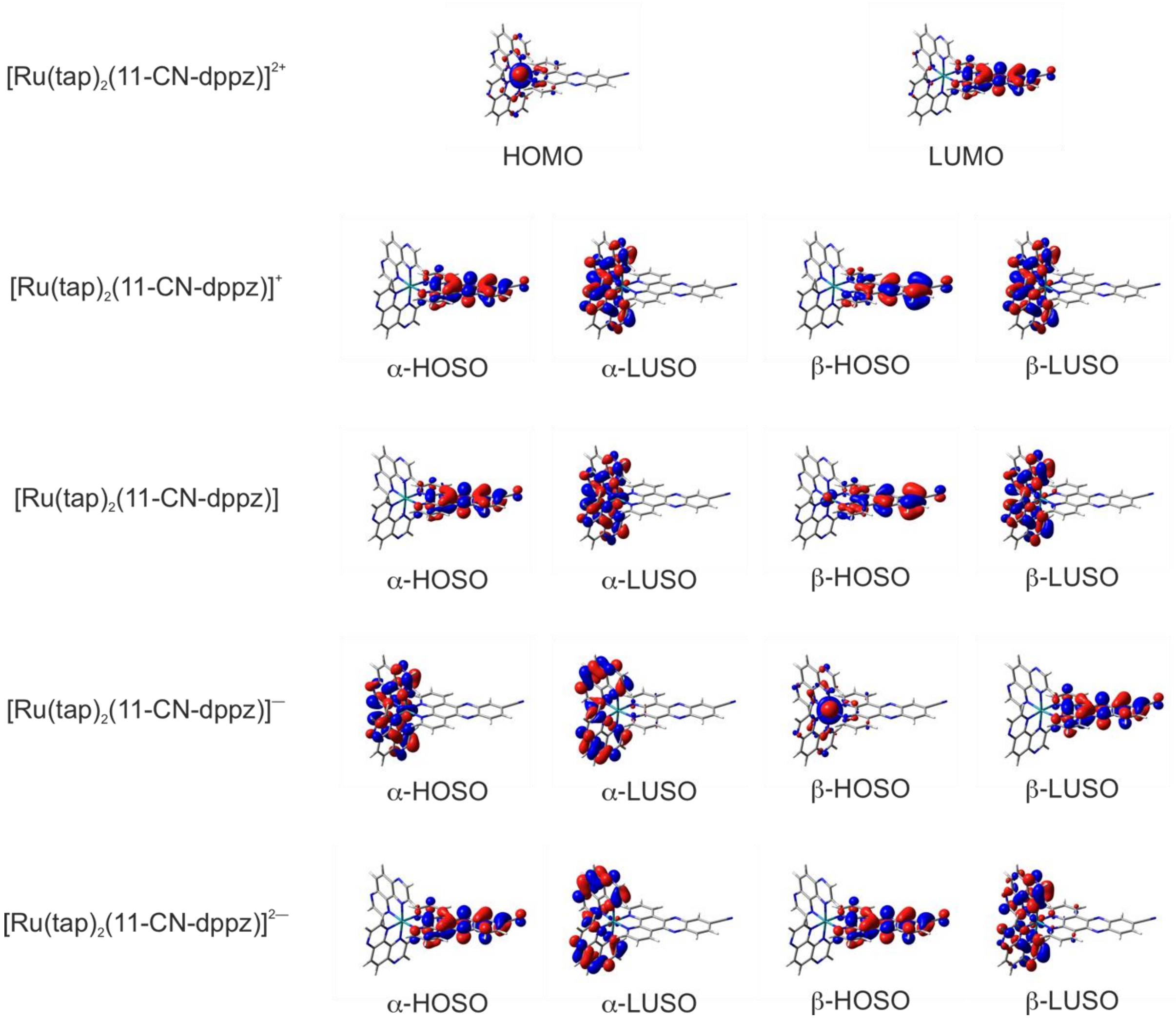

Figure S33. Frontier molecular (spin-) orbitals of $[Ru(tap)_2(11\text{-}CN\text{-}dppz)]^{2+/+/0/–/2–}$.

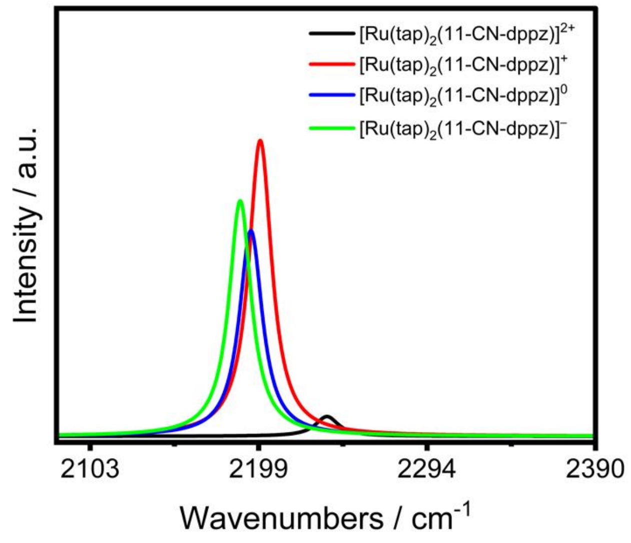

Figure S34. DFT-calculated IR spectra of $[Ru(tap)_2(11\text{-}CN\text{-}dppz)]^{2+}$ (black curve), $[Ru(tap)_2(11\text{-}CN\text{-}dppz)]^{+}$ (red curve), $[Ru(tap)_2(11\text{-}CN\text{-}dppz)]$ (blue curve) and $[Ru(tap)_2(11\text{-}CN\text{-}dppz)]^{-}$ (green curve).

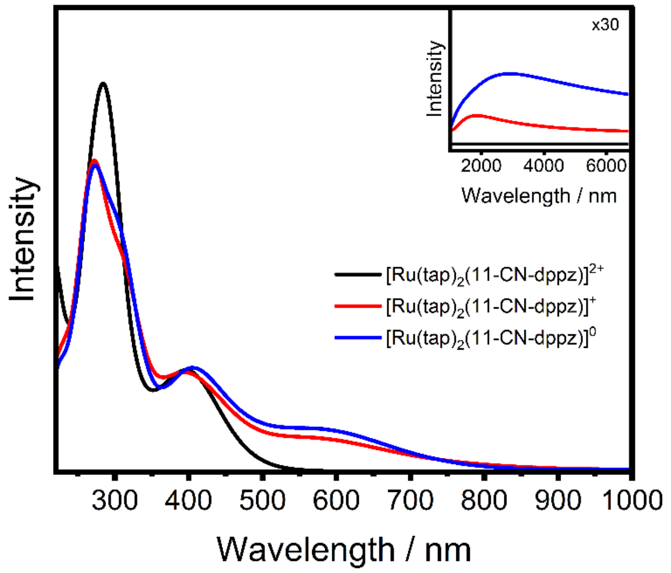

Figure S35. TDDFT-calculated UV-vis absorption spectra of $[Ru(tap)_2(11\text{-}CN\text{-}dppz)]^{2+}$ (black curve), 1e^{-} reduced $[Ru(tap)_2(11\text{-}CN\text{-}dppz)]^{+}$ (red curve) and 2e^{-} reduced $[Ru(tap)_2(11\text{-}CN\text{-}dppz)]$ (blue curve).

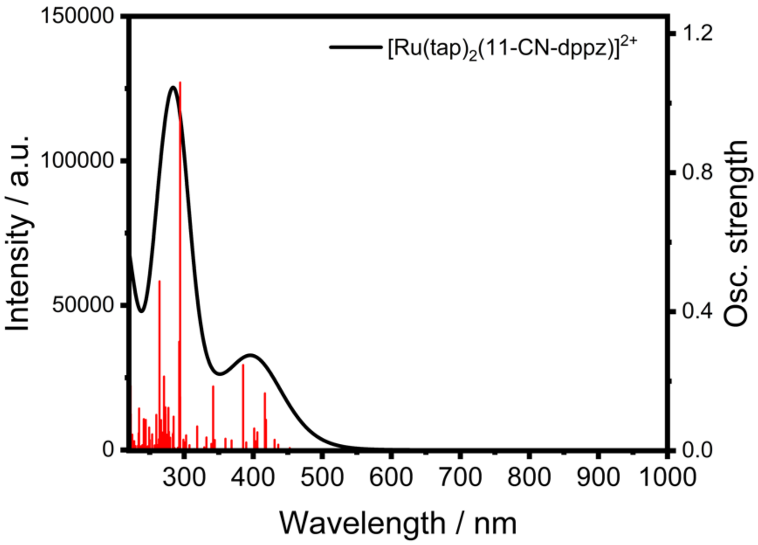

Figure S36. TDDFT-calculated UV-vis absorption spectrum of $[Ru(tap)_2(11\text{-}CN\text{-}dppz)]^{2+}$.

Table S5. Major electronic excitations in $[Ru(tap)_2(11\text{-}CN\text{-}dppz)]^{2+}$ determined by TDDFT calculations.

Compound	Wavelength (nm) *a*	Osc. Str. (*f*)	Major contributions (%) *b*
$[Ru(tap)_2(11\text{-}CN\text{-}dppz)]^{2+}$	453 (454sh)	0.0078	HOMO-1 → LUMO+1 (59)<br>HOMO-1 → LUMO+2 (22)
	436 (454sh)	0.0172	HOMO-2 → LUMO+1 (30)<br>HOMO → LUMO (48)<br>HOMO → LUMO+4 (28)<br>HOMO → LUMO+5 (23)
	431 (454sh)	0.0315	HOMO-2 → LUMO+1 (36)<br>HOMO-1 → LUMO+2 (23)<br>HOMO → LUMO (40)<br>HOMO → LUMO+4 (25)
	419 (414)	0.0892	HOMO-2 → LUMO+1 (26)<br>HOMO-1 → LUMO+3 (31)<br>HOMO → LUMO+4 (54)
	417 (414)	0.1652	HOMO-1 → LUMO (61)
	385 (414)	0.2468	HOMO-2 → LUMO+2 (32)<br>HOMO-2 → LUMO+3 (30)<br>HOMO-1 → LUMO+5 (40)
	342 (350)	0.1849	HOMO-4 → LUMO (48)<br>HOMO-3 → LUMO+6 (25)
	294.4 (277)	0.155	HOMO-8 → LUMO+1 (53)<br>HOMO → LUMO+7 (29)
	294.1 (277)	1.0596	HOMO-8 → LUMO+1 (21)<br>HOMO-4 → LUMO (24)<br>HOMO-3 → LUMO+6 (51)
	293 (277)	0.3140	HOMO-9 → LUMO+1 (37)<br>HOMO-7 → LUMO+4 (20)

Compound	Wavelength (nm) [a]	Osc. Str. (*f*)	Major contributions (%) [b]
			HOMO-6 → LUMO+3 (38)<br>HOMO-3 → LUMO+6 (22)
	277 (277)	0.1232	HOMO-9 → LUMO+2 (21)<br>HOMO-9 → LUMO+3 (29)<br>HOMO-6 → LUMO+5 (37)<br>HOMO-2 → LUMO+7 (23)
	273 (277)	0.1047	HOMO-10 → LUMO+4 (21)<br>HOMO → LUMO+8 (21)<br>HOMO → LUMO+13 (22)
	272.9 (277)	0.1247	HOMO-10 → LUMO+4 (22)<br>HOMO-2 → LUMO+7 (20)<br>HOMO → LUMO+8 (21)
	271 (277)	0.2134	HOMO-2 → LUMO+7 (33)<br>HOMO → LUMO+13 (22)
	264 (277)	0.4874	HOMO-8 → LUMO+5 (46)
	260 (277)	0.1033	HOMO-6 → LUMO+6 (21)<br>HOMO → LUMO+9 (48)
	235 (226)	0.1216	HOMO-16 → LUMO (59)
	222.6 (226)	0.1055	HOMO-15 → LUMO+3 (29)<br>HOMO-15 → LUMO+4 (37)<br>HOMO-6 → LUMO+7 (35)<br>HOMO → LUMO+16 (27)
	222.5	0.1866	HOMO-15 → LUMO+4 (28)<br>HOMO-6 → LUMO+7 (47)<br>HOMO → LUMO+16 (27)

[a] Corresponding experimental absorption in brackets. [b] See Figure S37.

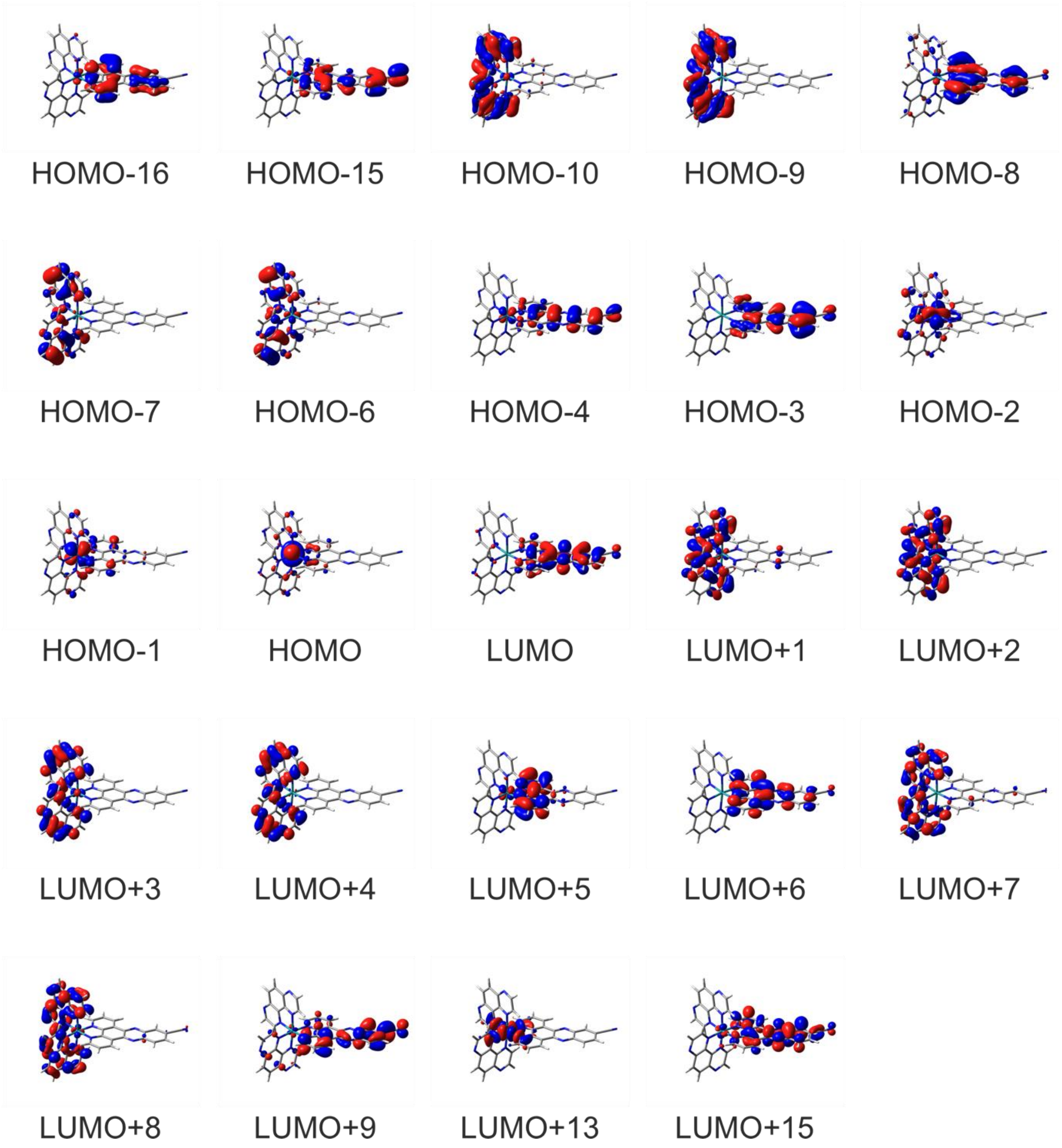

Figure S37. Molecular orbitals involved in electronic transitions of $[Ru(tap)_2(11\text{-}CN\text{-}dppz)]^{2+}$ (listed in Table S5).

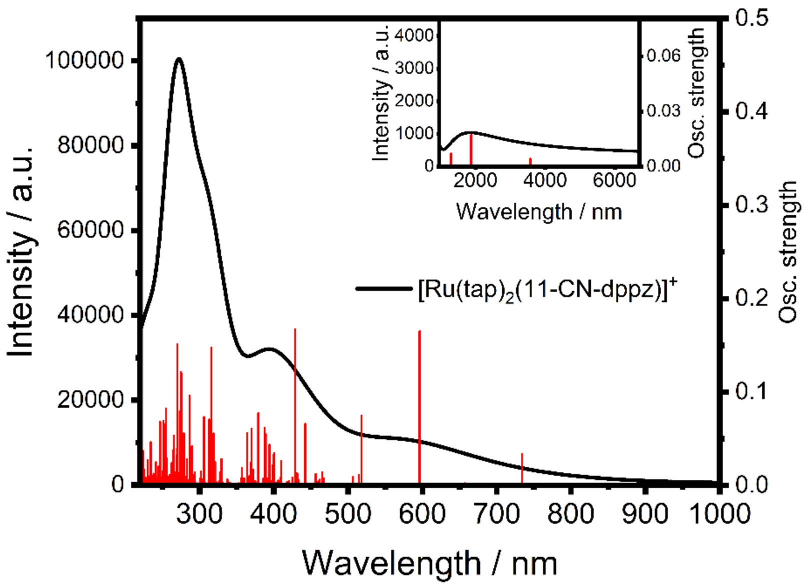

Figure S38. TDDFT-calculated UV-vis absorption spectrum of $[Ru(tap)_2(11\text{-}CN\text{-}dppz)]^+$.

Table S6. Major electronic excitations of $[Ru(tap)_2(11\text{-}CN\text{-}dppz)]^+$ determined by TDDFT calculations.

Compound	Wavelength (nm) [a]	Osc. Str. (*f*)	Major contributions (%) [b]
$[Ru(tap)_2(11\text{-}CN\text{-}dppz)]^+$	1876	0.0170	α-HOSO → α-LUSO+4 (99.6)
	734	0.0339	α-HOSO → α-LUSO+6 (99.6)
	596	0.1651	α-HOSO → α-LUSO+8 (24)<br>α-HOSO → α-LUSO+9 (36)<br>α-HOSO → α-LUSO+10 (84)<br>α-HOSO → α-LUSO+11 (24)
	518 (502)	0.0746	α-HOSO → α-LUSO+8 (29)<br>α-HOSO → α-LUSO+9 (40)<br>α-HOSO → α-LUSO+11 (81)
	442 (455)	0.0660	α-HOSO-5 → α-LUSO (28)<br>α-HOSO-3 → α-LUSO+2 (36)<br>α-HOSO-2 → α-LUSO+3 (34)<br>β-HOSO-4 → β-LUSO (31)<br>β-HOSO-3 → β-LUSO+3 (24)<br>β-HOSO-2 → β-LUSO+2 (32)<br>β-HOSO-2 → β-LUSO+3 (23)<br>β-HOSO-1 → β-LUSO+2 (23)
	428 (455)	0.1673	α-HOSO-5 → α-LUSO (23)<br>α-HOSO-3 → α-LUSO+2 (44)<br>α-HOSO-2 → α-LUSO+3 (28)<br>α-HOSO-1 → α-LUSO (25)<br>β-HOSO-4 → β-LUSO (23)<br>β-HOSO-2 → β-LUSO+2 (34)<br>β-HOSO-2 → β-LUSO+3 (27)<br>β-HOSO-1 → β-LUSO+3 (22)

Compound	Wavelength (nm) [a]	Osc. Str. (*f*)	Major contributions (%) [b]
	316 (344)	0.1474	α-HOSO-1 → α-LUSO+5 (25)<br>β-HOSO-4 → β-LUSO+4 (49)<br>β-HOSO-4 → β-LUSO+5 (64)
	315 (344)	0.246	α-HOSO-6 → α-LUSO+4 (50)<br>α-HOSO-1 → α-LUSO+5 (35)<br>β-HOSO-5 → β-LUSO+4 (35)<br>β-HOSO-4 → β-LUSO+5 (26)<br>β-HOSO-3 → β-LUSO+6 (23)<br>β-HOSO-1 → β-LUSO+6 (22)
	311 (344)	0.2664	α-HOSO-7 → α-LUSO+4 (25)<br>α-HOSO-6 → α-LUSO+4 (38)<br>α-HOSO-3 → α-LUSO+5 (20)<br>α-HOSO-1 → α-LUSO+5 (22)<br>β-HOSO-5 → β-LUSO+5 (59)<br>β-HOSO-2 → β-LUSO+6 (22)
	310 (344)	0.2113	α-HOSO-3 → α-LUSO+4 (29)<br>α-HOSO-6 → α-LUSO+5 (33)<br>α-HOSO-5 → α-LUSO+5 (22)<br>α-HOSO-3 → α-LUSO+5 (50)<br>β-HOSO-5 → β-LUSO+5 (25)<br>β-HOSO-2 → β-LUSO+6 (34)
	276 (277)	0.1196	α-HOSO-11 → α-LUSO+1 (26)<br>β-HOSO-13 → β-LUSO (20)<br>β-HOSO-11 → β-LUSO+1 (30)<br>β-HOSO-11 → β-LUSO+3 (24)
	275 (277)	0.1213	α-HOSO-15 → α-LUSO (22)<br>α-HOSO-14 → α-LUSO+1 (26)<br>α-HOSO-11 → α-LUSO+3 (20)<br>α-HOSO-7 → α-LUSO+4 (31)<br>β-HOSO-11 → β-LUSO+3 (21)<br>β-HOSO-6 → β-LUSO+4 (20)<br>β-HOSO-6 → β-LUSO+5 (23)<br>β-HOSO-2 → β-LUSO+8 (21)
	271 (277)	0.2778	β-HOSO-16 → β-LUSO (40)<br>β-HOSO-13 → β-LUSO (23)<br>β-HOSO-12 → β-LUSO+1 (29)
	270 (277)	0.1508	α-HOSO-5 → α-LUSO+7 (30)<br>α-HOSO-3 → α-LUSO+12 (29)<br>β-HOSO-5 → β-LUSO+12 (21)<br>β-HOSO-4 → β-LUSO+8 (32)<br>β-HOSO-2 → β-LUSO+12 (26)
	269.6 (277)	0.2120	α-HOSO-7 → α-LUSO+4 (22)<br>α-HOSO-2 → α-LUSO+12 (23)<br>β-HOSO-3 → β-LUSO+12 (20)<br>β-HOSO → β-LUSO+14 (21)
	269.4 (277)	0.2619	α-HOSO-5 → α-LUSO+12 (22)<br>α-HOSO-4 → α-LUSO+6 (21)<br>α-HOSO-3 → α-LUSO+13 (22)<br>α-HOSO-2 → α-LUSO+12 (30)<br>β-HOSO-4 → β-LUSO+12 (23)<br>β-HOSO-3 → β-LUSO+12 (24)

Compound	Wavelength (nm) [a]	Osc. Str. (*f*)	Major contributions (%) [b]
			β-HOSO-2 → β-LUSO+13 (20)<br>β-HOSO-1 → β-LUSO+12 (21)
	221 (225)	0.3211	α-HOSO-8 → α-LUSO+6 (60)<br>β-HOSO-7 → β-LUSO+7 (59)

[a] Corresponding experimental absorption in brackets. [b] See Figure S39.

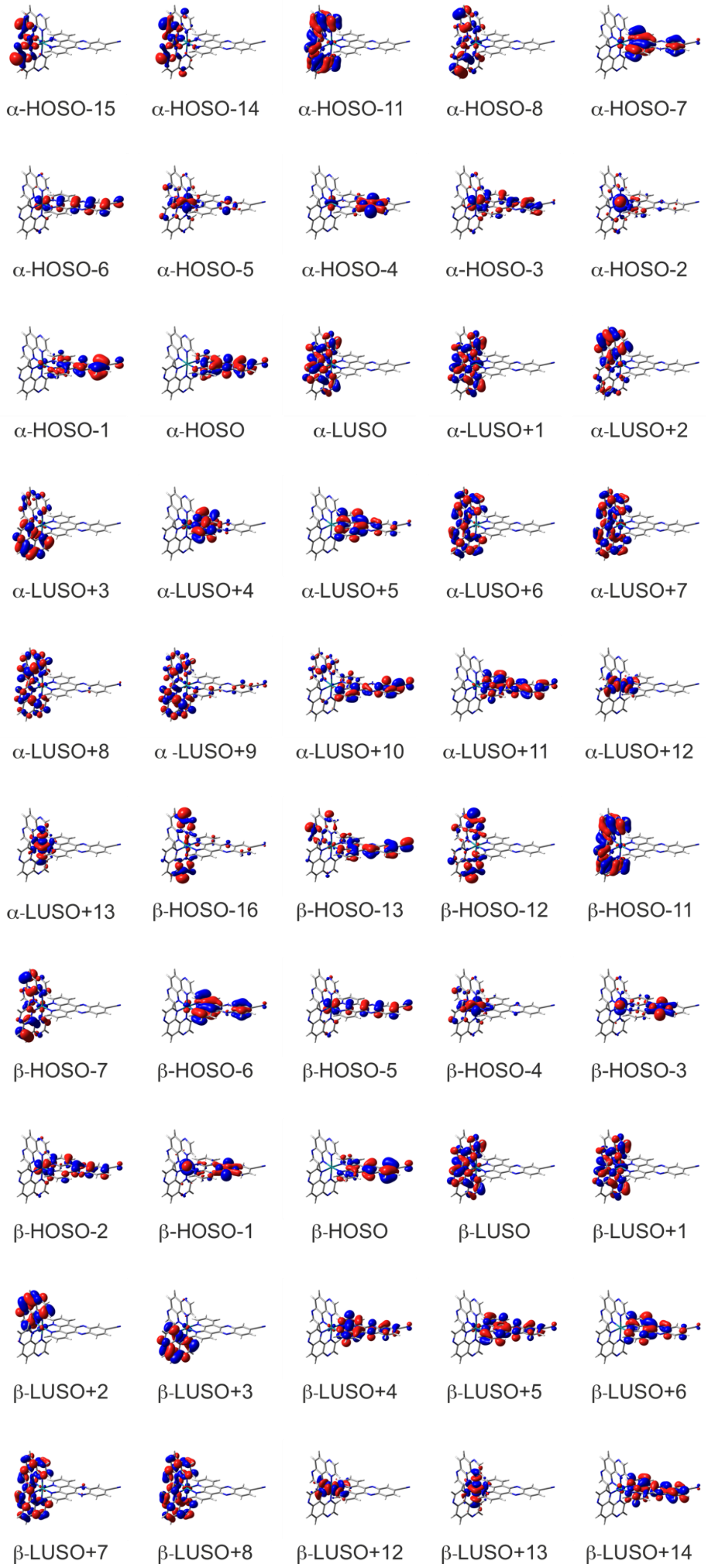

Figure S39. Molecular spin-orbitals involved in electronic transitions of $[Ru(tap)_2(11\text{-CN-dppz})]^+$ (listed in Table S6).

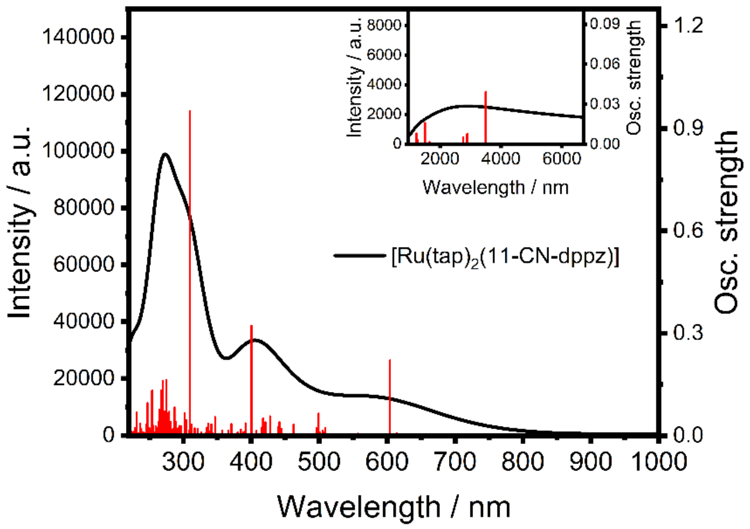

Figure S40. TDDFT-calculated UV-vis absorption spectrum of [Ru(tap)$_2$(11-CN-dppz)].

Table S7. Major electronic excitations in [Ru(tap)$_2$(11-CN-dppz)] determined by TDDFT calculations.

Compound	Wavelength (nm) [a]	Osc. Str. (*f*)	Major contributions (%) [b]
[Ru(tap)$_2$(11-CN-dppz)]	3500 (–)	0.0391	α-HOSO-1 → α-LUSO (99)<br>α-HOSO → α-LUSO (26)<br>α-HOSO-1 ← α-LUSO (22)
	1500 (–)	0.0160	α-HOSO → α-LUSO+2 (49)<br>α-HOSO → α-LUSO+3 (86)
	604 (647,591)	0.2212	α-HOSO → α-LUSO+5 (53)<br>α-HOSO → α-LUSO+6 (24)<br>α-HOSO → α-LUSO+7 (75)
	499 (467)	0.0651	α-HOSO-1 → α-LUSO+9 (25)<br>α-HOSO → α-LUSO+6 (30)<br>α-HOSO → α-LUSO+10 (81)
	400 (344)	0.3224	α-HOSO-4 → α-LUSO (20)<br>α-HOSO-3 → α-LUSO+3 (40)<br>β-HOSO-3 → β-LUSO+3 (34)<br>β-HOSO-2 → β-LUSO+2 (27)<br>β-HOSO-2 → β-LUSO+4 (53)<br>β-HOSO-2 → β-LUSO+5 (25)
	310 (277)	0.9512	α-HOSO-8 → α-LUSO+4 (21)<br>α-HOSO-5 → α-LUSO+4 (65)<br>β-HOSO-5 → β-LUSO+4 (30)<br>β-HOSO-5 → β-LUSO+5 (23)<br>β-HOSO → β-LUSO+6 (38)
	275 (277)	0.1634	α-HOSO-12 → α-LUSO (24)<br>α-HOSO-10 → α-LUSO+2 (22)<br>α-HOSO-2 → α-LUSO+6 (26)

Compound	Wavelength (nm) [a]	Osc. Str. (*f*)	Major contributions (%) [b]
			β-HOSO-10 → β-LUSO+1 (22)<br>β-HOSO-10 → β-LUSO+3 (25)<br>β-HOSO-8 → β-LUSO+2 (31)<br>β-HOSO-8 → β-LUSO+4 (28)<br>β-HOSO-1 → β-LUSO+8 (37)
	270 (277)	0.1604	α-HOSO-14 → α-LUSO+2 (31)<br>α-HOSO-13 → α-LUSO+1 (25)<br>α-HOSO-9 → α-LUSO+3 (25)<br>α-HOSO-8 → α-LUSO+3 (33)<br>β-HOSO-12 → β-LUSO+1 (25)<br>β-HOSO-9 → β-LUSO+4 (23)<br>β-HOSO-6 → β-LUSO+5 (20)<br>β-HOSO → β-LUSO+7 (28)
	269 (277)	0.1336	α-HOSO-3 → α-LUSO+6 (23)<br>α-HOSO-2 → α-LUSO+7 (55)<br>α-HOSO-2 → α-LUSO+10 (20)<br>β-HOSO-12 → β-LUSO (20)<br>β-HOSO-2 → β-LUSO+8 (20)<br>β-HOSO → β-LUSO+7 (27)
	267 (277)	0.1323	α-HOSO-9 → α-LUSO+3 (63)<br>α-HOSO-3 → α-LUSO+7 (21)<br>β-HOSO-10 → β-LUSO+4 (34)<br>β-HOSO-6 → β-LUSO+5 (25)
	254 (277)	0.1327	α-HOSO-5 → α-LUSO+5 (40)<br>α-HOSO-5 → α-LUSO+7 (29)<br>β-HOSO-16 → β-LUSO+4 (30)<br>β-HOSO-11 → β-LUSO+4 (31)<br>β-HOSO → β-LUSO+9 (43)
	218 (225)	0.1277	α-HOSO-20 → α-LUSO+1 (24)<br>α-HOSO-19 → α-LUSO+3 (29)<br>α-HOSO-9 → α-LUSO+5 (32)

[a] Corresponding experimental absorption in brackets. [b] See Figure S41.

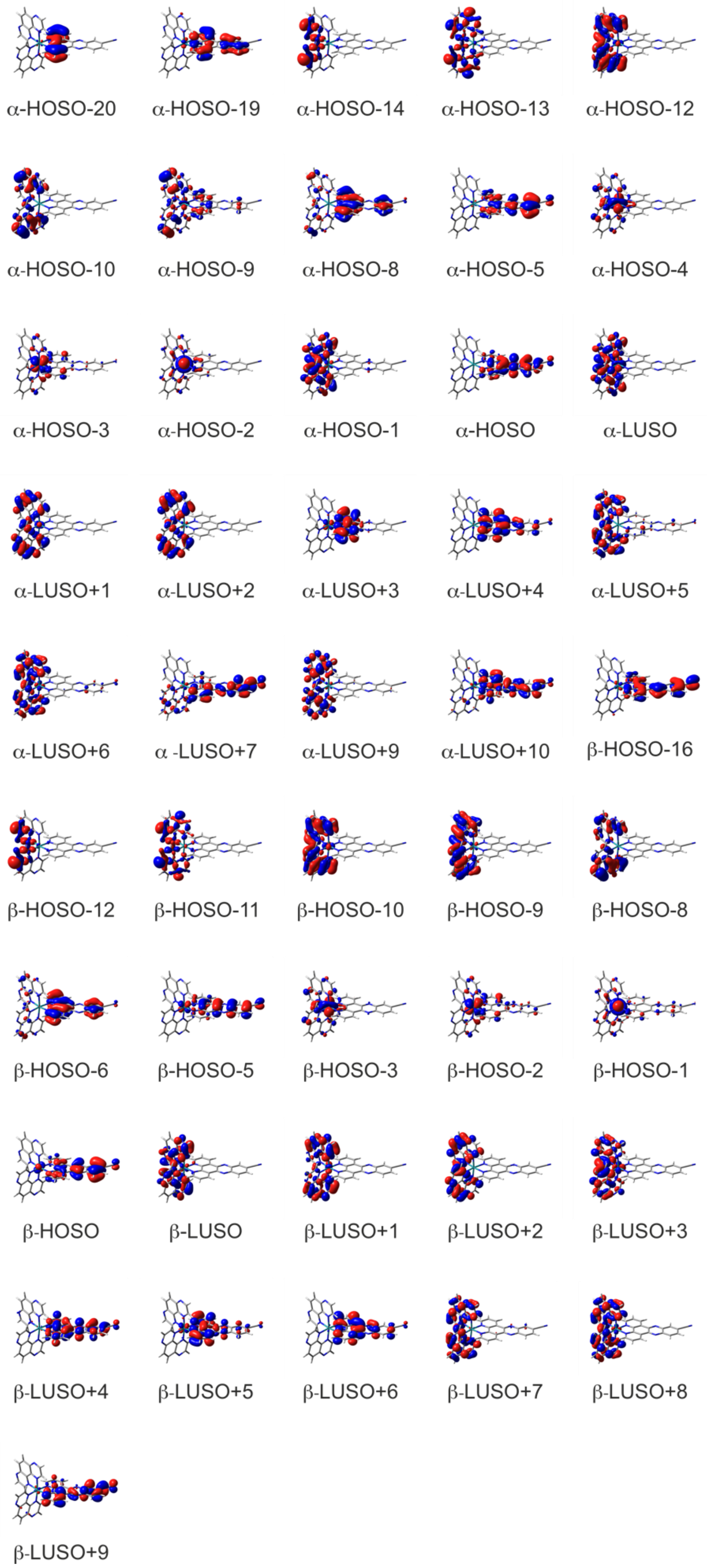

Figure S41. DFT-calculated molecular spin-orbitals involved in electronic transitions of [Ru(tap)$_2$(11-CN-dppz)] (listed in Table 7).

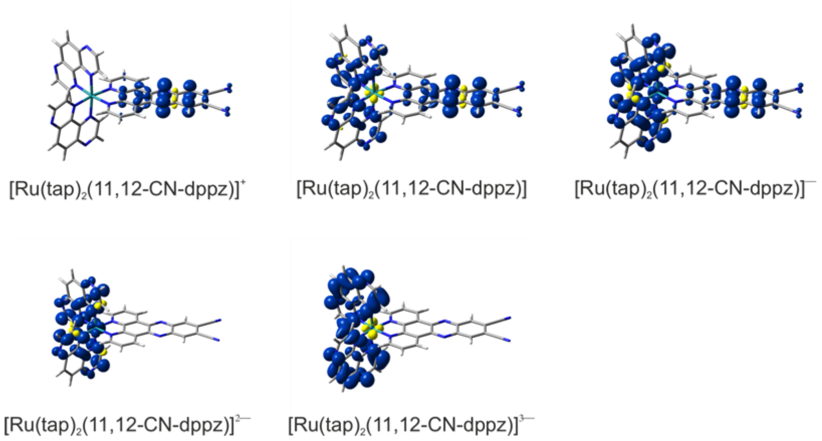

Figure S42. DFT-calculated spin density distributions in $[Ru(tap)_2(11,12\text{-}CN\text{-}dppz)]^{+/0/–/2–/3–}$.

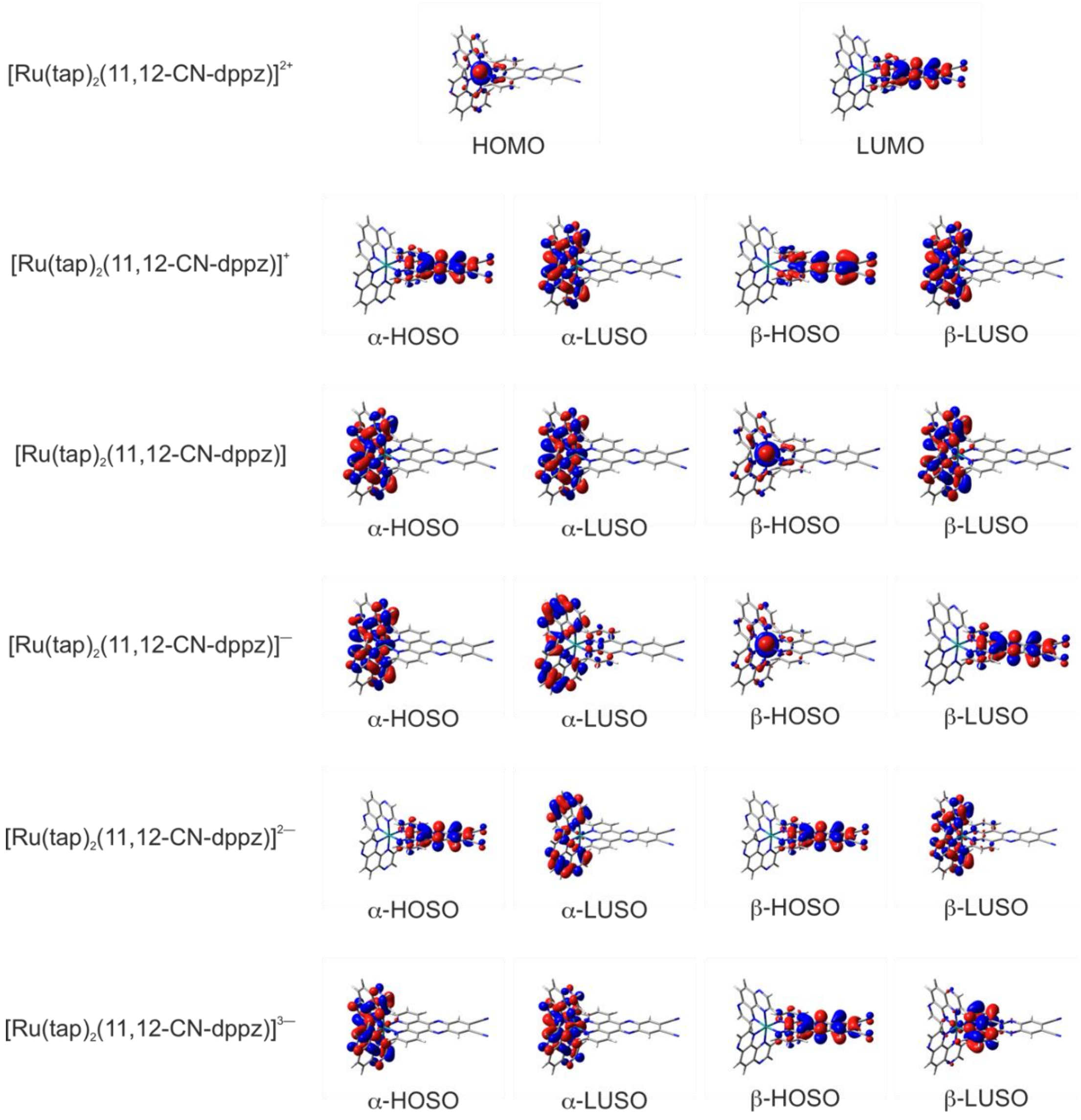

Figure S43. Frontier (spin-) orbitals of $[Ru(tap)_2(11,12\text{-}CN\text{-}dppz)]^{2+/+/0/–/2–/3–}$.

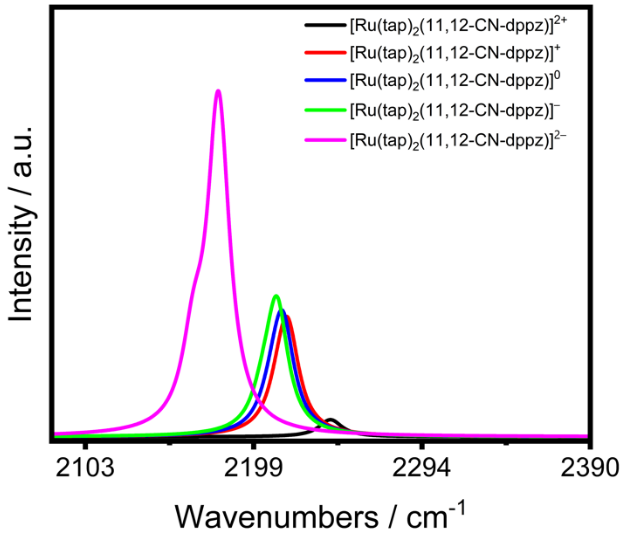

Figure S44. DFT-calculated IR spectra of $[Ru(tap_2(11,12\text{-}CN\text{-}dppz)]^{2+}$ (black curve), $[Ru(tap)_2(11,12\text{-}CN\text{-}dppz)]^+$ (red curve), $[Ru(tap)_2(11,12\text{-}CN\text{-}dppz)]$ (blue curve), $[Ru(tap)_2(11,12\text{-}CN\text{-}dppz)]^-$ (green curve) and $[Ru(tap)_2(11,12\text{-}CN\text{-}dppz)]^{2-}$ (purple curve).

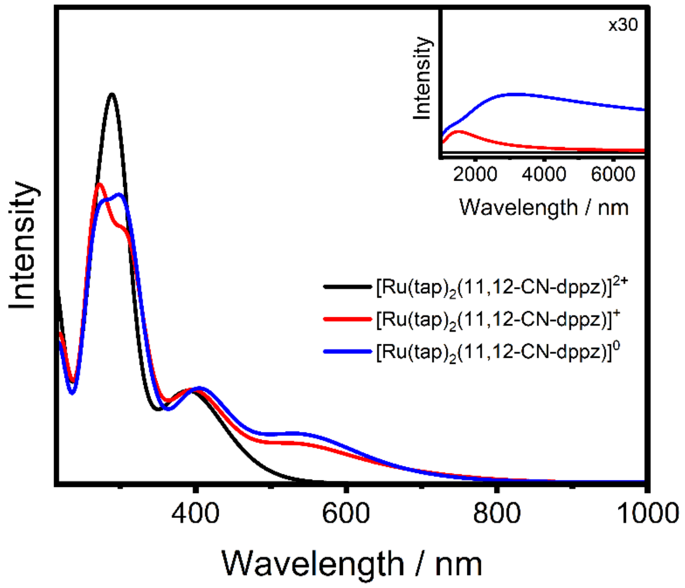

Figure S45. TDDFT-calculated UV-vis absorption spectra of $[Ru(tap)_2(11,12\text{-}CN\text{-}dppz)]^{2+}$ (black curve), $[Ru(tap)_2(11,12\text{-}CN\text{-}dppz)]^+$ (red curve) and $[Ru(tap)_2(11,12\text{-}CN\text{-}dppz)]$ (blue curve).

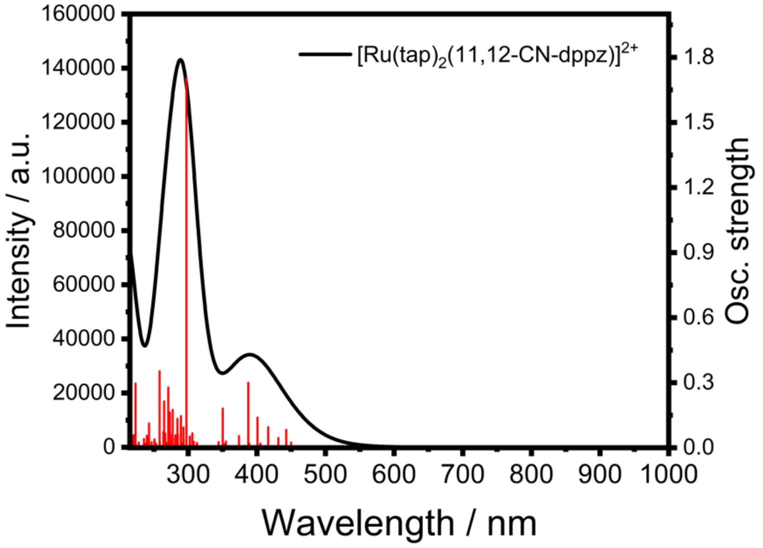

Figure S46. TDDFT-calculated UV-vis absorption spectrum of $[Ru(tap)_2(11,12\text{-CN-dppz})]^{2+}$.

Table S8. Major electronic excitations in $[Ru(tap)_2(11,12\text{-CN-dppz})]^{2+}$ determined by TDDFT calculations.

Compound	Wavelength (nm)[a]	Osc. Str. (*f*)	Major contributions (%) [b]
$[Ru(tap)_2(11,12\text{-CN-dppz})]^{2+}$	450 (457sh)	0.0244	HOMO-1 → LUMO+1 (65)
	443 (457sh)	0.0832	HOMO-1 → LUMO (67)
	431 (457sh)	0.0452	HOMO-2 → LUMO (25)<br>HOMO-2 → LUMO+1 (46)<br>HOMO-1 → LUMO+2 (28)<br>HOMO → LUMO+4 (35)
	417 (404)	0.0957	HOMO-2 → LUMO+1 (28)<br>HOMO-1 → LUMO+3 (31)<br>HOMO → LUMO+4 (54)
	405 (404)	0.0199	HOMO-2 → LUMO+2 (30)<br>HOMO-2 → LUMO+3 (26)<br>HOMO-1 → LUMO+4 (51)<br>HOMO → LUMO+3 (20)
	401 (404)	0.1396	HOMO-1 → LUMO+3 (61)<br>HOMO → LUMO+4 (21)
	388 (404)	0.2997	HOMO-2 → LUMO+2 (28)<br>HOMO-2 → LUMO+3 (35)<br>HOMO-1 → LUMO+4 (20)<br>HOMO-1 → LUMO+5 (44)
	350 (360)	0.1812	HOMO-4 → LUMO (60)<br>HOMO-3 → LUMO+6 (30)
	297 (282)	1.7053	HOMO-4 → LUMO (26)<br>HOMO-4 → LUMO+5 (21)<br>HOMO-3 → LUMO+6 (56)
	289 (282)	0.1466	HOMO-4 → LUMO+5 (48)

Compound	Wavelength (nm)[a]	Osc. Str. (*f*)	Major contributions (%) [b]
	284 (282)	0.1336	HOMO-9 → LUMO+4 (31)<br>HOMO-8 → LUMO+3 (43)<br>HOMO-2 → LUMO+8 (28)
	278 (282)	0.1748	HOMO-8 → LUMO+2 (26)<br>HOMO-8 → LUMO+3 (25)<br>HOMO-2 → LUMO+8 (28)
	273 (282)	0.1631	HOMO-9 → LUMO+2 (21)<br>HOMO-2 → LUMO+7 (20)<br>HOMO-1 → LUMO+8 (25)<br>HOMO-1 → LUMO+14 (26)<br>HOMO → LUMO+9 (23)
	271 (282)	0.2784	HOMO-2 → LUMO+8 (34)
	265 (282)	0.2146	HOMO-10 → LUMO+5 (21)<br>HOMO-9 → LUMO+5 (42)<br>HOMO-1 → LUMO+9 (41)
	258 (282)	0.3537	HOMO-10 → LUMO+5 (51)<br>HOMO → LUMO+10 (27)
	243 (282)	0.1124	HOMO-16 → LUMO (60)
	223 (230)	0.2965	HOMO-16 → LUMO+3 (23)<br>HOMO-5 → LUMO+8 (61)
	214 (230)	0.1316	HOMO-16 → LUMO+5 (21)<br>HOMO-6 → LUMO+9 (47)<br>HOMO-4 → LUMO+9 (31)

[a] Corresponding experimental absorption in brackets. [b] See Figure S47.

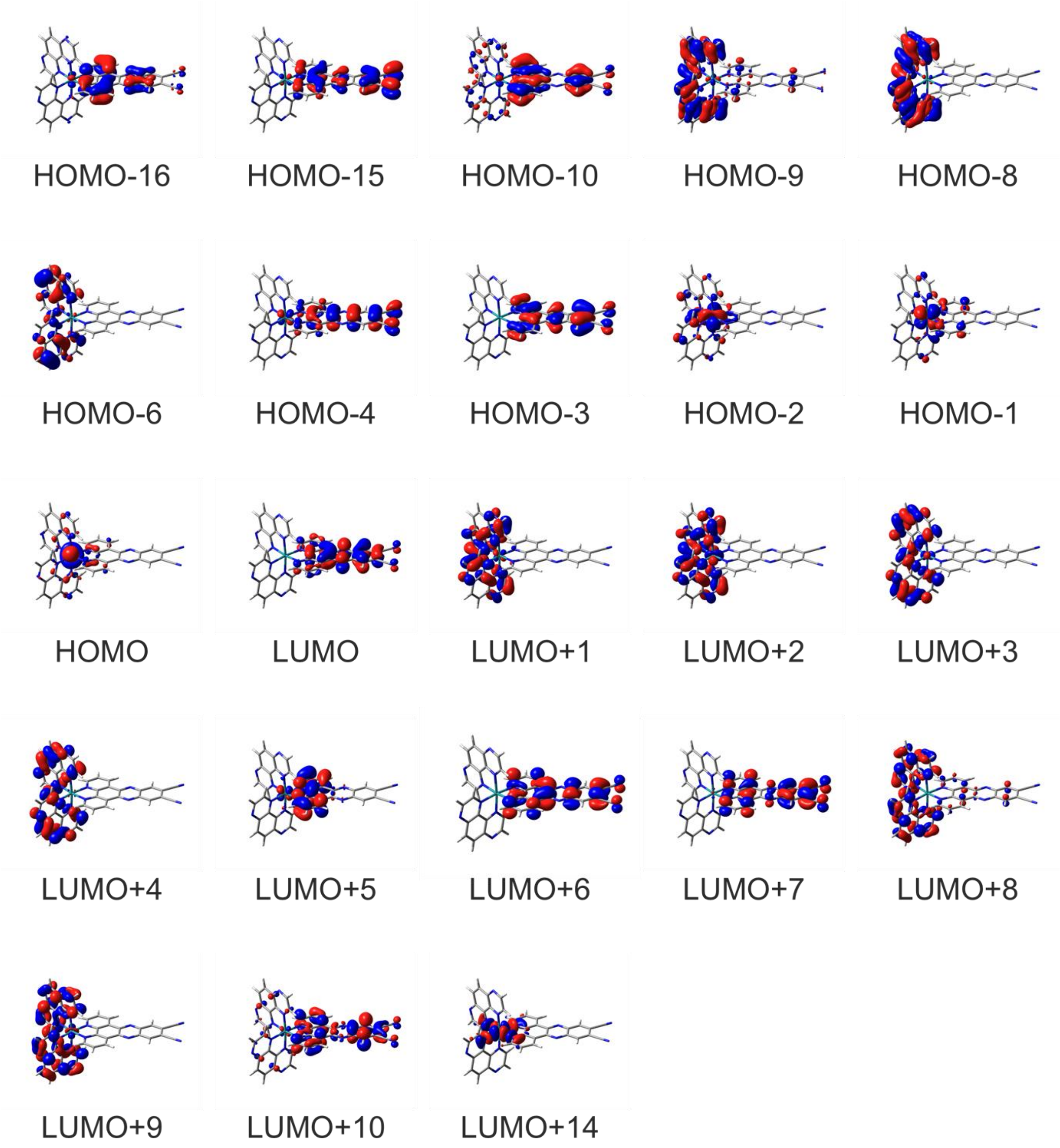

Figure S47. Molecular orbitals involved in electronic transitions of $[Ru(tap)_2(11,12\text{-CN-dppz})]^{2+}$ (listed in Table S8).

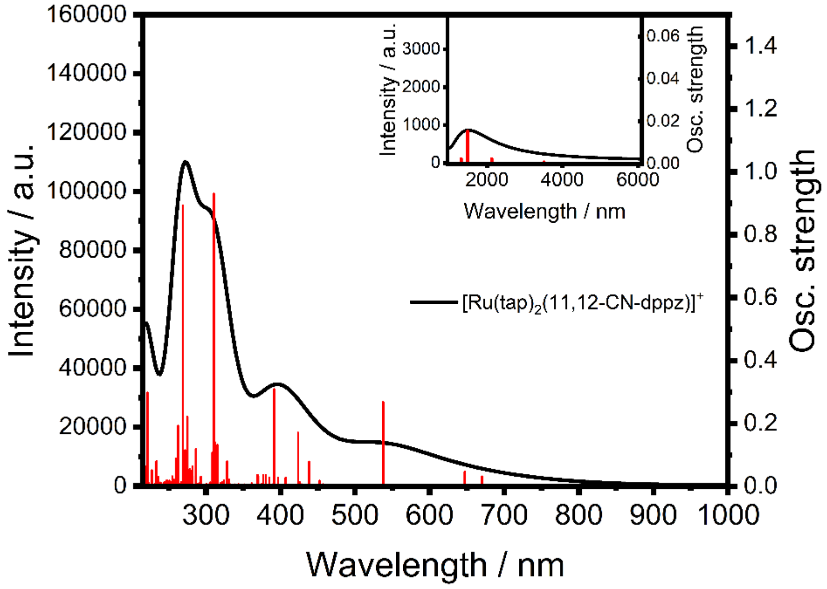

Figure S48. TDDFT-calculated UV-vis absorption spectrum of $[Ru(tap)_2(11,12\text{-}CN\text{-}dppz)]^+$.

Table S9 Major electronic excitations in [Ru(tap)$_2$(11,12-CN-dppz)]$^+$ determined by TDDFT calculations.

Compound	Wavelength (nm) ^{a}	Osc. Str. (f)	Major contributions (%) ^{b}
[Ru(tap)$_2$(11,12-CN-dppz)]$^+$	1480 (–)	0.0154	α-HOSO → α-LUSO+4 (99.5)
	670 (630)	0.0310	α-HOSO → α-LUSO+7 (25)<br>α-HOSO → α-LUSO+8 (94)
	647 (630)	0.0470	α-HOSO → α-LUSO+6 (99)
	538 (580)	0.2682	α-HOSO → α-LUSO+10 (84)<br>α-HOSO → α-LUSO+11 (46)
	438 (460)	0.0783	α-HOSO-4 → α-LUSO (30)<br>α-HOSO-2 → α-LUSO+2 (35)<br>α-HOSO-1 → α-LUSO+3 (41)<br>β-HOSO-4 → β-LUSO (31)<br>β-HOSO-2 → β-LUSO+2 (43)<br>β-HOSO-1 → β-LUSO+3 (40)
	423 (460)	0.1703	α-HOSO-4 → α-LUSO (22)<br>α-HOSO-2 → α-LUSO+2 (56)<br>α-HOSO-1 → α-LUSO+3 (27)<br>β-HOSO-4 → β-LUSO (22)<br>β-HOSO-2 → β-LUSO+2 (44)<br>β-HOSO-1 → β-LUSO+3 (31)<br>β-HOSO → β-LUSO+3 (31)
	391 (404)	0.3091	α-HOSO-4 → α-LUSO+1 (33)<br>α-HOSO-4 → α-LUSO+2 (23)<br>α-HOSO-3 → α-LUSO+1 (27)<br>α-HOSO-1 → α-LUSO+2 (22)<br>β-HOSO-5 → β-LUSO (20)<br>β-HOSO-4 → β-LUSO+1 (30)<br>β-HOSO-4 → β-LUSO+2 (22)<br>β-HOSO-3 → β-LUSO+1 (31)<br>β-HOSO-2 → β-LUSO (24)<br>β-HOSO-2 → β-LUSO+3 (21)<br>β-HOSO-2 → β-LUSO+4 (26)<br>β-HOSO-2 → β-LUSO+5 (21)<br>β-HOSO-1 → β-LUSO+2 (22)
	315 (344)	0.1319	α-HOSO-5 → α-LUSO+5 (35)<br>α-HOSO-4 → α-LUSO+5 (63)<br>α-HOSO-3 → α-LUSO+5 (24)<br>α-HOSO-1 → α-LUSO+13 (24)<br>β-HOSO-1 → β-LUSO+14 (23)
	312 (344)	0.1400	α-HOSO → α-LUSO+17 (92)
	311 (344)	0.9306	α-HOSO-4 → α-LUSO+5 (30)<br>α-HOSO-3 → α-LUSO+5 (50)<br>α-HOSO → α-LUSO+17 (26)<br>α-HOSO → α-LUSO+19 (41)<br>β-HOSO-5 → β-LUSO+4 (33)<br>β-HOSO-4 → β-LUSO+6 (23)<br>β-HOSO → β-LUSO+6 (32)
	309 (344)	0.1005	α-HOSO → α-LUSO+17 (20)<br>α-HOSO → α-LUSO+19 (78)

Compound	Wavelength (nm) [a]	Osc. Str. (*f*)	Major contributions (%) [b]
			β-HOSO-4 → β-LUSO+6 (41)
	308 (344)	0.1066	α-HOSO-6 → α-LUSO+4 (27)<br>α-HOSO-4 → α-LUSO+5 (20)<br>α-HOSO-3 → α-LUSO+5 (20)<br>α-HOSO → α-LUSO+19 (35)<br>β-HOSO-5 → β-LUSO+4 (21)<br>β-HOSO-4 → β-LUSO+6 (69)
	286 (280)	0.1192	α-HOSO-11 → α-LUSO+3 (25)<br>α-HOSO-10 → α-LUSO+2 (32)<br>α-HOSO-4 → α-LUSO+6 (46)<br>β-HOSO-10 → β-LUSO+3 (26)<br>β-HOSO-9 → β-LUSO+2 (33)<br>β-HOSO-4 → β-LUSO+7 (46)<br>β-HOSO-3 → β-LUSO+7 (21)
	275 (280)	0.2212	α-HOSO-11 → α-LUSO+1 (37)<br>α-HOSO-11 → α-LUSO+2 (25)<br>α-HOSO-9 → α-LUSO+3 (21)<br>α-HOSO-1 → α-LUSO+7 (22)<br>β-HOSO-10 → β-LUSO+1 (37)<br>β-HOSO-10 → β-LUSO+2 (23)<br>β-HOSO-8 → β-LUSO+3 (21)<br>β-HOSO-1 → β-LUSO+8 (22)
	272 (280)	0.1146	α-HOSO-16 → α-LUSO (34)<br>α-HOSO-12 → α-LUSO+1 (29)<br>α-HOSO-7 → α-LUSO+4 (27)<br>α-HOSO-3 → α-LUSO+14 (24)<br>β-HOSO-17 → β-LUSO+4 (23)
	269 (280)	0.8927	α-HOSO-11 → α-LUSO (20)<br>α-HOSO-10 → α-LUSO+1 (25)<br>α-HOSO-7 → α-LUSO+4 (42)<br>β-HOSO-10 → β-LUSO (20)<br>β-HOSO-9 → β-LUSO+1 (25)<br>β-HOSO-6 → β-LUSO+5 (34)
	262 (280)	0.1923	α-HOSO-7 → α-LUSO+5 (37)<br>α-HOSO-3 → α-LUSO+8 (41)<br>α-HOSO-1 → α-LUSO+8 (42)<br>β-HOSO-6 → β-LUSO+6 (25)<br>β-HOSO → β-LUSO+9 (39)
	221 (227)	0.2974	α-HOSO-8 → α-LUSO+6 (62)<br>β-HOSO-7 → β-LUSO+7 (59)
	215 (227)	0.1388	α-HOSO-8 → α-LUSO+7 (59)<br>β-HOSO-7 → β-LUSO+8 (60)

[a] Corresponding experimental absorption in brackets. [b] See Figure S49.

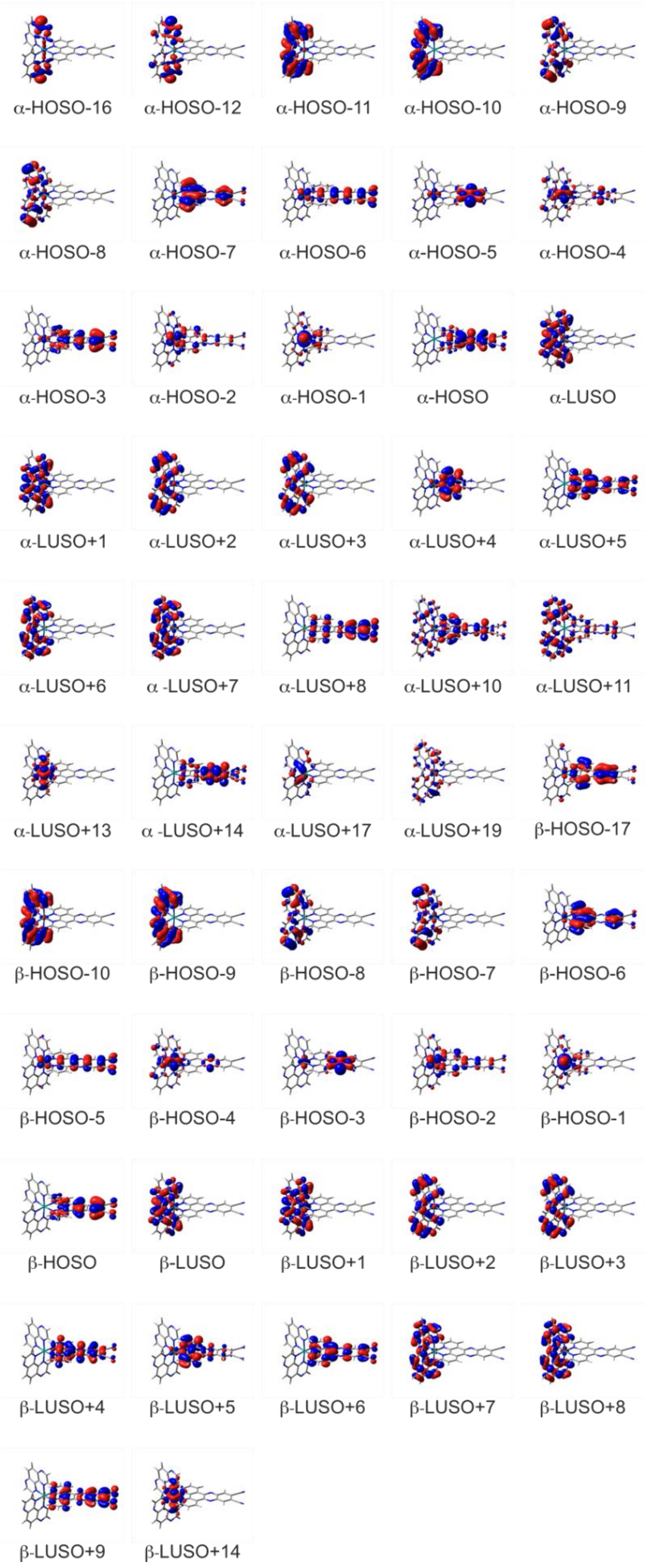

Figure S49. Molecular spin-orbitals involved in electronic transitions of $[Ru(tap)_2(11,12\text{-}CN\text{-}dppz)]^+$ (listed in Table S9).

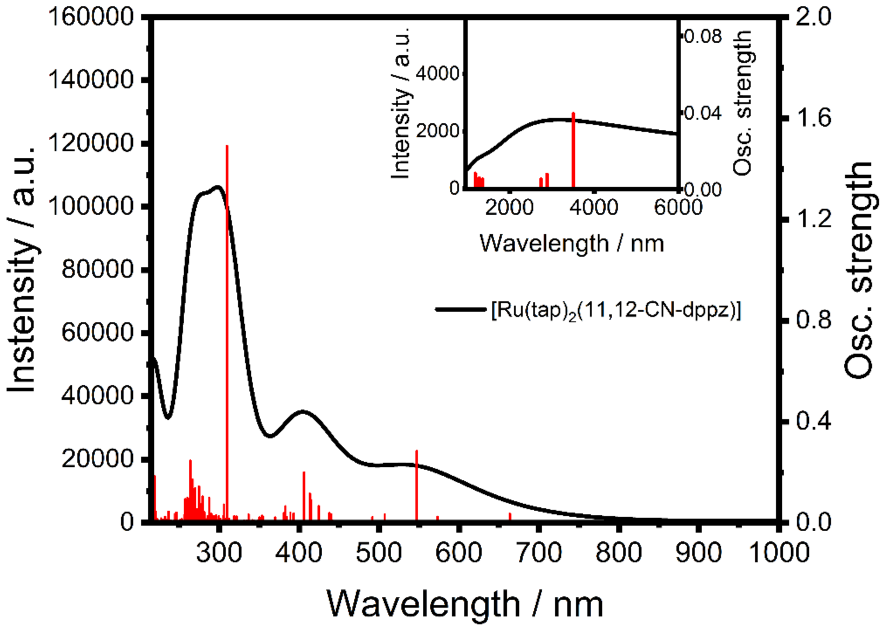

Figure S50. TDDFT-calculated UV-vis absorption spectrum of $[Ru(tap)_2(11,12\text{-}CN\text{-}dppz)]$.

Table S10. Major electronic excitations in [Ru(tap)$_2$(11,12-CN-dppz)] determined by TDDFT calculations.

Compound	Wavelength (nm) [a]	Osc. Str. (*f*)	Major contributions (%) [b]
[Ru(tap)$_2$(11,12-CN-dppz)]	3504 (–)	0.0395	α-HOSO → α-LUSO (102)<br>α-HOSO ← α-LUSO (23)
	663 (630)	0.0361	α-HOSO-1 → α-LUSO+5 (97)
	573 (580)	0.0242	α-HOSO-4 → α-LUSO (86)<br>β-HOSO-3 → β-LUSO+4 (28)<br>β-HOSO-2 → β-LUSO+4 (20)
	547 (580)	0.284	α-HOSO-1 → α-LUSO+6 (70)<br>α-HOSO-1 → α-LUSO+8 (67)
	424 (460)	0.0660	α-HOSO-4 → α-LUSO+2 (22)<br>α-HOSO-3 → α-LUSO+1 (25)<br>α-HOSO-2 → α-LUSO+2 (20)<br>β-HOSO-1 → β-LUSO+4 (23)<br>β-HOSO → β-LUSO+3 (84)
	415 (460)	0.0904	α-HOSO-3 → α-LUSO+1 (33)<br>β-HOSO-1 → β-LUSO+2 (66)<br>β-HOSO-1 → β-LUSO+4 (56)
	413 (460)	0.1153	α-HOSO-3 → α-LUSO+3 (24)<br>β-HOSO-3 → β-LUSO+2 (34)<br>β-HOSO-2 → β-LUSO+2 (24)<br>β-HOSO-1 → β-LUSO+1 (49)<br>β-HOSO-1 → β-LUSO+3 (60)
	406 (460)	0.1991	α-HOSO-3 → α-LUSO+3 (29)<br>β-HOSO-1→ β-LUSO+1 (56)<br>β-HOSO-1 → β-LUSO+3 (63)
	310 (344)	1.4906	α-HOSO-7 → α-LUSO+2 (23)<br>α-HOSO-5 → α-LUSO+4 (69)<br>β-HOSO-5 → β-LUSO+1 (36)<br>β-HOSO-5 → β-LUSO+5 (21)<br>β-HOSO-3 → β-LUSO+6 (25)<br>β-HOSO-2 → β-LUSO+6 (34)
	279 (281)	0.1055	α-HOSO-12 → α-LUSO+2 (51)<br>α-HOSO-11 → α-LUSO+1 (27)<br>α-HOSO-10 → α-LUSO+2 (38)<br>β-HOSO-1 → β-LUSO+7 (25)
	275 (281)	0.1437	α-HOSO-12 → α-LUSO (22)<br>α-HOSO-9 → α-LUSO+2 (22)<br>α-HOSO-2 → α-LUSO+7 (30)<br>β-HOSO-10 → β-LUSO+2 (23)<br>β-HOSO-10 → β-LUSO+4 (23)<br>β-HOSO-7 → β-LUSO+3 (29)<br>β-HOSO-7 → β-LUSO+5 (23)<br>β-HOSO → β-LUSO+9 (43)
	270 (281)	0.1357	α-HOSO-3 → α-LUSO+7 (31)<br>α-HOSO-2 → α-LUSO+8 (22)<br>β-HOSO-12 → β-LUSO (25)<br>β-HOSO-11 → β-LUSO+2 (22)<br>β-HOSO-10 → β-LUSO+5 (23)

Compound	Wavelength (nm) [a]	Osc. Str. (*f*)	Major contributions (%) [b]
			β-HOSO-1 → β-LUSO+9 (46)
	267 (281)	0.1720	α-HOSO-7 → α-LUSO+5 (28)
			α-HOSO-5 → α-LUSO+11 (24)
			α-HOSO-3 → α-LUSO+5 (24)
			α-HOSO-1 → α-LUSO+23 (22)
			β-HOSO-17 → β-LUSO+1 (34)
			β-HOSO-8 → β-LUSO+5 (33)
			β-HOSO-5 → β-LUSO+7 (27)
	264 (281)	0.2459	α-HOSO-12 → α-LUSO+3 (27)
			α-HOSO-10 → α-LUSO+3 (40)
			β-HOSO-10 → β-LUSO+5 (35)
			β-HOSO-8 → β-LUSO+5 (24)
			β-HOSO-1 → β-LUSO+7 (25)
			β-HOSO → β-LUSO+10 (28)
	219 (227)	0.1842	α-HOSO-8 → α-LUSO+6 (53)
			β-HOSO-6 → β-LUSO+8 (61)

[a] Corresponding experimental absorption in brackets. [b] See Figure S51.

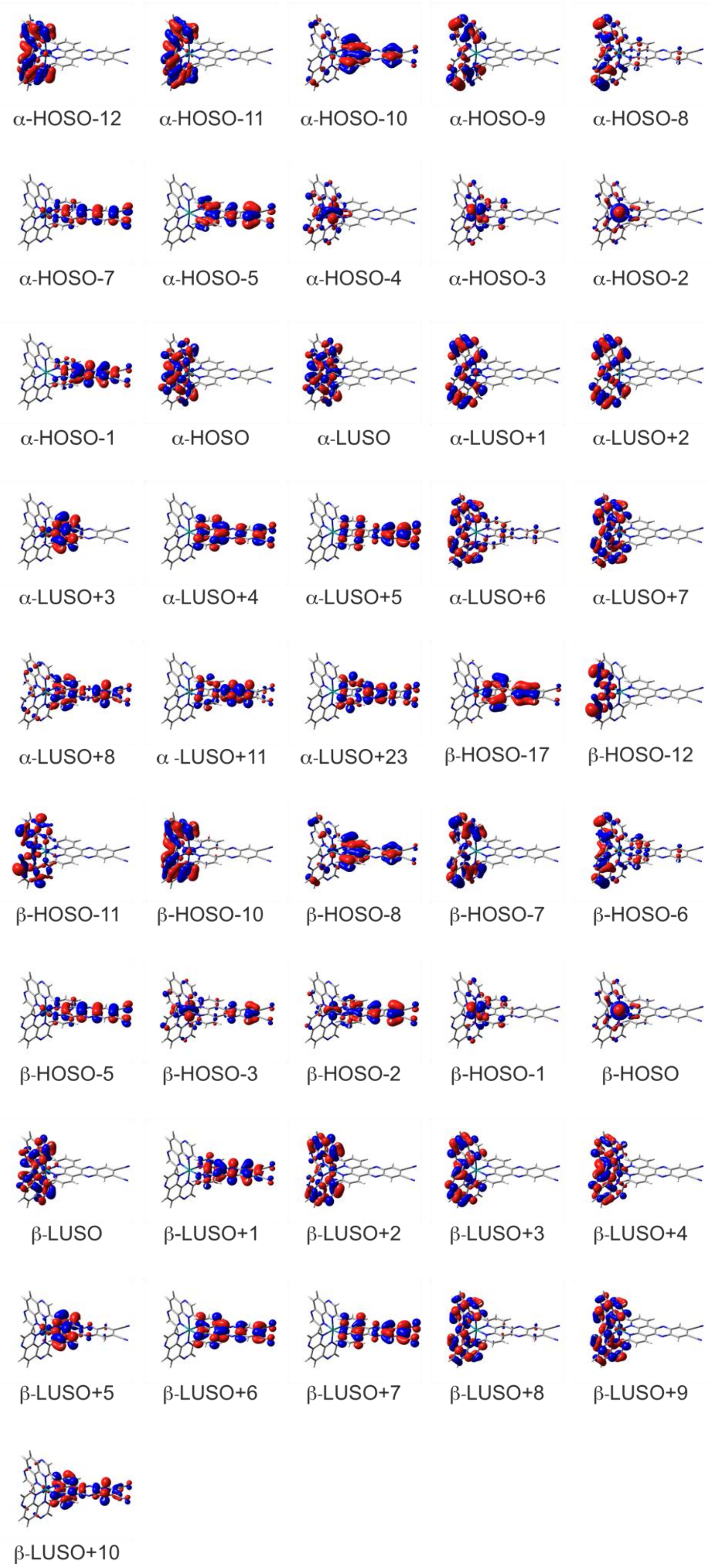

Figure S51. Molecular spin-orbitals involved in electronic transitions of [Ru(tap)$_2$(11,12-CN-dppz)] (listed in Table S10).

9. DFT-calculated charge-transfer excited-state properties of studied complexes

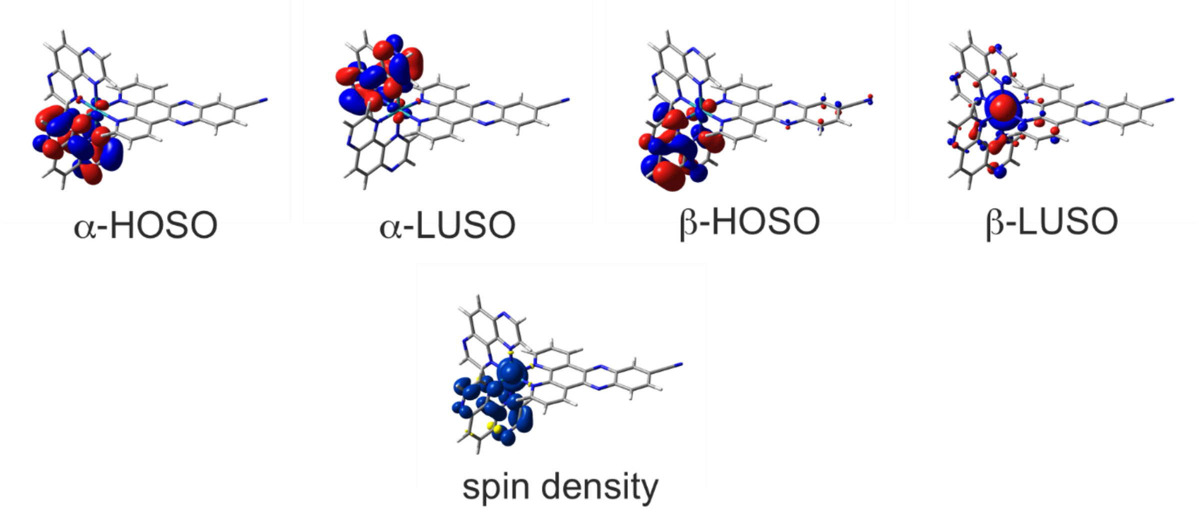

Figure S52. Frontier molecular spin-orbitals and spin density distribution in the lowest ^{3}MLCT (Ru→tap) excited state of [Ru(tap)$_2$(11-CN-dppz)]$^{2+}$.

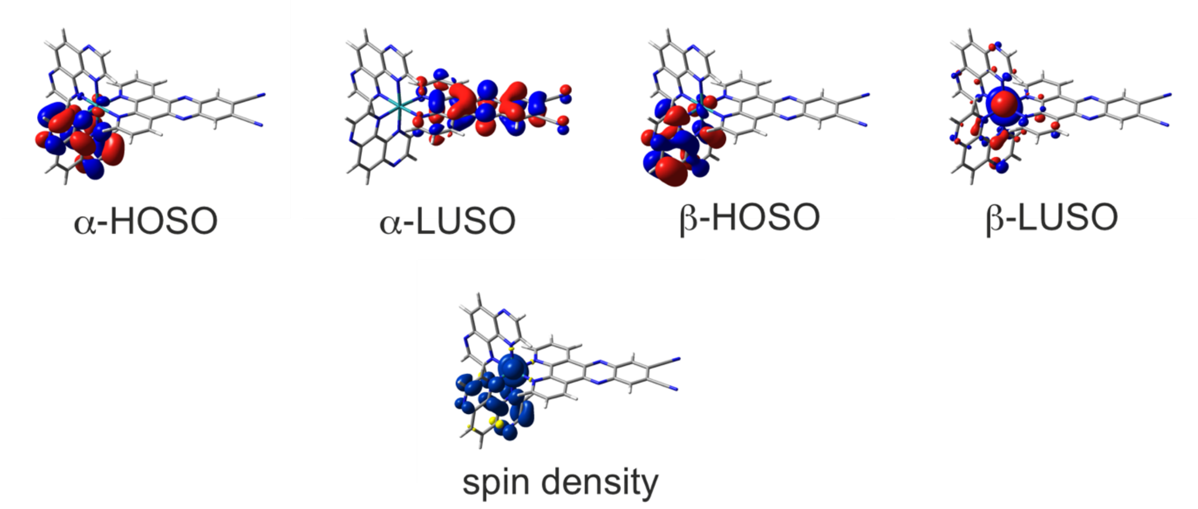

Figure S53. Frontier molecular spin-orbitals and spin density distribution in the lowest ^{3}MLCT (Ru→tap) excited state of [Ru(tap)$_2$(11,12-CN-dppz)]$^{2+}$.
